# One-Pot Synthesis of Styrene Derivatives from Allyl Silanes via B(C_6_F_5_)_3_-Catalyzed Isomerization–Hiyama Coupling

**DOI:** 10.1021/acs.orglett.2c03584

**Published:** 2022-11-17

**Authors:** Betty A. Kustiana, Rebecca L. Melen, Louis C. Morrill

**Affiliations:** Cardiff Catalysis Institute, School of Chemistry, Cardiff University, Main Building, Park Place, Cardiff CF10 3AT, U.K.

## Abstract

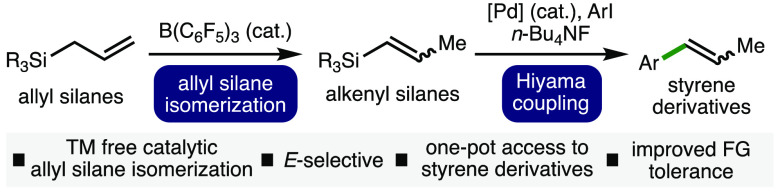

Herein, we report a one-pot synthesis of styrene derivatives via a novel B(C_6_F_5_)_3_-catalyzed *E*-selective isomerization of readily accessible allyl silanes and subsequent Hiyama coupling of the versatile alkenyl silane intermediates. This one-pot, two-step approach enables access to a broad range of styrene derivatives, including those containing Lewis basic functional groups, that cannot be accessed via the previously developed B(C_6_F_5_)_3_-catalyzed isomerization of allyl benzenes.

Alkenyl silanes are useful building blocks in organic synthesis, polymer chemistry, and materials science.^1^ They participate in a diverse array of transformations, including electrophilic substitution,^2^ polymerization,^3^ and cross-coupling reactions.^4^ Alkenyl silanes can be accessed by various methods, including nucleophilic substitution of chlorosilanes with alkenyl magnesium reagents,^5^ transition metal-catalyzed hydrosilylation of alkynes^6^ and allenes,^7^ dehydrogenative silylation of alkenes,^8^ and Cu-catalyzed silylation of alkenyl iodonium salts (Scheme 1A).^9^ An attractive alternative approach for the formation of substituted alkenyl silanes is the isomerization of allyl silanes, due to their relative ease of synthesis and commercial availability.^10^ A variety of catalytic approaches for the isomerization of allyl silanes to alkenyl silanes have been developed, which employ catalysts based on both precious metals (e.g., Ru, Pd, and Ir)^11^ and more abundant first-row transition metals (e.g., Fe, Co, and Ni).^12^

**Scheme 1:**
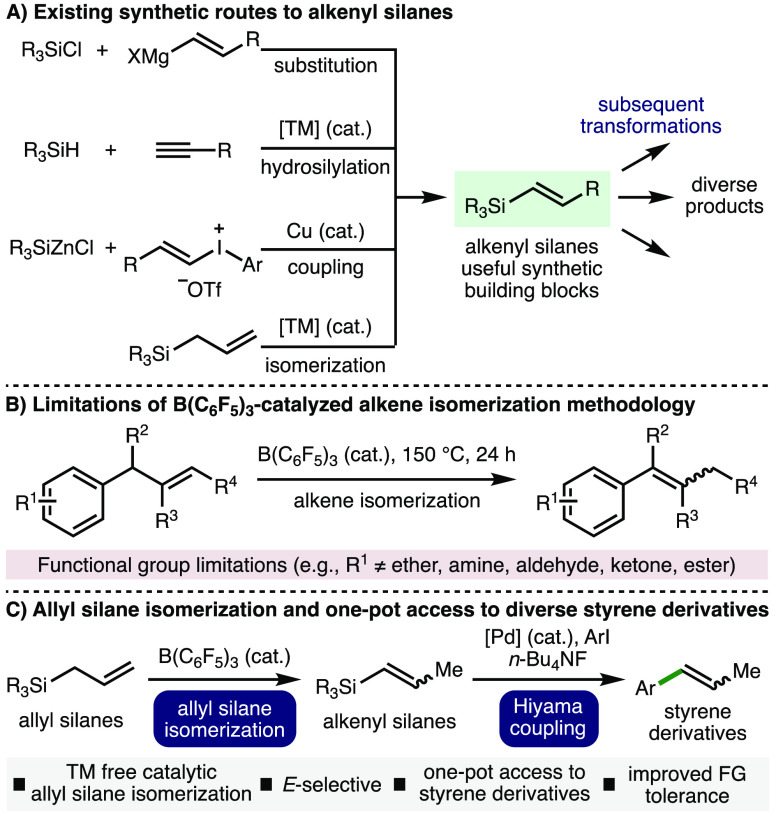
Context

The development and application of main group catalysts in synthesis continues to be an active area of investigation in organic chemistry.^13^ This can be attributed to the desire to understand further the reactivity and capabilities of main group catalysts, combined with the increasing drive to reduce the dependence upon finite precious metals.^14^ Among main group catalysts, fluorinated triarylboranes such as commercially available B(C_6_F_5_)_3_ have garnered significant attention.^15^ These species have been employed as catalysts in a variety of transformations, including hydrosilylation, frustrated Lewis pair (transfer) hydrogenation, and various C–C bond-forming reactions.^16^ As part of our ongoing interest in the use of boranes as catalysts in synthesis,^17^ we recently reported the B(C_6_F_5_)_3_-catalyzed *E*-selective isomerization of alkenes (Scheme 1B).^18^ Although the method could be applied across a broad range of alkene-containing substrates, the high Lewis acidity of B(C_6_F_5_)_3_ resulted in a number of limitations with respect to the incorporation of Lewis basic functional groups (e.g., ethers, amines, aldehydes, ketones, and esters). To address these limitations, herein we report the B(C_6_F_5_)_3_-catalyzed isomerization of allyl silanes to alkenyl silanes, which undergo Hiyama coupling in a one-pot, two-step process to access a more diverse array of valuable substituted styrene derivatives (Scheme 1C). Examples of biologically active molecules that contain substituted styrene motifs include anethole (food additive), isoeugenol (fragrance), and licarin A (antimycobacterial).

To commence our studies, the B(C_6_F_5_)_3_-catalyzed isomerization of allyl triphenyl silane **1** to form triphenyl(prop-1-en-1-yl)silane **2** was selected for reaction optimization (Table 1).^19^ Employing commercially available B(C_6_F_5_)_3_ (5 mol %) as a catalyst and toluene ([**1**] = 0.25 M) as a solvent in a sealed tube at 140 °C for 48 h under argon gave **2** in 85% NMR yield (80% isolated yield) with high selectivity for the *E*-alkene isomer (97:3 *E*:*Z*) (entry 1). No alkene isomerization was observed in the absence of B(C_6_F_5_)_3_ (entry 2). Decreasing the reaction time or the reaction temperature each reduced the NMR yield of **2** (entries 3 and 4), as did variation of the concentration and solvent (entries 5–8). Decreasing the catalyst loading to 2.5 mol % resulted in only 21% conversion to **2** (entry 9).

**Table 1:**
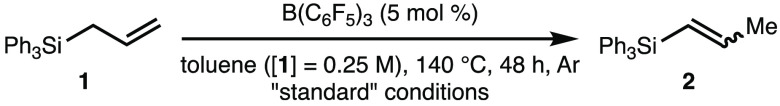
Reaction Optimization^a^

entry	variation from “standard” conditions	yield^b^ (%)	*E*:*Z* ratio^b^
1	none	85 (80)	97:3
2	no B(C_6_F_5_)_3_	<2	–
3	reaction time of 24 h	72	>98:<2
4	130 °C	6	84:16
5	[**1**] = 0.1 M	65	98:2
6	[**1**] = 0.5 M	72	94:6
7	chlorobenzene as the solvent	76	96:4
8	xylenes as the solvent	30	>98:<2
9	B(C_6_F_5_)_3_ (2.5 mol %)	21	>98:<2

a Reactions performed using 0.1 mmol of **1**.

b Determined by ^1^H NMR analysis of the crude reaction mixture using 1,3,5-trimethylbenzene as the internal standard. Isolated yield in parentheses.

With the optimized reaction conditions in hand, the scope of the B(C_6_F_5_)_3_-catalyzed allyl isomerization process was explored (Scheme 2). It was found that various aryl/alkyl substitutions on silicon were tolerated, which provided access to the corresponding internal alkene products in high yields (≤91%), and with good selectivity for the *E*-alkene isomer (products **2–13**). Commonly employed silicon-based protecting groups could be incorporated into the products, including *tert*-butyldiphenylsilyl (TBDPS) **3**, *tert*-butyldimethylsilyl (TBS) **8**, triisopropylsilyl (TIPS) **12**, and trimethylsilyl (TMS) **13**. The reaction performed well on a 2 mmol scale, which gave **7** in 80% yield and with 93:7 *E*:*Z* selectivity. It was found that allyltriphenylgermane and allyltriethylgermane also underwent B(C_6_F_5_)_3_-catalyzed isomerization to form products **14** and **15** in 58% and 45% yields, respectively.

**Scheme 2:**
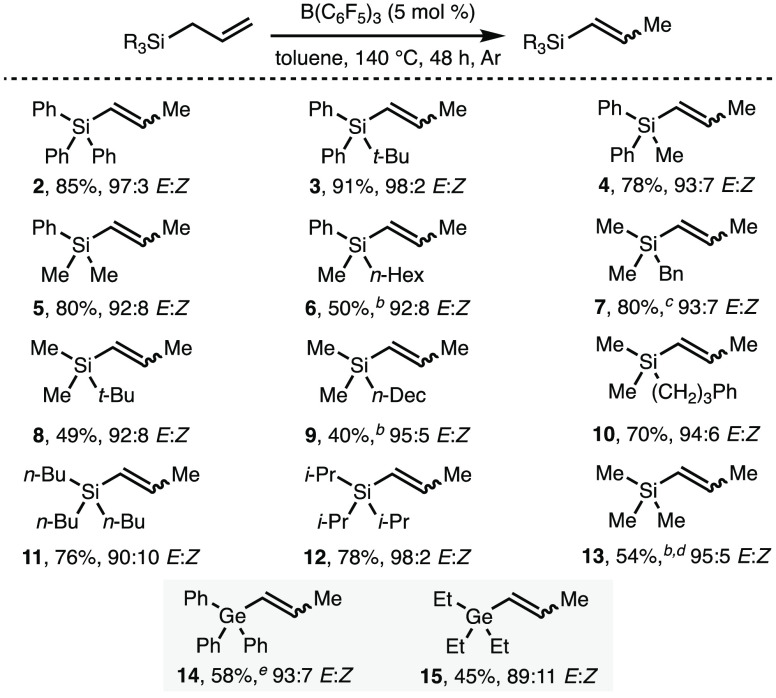
Scope of Allyl Silane Isomerization Reactions performed using 0.1 mmol of allyl silane. Yields determined by ^1^H NMR analysis of the crude reaction mixture using 1,3,5-trimethylbenzene as the internal standard. At 130 °C. With 2 mmol of substrate. At 24 h. At 72 h.

Having established the scope of the B(C_6_F_5_)_3_-catalyzed isomerization of allyl silanes, we investigated the synthetic utility of the corresponding prop-1-en-1-yl silane products. Of particular interest was the Pd-catalyzed Hiyama coupling between prop-1-en-1-yl silanes and aryl iodides,^4^ as it was envisaged that this strategy would generate substituted styrene derivatives that could not be accessed using our previously developed B(C_6_F_5_)_3_-catalyzed isomerization of allyl benzenes.^18^ Employing benzyldimethylsilane **7**,^20^ alkene isomerization was followed by Hiyama coupling via the addition of Pd(dba)_2_ (4 mol %), *n*-Bu_4_NF (2 equiv), and the desired aryl iodide (1 equiv) to the same reaction vessel, which was heated at 40 °C for 24 h under N_2_ (Scheme 3A). This one-pot, two-step process provided access to a broad range of substituted styrene derivatives in good yields with high *E* selectivity, bearing various functional groups, including ethers, amines, acetals, esters, nitriles, ketones, and sulfonamides (Scheme 3B, products **16–27**). Alkenyl-substituted heterocycles, including indole, benzofuran, and pyridine, were also formed in good yields with high *E* selectivity (products **28–31**). A majority of these products could not be accessed using our previously developed B(C_6_F_5_)_3_-catalyzed alkene isomerization methodology due to several competing processes, including the coordination of B(C_6_F_5_)_3_ to basic functionalities (e.g., pyridines), B(C_6_F_5_)_3_-mediated C–H hydride abstraction (e.g., benzylic and α-amino positions), and undesired reduction of susceptible functional groups (e.g., ketones). As an alternative demonstration of alkenyl silane derivatization, the stereospecific epoxidation of prop-1-en-1-yl silane **2** with mCPBA gave the corresponding *trans*-epoxide **32** as a single observable isomer in 65% isolated yield (Scheme 3C).

**Scheme 3:**
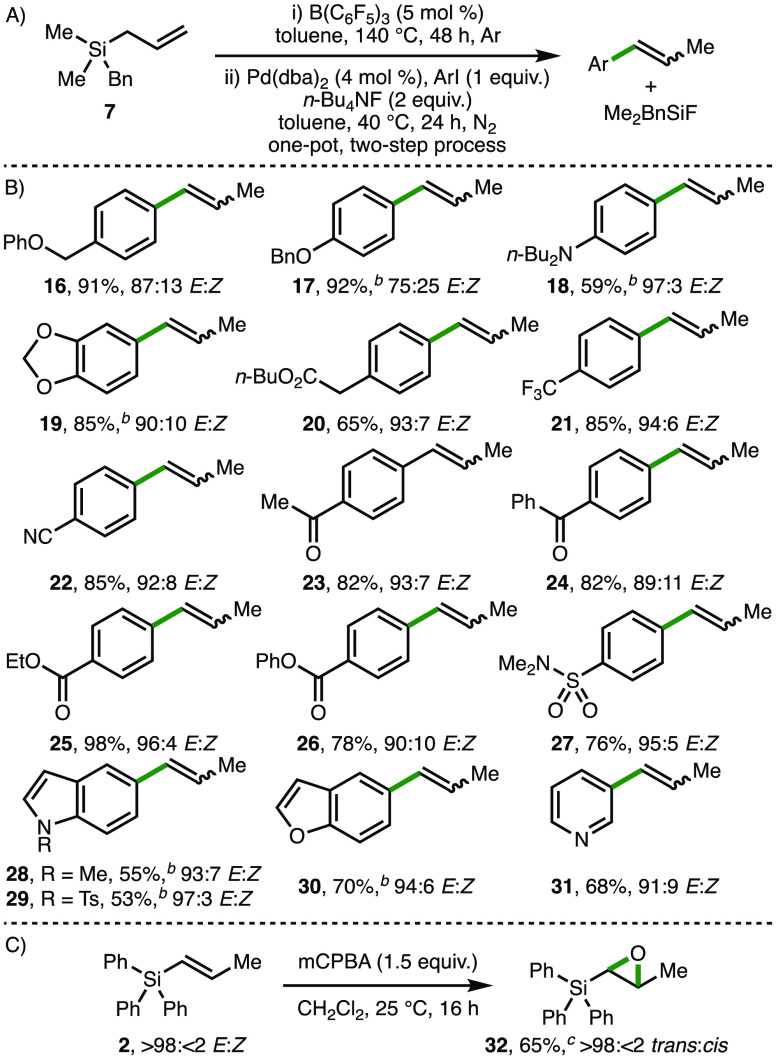
One-Pot Isomerization–Hiyama Coupling Reactions performed using 0.1 mmol of **7**. Yields determined by ^1^H NMR analysis of the crude reaction mixture using 1,3,5-trimethylbenzene as the internal standard. At 50 °C. Isolated yield.

Our investigation of the B(C_6_F_5_)_3_-catalyzed isomerization of allyl benzenes involved a detailed synthetic and computational mechanistic investigation,^18^ which revealed that multiple competing reaction mechanisms may be operative, namely, (i) hydride abstraction, (ii) 1,2-hydride shift, and (iii) 1,3-hydride shift. By analogy, it is proposed that the isomerization of allyl silanes may proceed via the same pathways (Scheme 4A). With a view to providing supporting evidence for plausible reaction intermediates, the B(C_6_F_5_)_3_-catalyzed isomerization of allyltriisopropyl silane **33** was performed in the presence of 1,2-dimethylindole **34** (1.2 equiv), which gave C(3)-alkylated indole **35** in 13% NMR yield alongside alkenyl silane **12** (Scheme 4B). This product indicates the presence of a β-silyl cation intermediate, formed via alkene activation by B(C_6_F_5_)_3_ (cf., proposed 1,2-hydride shift mechanism), which in this case is intercepted by nucleophilic indole **34**.

**Scheme 4:**
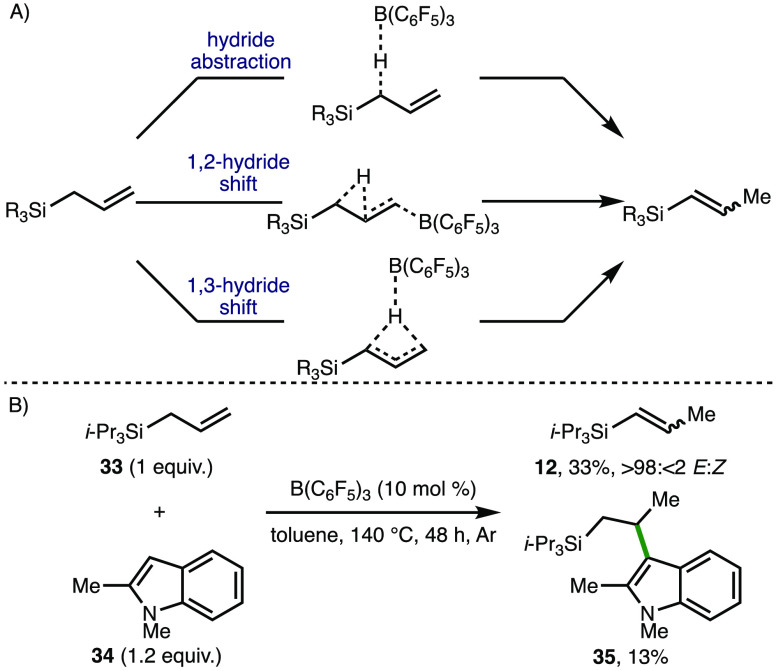
Reaction Mechanism

In conclusion, a one-pot synthesis of styrene derivatives has been developed via a novel B(C_6_F_5_)_3_-catalyzed *E*-selective isomerization of readily accessible allyl silanes and subsequent Hiyama coupling of the versatile alkenyl silane intermediates. This one-pot, two-step approach enables access to a broad range of styrene derivatives, including those containing Lewis basic functional groups that cannot be accessed via the previously developed B(C_6_F_5_)_3_-catalyzed isomerization of allyl benzenes. Ongoing work in our laboratory is focused on further applications of Lewis acid triarylborane catalysts in organic synthesis.

## Notes

The data underlying this study are openly available in the Cardiff University data catalogue at 10.17035/d.2022.0232368584.

## Supporting Information Available

Optimization data, experimental procedures, characterization of new compounds, and spectral data (PDF)
