# Supplementary material for: One-Pot Synthesis of Styrene Derivatives from Allyl Silanes via B(C6F5)3-Catalyzed Isomerization–Hiyama Coupling
Source: Org Lett. 2022 Nov 17;24(47):8694–7. doi: 10.1021/acs.orglett.2c03584 (PMC9724089; doi:10.1021/acs.orglett.2c03584)
Supplement: Supplementary file 1 — ol2c03584_si_001.pdf [file ol2c03584_si_001.pdf]

SUPPORTING INFORMATION

One-Pot Synthesis of Styrene Derivatives from Allyl Silanes via $B(C_6F_5)_3$-Catalyzed Isomerization – Hiyama Coupling

Betty A. Kustiana, Rebecca L. Melen, Louis C. Morrill*

e-mail*: MorrillLC@cardiff.ac.uk

Cardiff Catalysis Institute, School of Chemistry, Cardiff University, Main Building, Park Place, Cardiff, CF10 3AT, U.K.

Contents

1. General information 2
2. Experimental and characterization data 3
2.1. Synthesis of substrates 3
2.1.1. General procedure 1: allylsilane preparation 3
2.1.2. Iodoarenes preparation 20
2.2. Optimizations 32
2.2.1. Optimization of allylsilane isomerization 32
2.2.2. Optimization of one-pot isomerization/Hiyama cross-coupling 33
2.3. Substrate scope 34
2.3.1. General procedure 2 34
2.3.2. Characterization of allylsilane isomerization products 34
2.3.3. General procedure 3 51
2.4. Intermediates trapping 70
3. References 72

1. General information

Unless stated otherwise, reactions were performed using oven-dried 10 mL microwave vials sealed with an aluminium crimp cap and were stirred with Teflon-coated magnetic stirrer bars. Dry toluene was obtained after previously degassed solvent with Schlenk technique through activated alumina columns (Mbraun, SPS-800). All other solvents and commercial reagents were kept with activated molecular sieves and used without further purification unless stated otherwise. All $B(C_6F_5)_3$-catalyzed isomerization reactions were prepared in the glovebox under argon atmosphere.

Room temperature (rt) refers to 20–25 ˚C. All reactions involving heating were conducted using DrySyn blocks and a contact thermometer. *In vacuo* refers to reduced pressure of rotary evaporator. $B(C_6F_5)_3$ was obtained commercially from Acros and purified by sublimation three times before use.

Analytical thin layer chromatography was performed using silica coated aluminium plates (Kieselgel 60 F254 silica) and visualization was obtained using ultraviolet light (254 nm). Flash chromatography used Kieselgel 60 silica with the eluent stated.

Melting points were obtained on a Gallenkamp melting point apparatus and corrected by linear interpolation of melting points standards benzophenone (47–49 ˚C), and benzoic acid (121–123 ˚C).

^{1}H, and ^{13}C NMR spectra were obtained on either a Bruker Avance 300 (300 MHz ^{1}H, 75 MHz ^{13}C) or a Bruker Avance 400 (400 MHz ^{1}H, 101 MHz ^{13}C) or a Bruker Avance 500 (500 MHz ^{1}H, 126 MHz ^{13}C) spectrometer at rt in the solvent stated. Chemical shifts are reported in parts per million (ppm) relative to the residual solvent signal. All coupling constants, *J*, are quoted in Hz. Multiplicities are reported as the following symbols: s = singlet, d = doublet, t = triplet, q = quartet, m = multiplet, qd = quartet of doublet, dqd = doublet of quartet of doublet, ddtd = doublet of doublet of triplet of doublet and multiples thereof.

High and low resolutions mass spectrometry (HR- and LRMS, m/z) data were obtained at Cardiff University on a Micromass LCT spectrometer. The NMR yields and the *E*/*Z* ratio were determined by integration of suitable baseline separated ^{1}H NMR signals. The *E*/*Z* isomers were distinguished by the coupling constant (*J*) of olefinic signals and/or literatures.

2. Experimental and characterization data

2.1. Synthesis of substrates

2.1.1. General procedure 1: allylsilane preparation

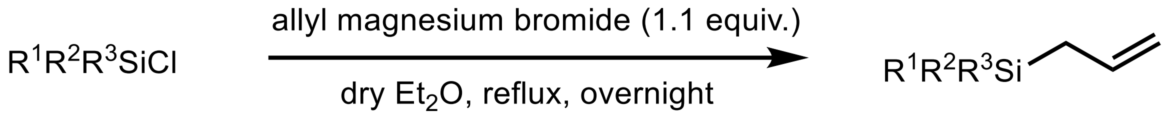

Under nitrogen, a 100 mL three-necked round-bottomed flask with a stirrer bar and a condenser was charged with chlorosilane (3 mmol), and dry Et_2O (12 mL), followed by the dropwise addition of allyl magnesium bromide (3.3 mmol) at 0 ˚C with stirring. The reaction was then refluxed overnight. The reaction was quenched with sat. NH_4Cl (10 mL), the organic phase was separated. The organic phase was washed with brine (2 x 10 mL), dried over $MgSO_4$, filtered, and concentrated *in vacuo*.

Allyltriphenylsilane[1]

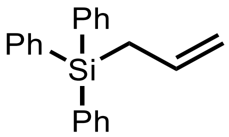

The title compound was prepared according to general procedure 1 using triphenylchlorosilane (3 mmol). Purification by flash silica chromatography (eluent = 100% PE) gave the title compound as white solid (0.72 g, 80%); mp 88–89 ˚C (lit. 88-89 ˚C)[2]; R_f = 0.40 (eluent = 100% PE); **^{1}H NMR (300 MHz, Chloroform-*d*)** δ 7.57 – 7.50 (m, 6H), 7.47 – 7.32 (m, 9H), 5.88 (ddt, *J* = 16.9, 10.1, 7.9 Hz, 1H), 5.04 – 4.83 (m, 2H), 2.41 (dt, *J* = 7.9, 1.3 Hz, 2H); **^{13}C NMR (75 MHz, Chloroform-*d*)** δ 135.9, 134.7, 133.9, 129.7, 128.0, 115.2, 21.3.

^{1}H NMR, 300 MHz, $CDCl_3$

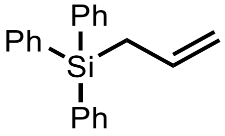

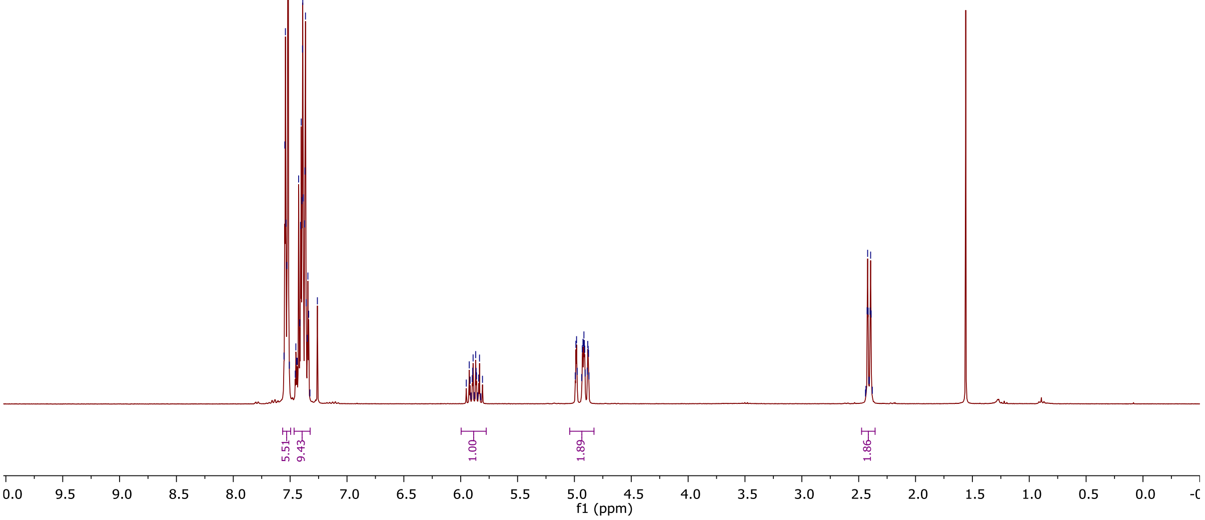

^{13}C NMR, 75 MHz, $CDCl_3$

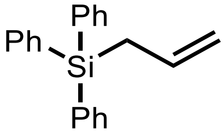

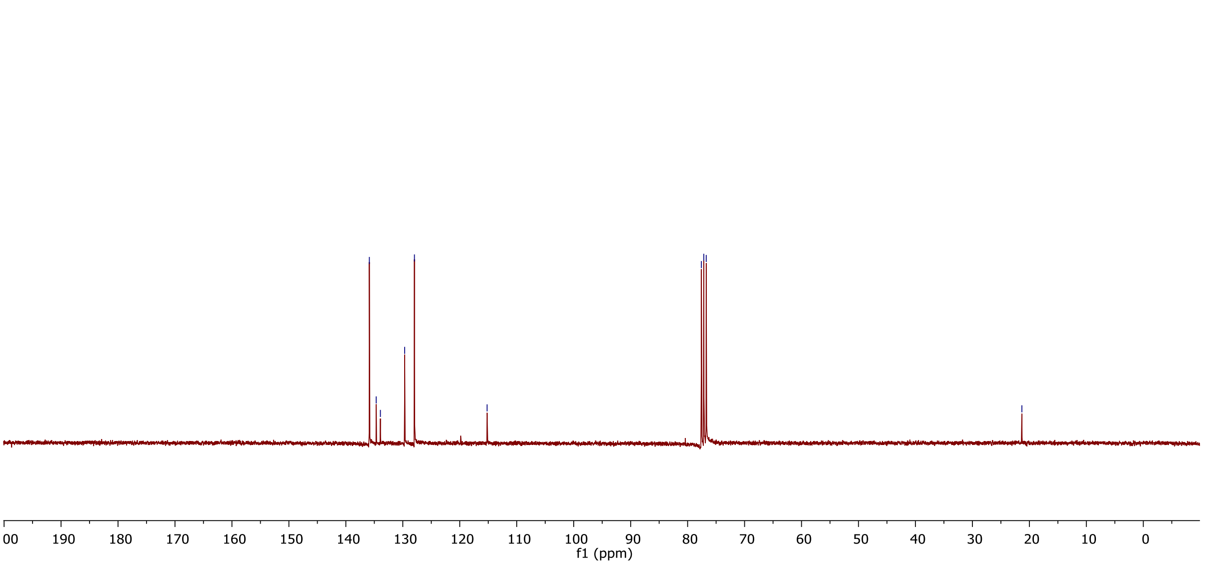

3-(*tert*-butyldiphenylsilyl)propene[1]

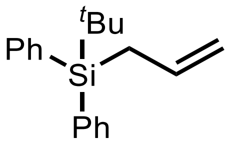

The title compound was prepared according to general procedure 1 using tert-butyldiphenylchlorosilane (5 mmol). Purification by flash silica chromatography (eluent = 100% PE) gave the title compound as colourless liquid (0.70 g, 50%); R_f: 0.35 (eluent = 100% PE); **^{1}H NMR (300 MHz, Chloroform-*d*)** δ 7.67 – 7.58 (m, 4H), 7.45 – 7.32 (m, 6H), 5.78 (ddt, *J* = 16.9, 10.0, 7.9 Hz, 1H), 4.98 – 4.74 (m, 2H), 2.20 (dt, *J* = 7.9, 1.4 Hz, 2H), 1.07 (s, 9H); **^{13}C NMR (126 MHz, Chloroform-*d*)** δ 136.1, 134.8, 134.5, 129.2, 127.7, 114.6, 28.0, 18.9, 18.6.

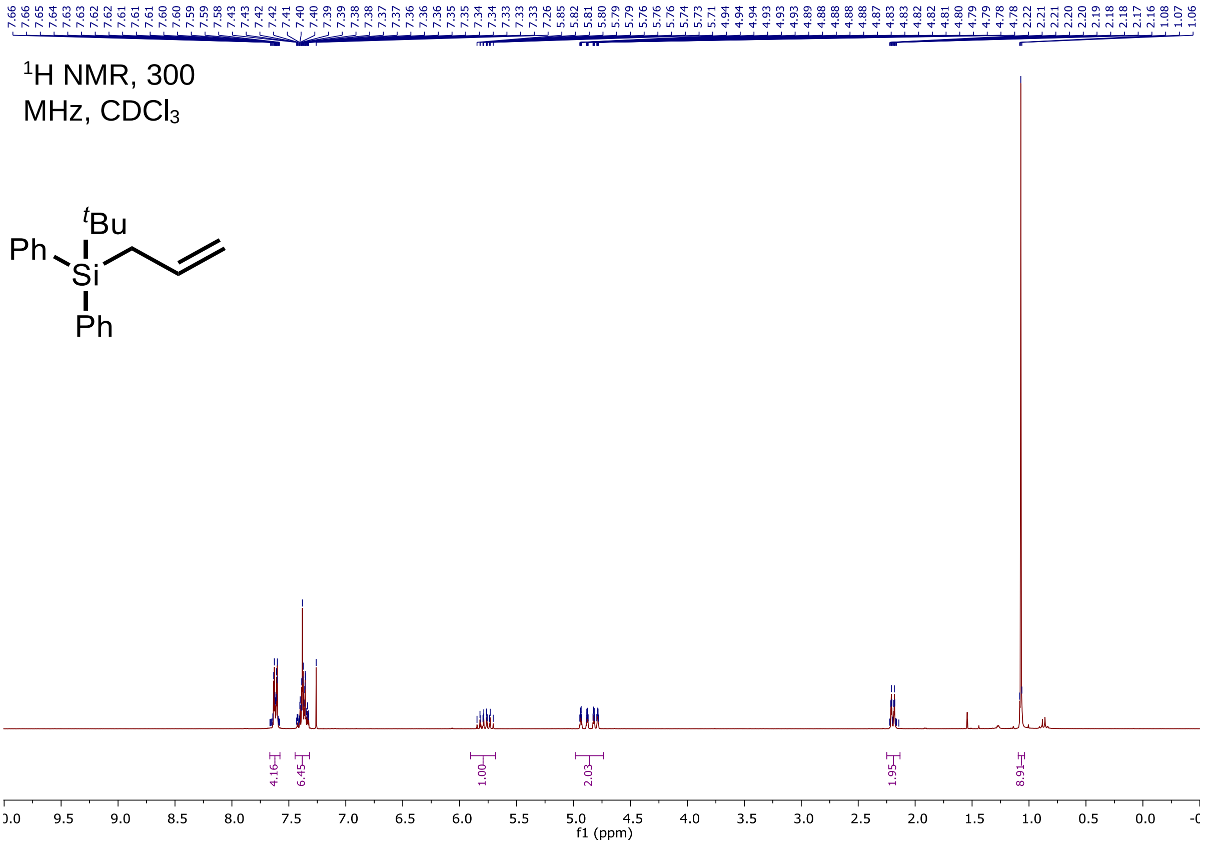

^{13}C NMR, 75 MHz, $CDCl_3$

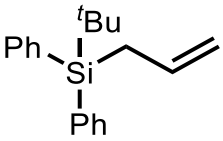

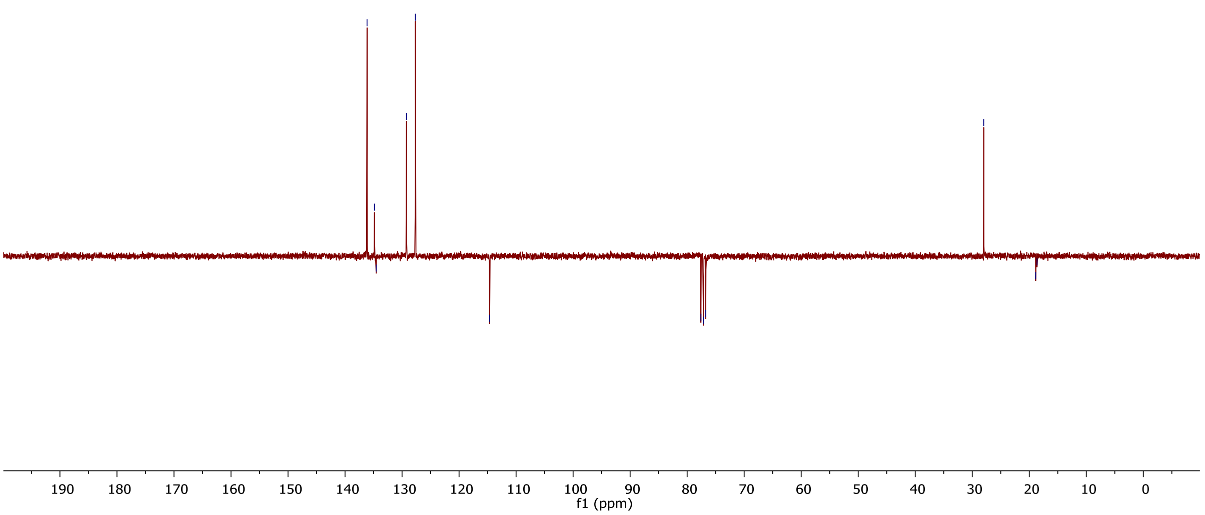

Allyldiphenyl(methyl)silane[1]

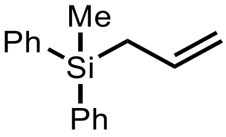

The title compound was prepared according to general procedure 1 using diphenyl(methyl)chlorosilane (3 mmol). Purification by flash silica chromatography (eluent = 5% EtOAc in PE) gave the title compound as colourless liquid (0.44 g, 61%); R_f: 0.65 (eluent = 5% EtOAc in PE); **^{1}H NMR (300 MHz, Chloroform-*d*)** δ 7.58 – 7.49 (m, 4H), 7.44 – 7.30 (m, 6H), 5.81 (ddt, *J* = 17.1, 10.1, 8.0 Hz, 1H), 5.01 – 4.80 (m, 2H), 2.09 (dt, *J* = 8.0, 1.2 Hz, 2H), 0.57 (s, 3H); **^{13}C NMR (75 MHz, Chloroform-*d*)** δ 136.7, 134.7, 134.2, 129.4, 127.9, 114.4, 22.3, -4.7.

^{1}H NMR, 300 MHz, $CDCl_3$

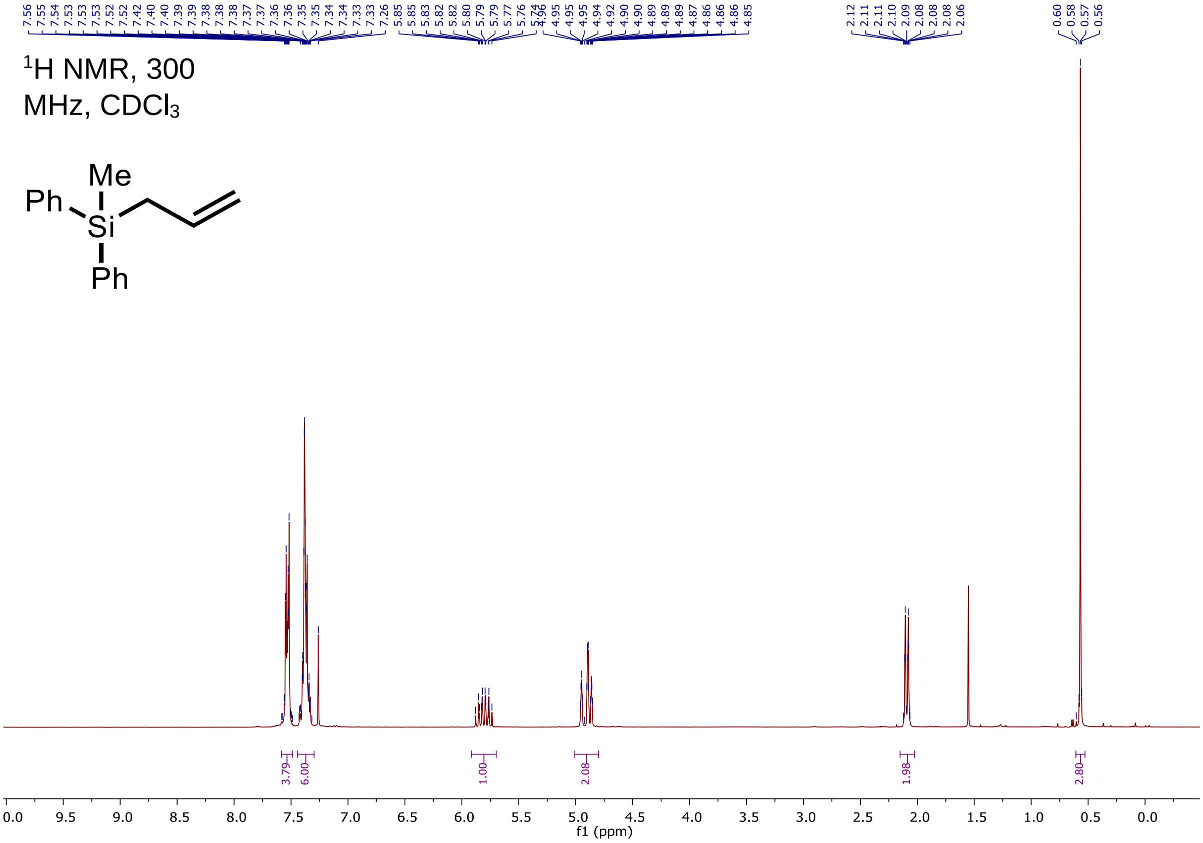

^{13}C NMR, 75 MHz, $CDCl_3$

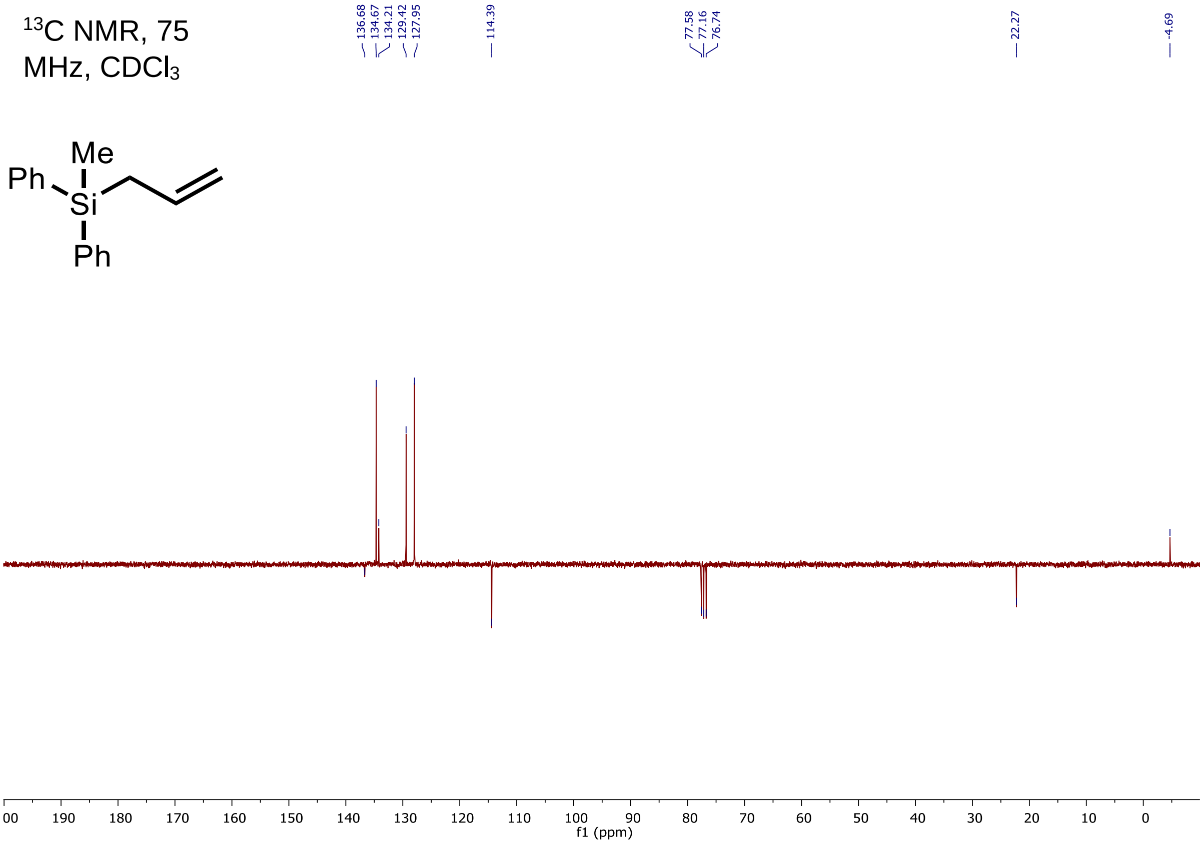

Allylhexylmethylphenylsilane

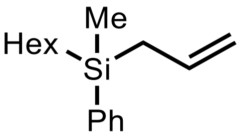

The title compound was prepared according to general procedure 1 using hexylmethylphenylchlorosilane (3 mmol). Purification by flash silica chromatography (eluent = 100% PE) gave the title compound as colourless liquid (0.46 g, 62%); R_f: 0.80 (eluent = 100% PE); **^{1}H NMR (500 MHz, Chloroform-*d*)** δ 7.52 – 7.48 (m, 2H), 7.37 – 7.33 (m, 3H), 5.77 (ddt, *J* = 17.0, 10.1, 8.1 Hz, 1H), 4.91 – 4.80 (m, 2H), 1.78 (dt, *J* = 8.1, 1.2 Hz, 2H), 1.33 – 1.21 (m, 8H), 0.89 – 0.84 (m, 3H), 0.82 – 0.77 (m, 2H), 0.27 (s, 3H).; **^{13}C NMR (126 MHz, Chloroform-*d*)** δ 138.1, 134.9, 134.0, 129.1, 127.8, 113.5, 33.4, 31.6, 23.8, 22.7, 22.4, 14.3, 13.8, -5.3; **HRMS** (CI) calculated $[C_{13}H_{21}Si]^{+}$ $(M-C_3H_5)^{+}$: m/z 205.1407, found 205.1406; **IR** (film, ν_{max} / cm^{-1}) 2955, 2920, 2855, 1630, 1427, 1250, 1111, 893, 797.

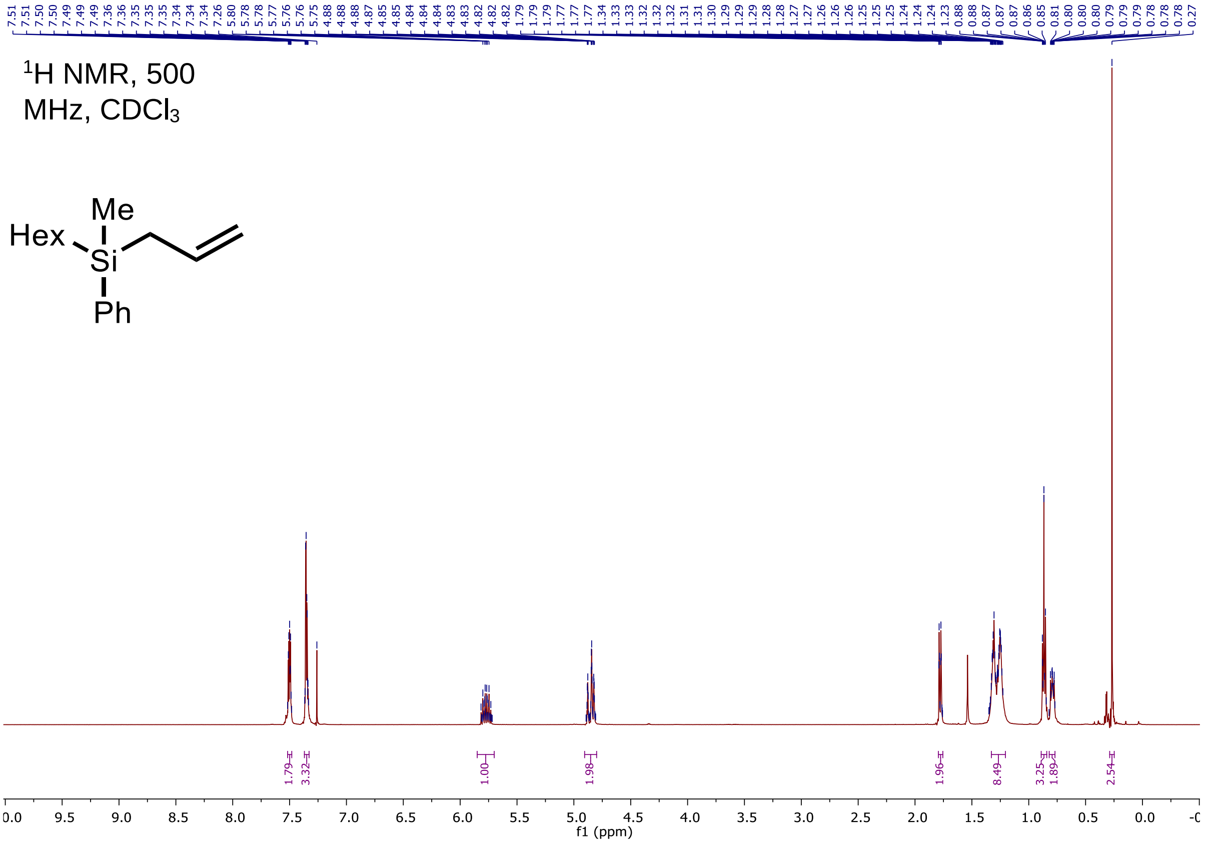

^{13}C NMR, 126 MHz, $CDCl_3$

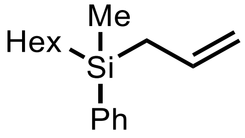

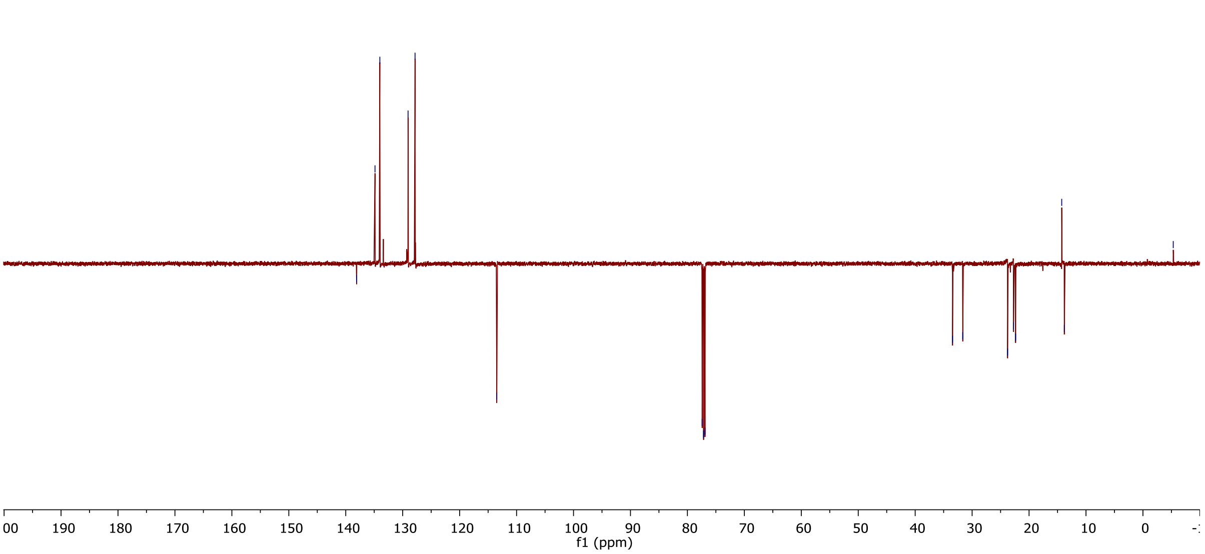

Allyl(phenyl)dimethylsilane[1]

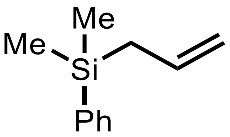

The title compound was prepared according to general procedure 1 using dimethylphenylchlorosilane (3 mmol). Purification by flash silica chromatography (eluent = 100% PE) gave the title compound as colourless liquid (0.26 g, 50%); R_f: 0.55 (eluent = 100% PE); **^{1}H NMR (500 MHz, Chloroform-*d*)** δ 7.55 – 7.48 (m, 2H), 7.40 – 7.32 (m, 3H), 5.78 (ddtd, *J* = 16.9, 10.2, 8.1, 0.7 Hz, 1H), 4.92 – 4.79 (m, 2H), 1.76 (ddt, *J* = 8.1, 1.6, 0.8 Hz, 2H), 0.28 (d, *J* = 0.7 Hz, 6H); **^{13}C NMR (126 MHz, Chloroform-*d*)** δ 138.8, 134.8, 133.8, 129.1, 127.9, 113.5, 23.8, -3.3.

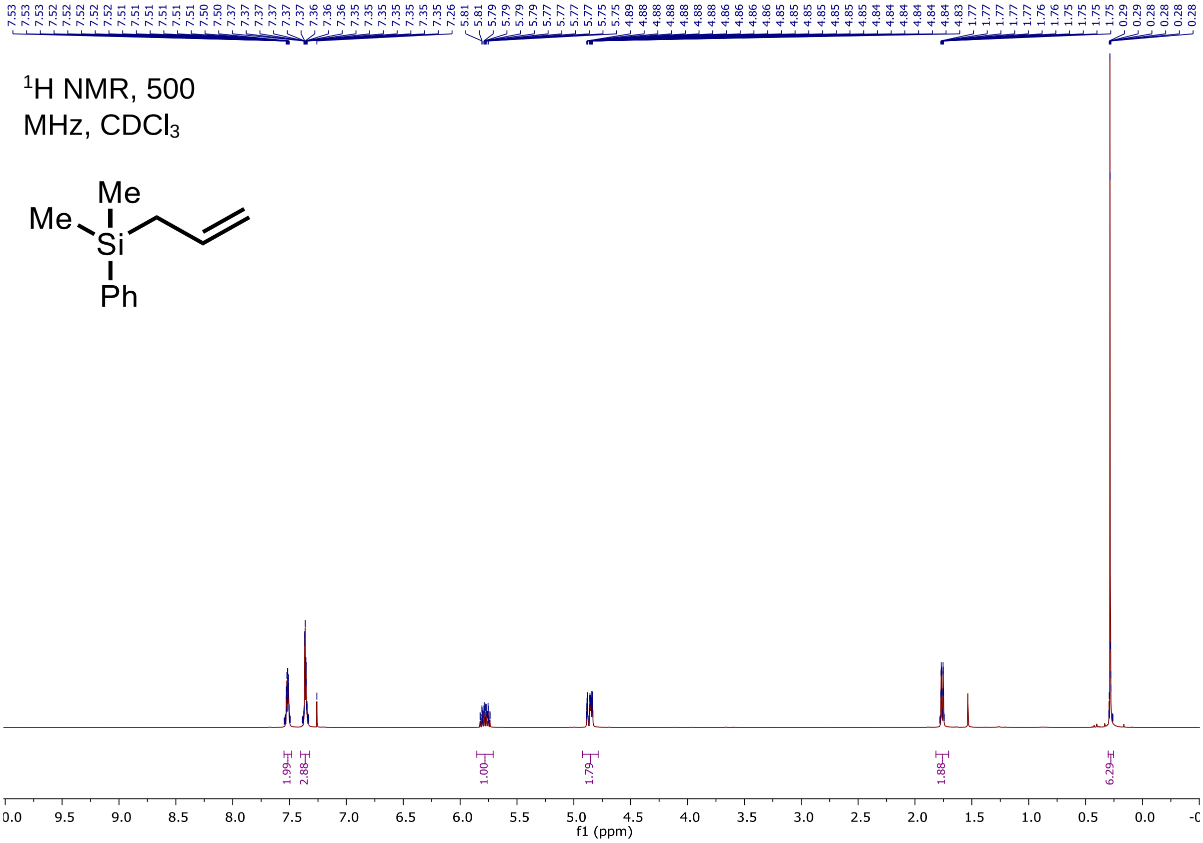

1H NMR, 500 MHz, CDCl3
Me
Me
Si
Ph
1.99
2.88
1.00
1.79
1.88
6.29
f1 (ppm)

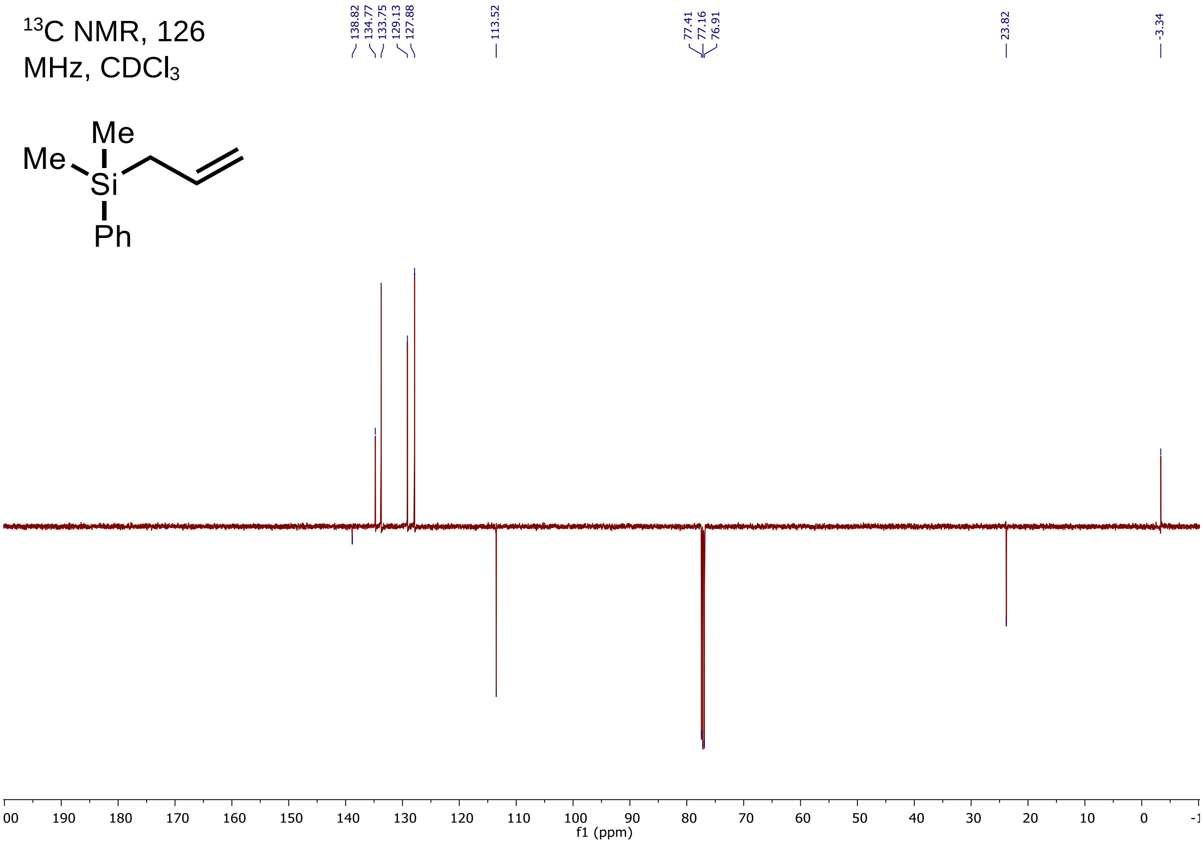

13C NMR, 126 MHz, CDCl3
138.82
134.77
133.75
129.13
127.88
113.52
77.41
77.16
76.91
23.82
-3.34
Me
Me
Si
Ph
f1 (ppm)

Allyl(benzyl)dimethylsilane[3]

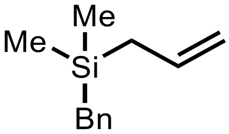

The title compound was prepared according to general procedure 1 using benzyldimethylchlorosilane (3 mmol). Purification by flash silica chromatography (eluent = 100% PE) gave the title compound as colourless liquid (0.28 g, 50%); R_f: 0.64 (eluent = 100% PE); **^{1}H NMR (300 MHz, Chloroform-*d*)** δ 7.25 – 7.18 (m, 2H), 7.11 – 7.04 (m, 1H), 7.03 – 6.99 (m, 2H), 5.86 – 5.68 (m, 1H), 4.93 – 4.81 (m, 2H), 2.11 (s, 2H), 1.55 – 1.50 (m, 2H), -0.02 (s, 6H); **^{13}C NMR (75 MHz, Chloroform-*d*)** δ 140.1, 134.9, 128.33, 128.27, 124.1, 113.4, 25.2, 22.8, -3.9.

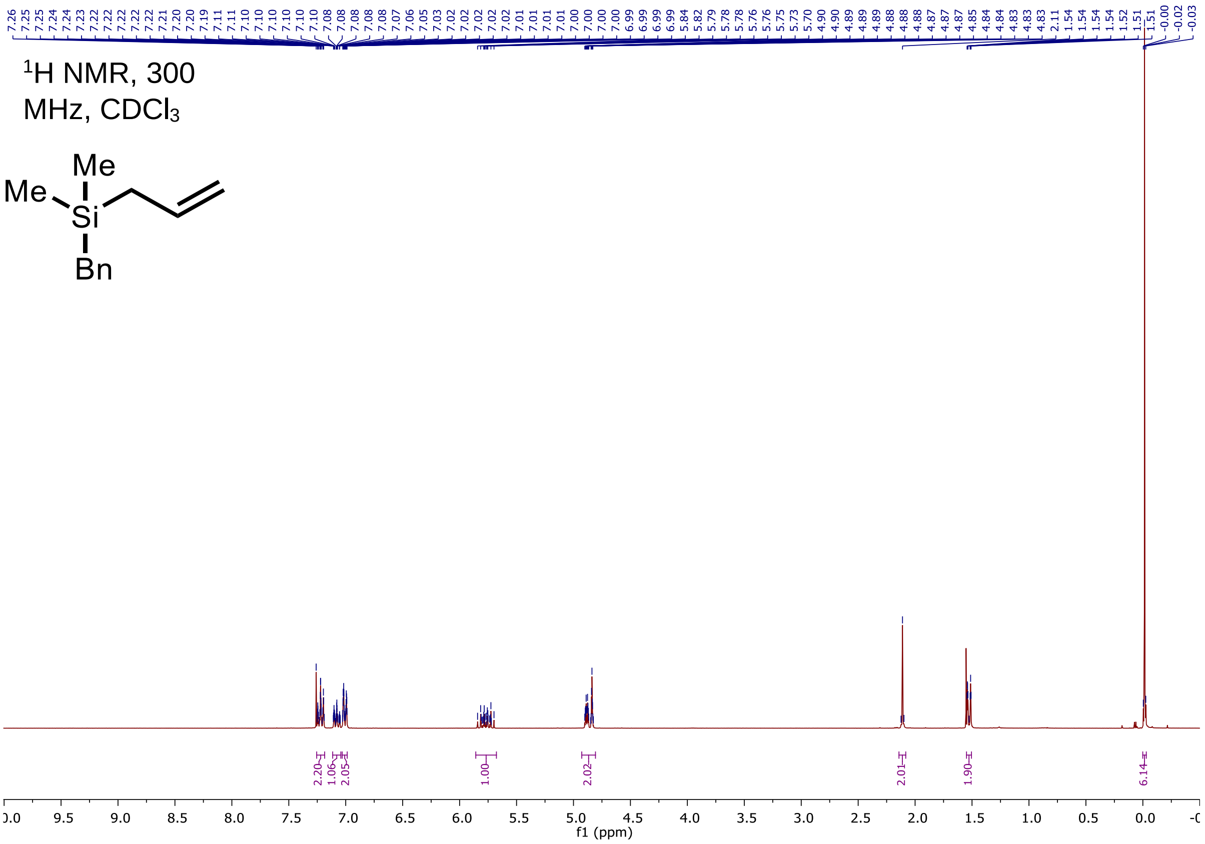

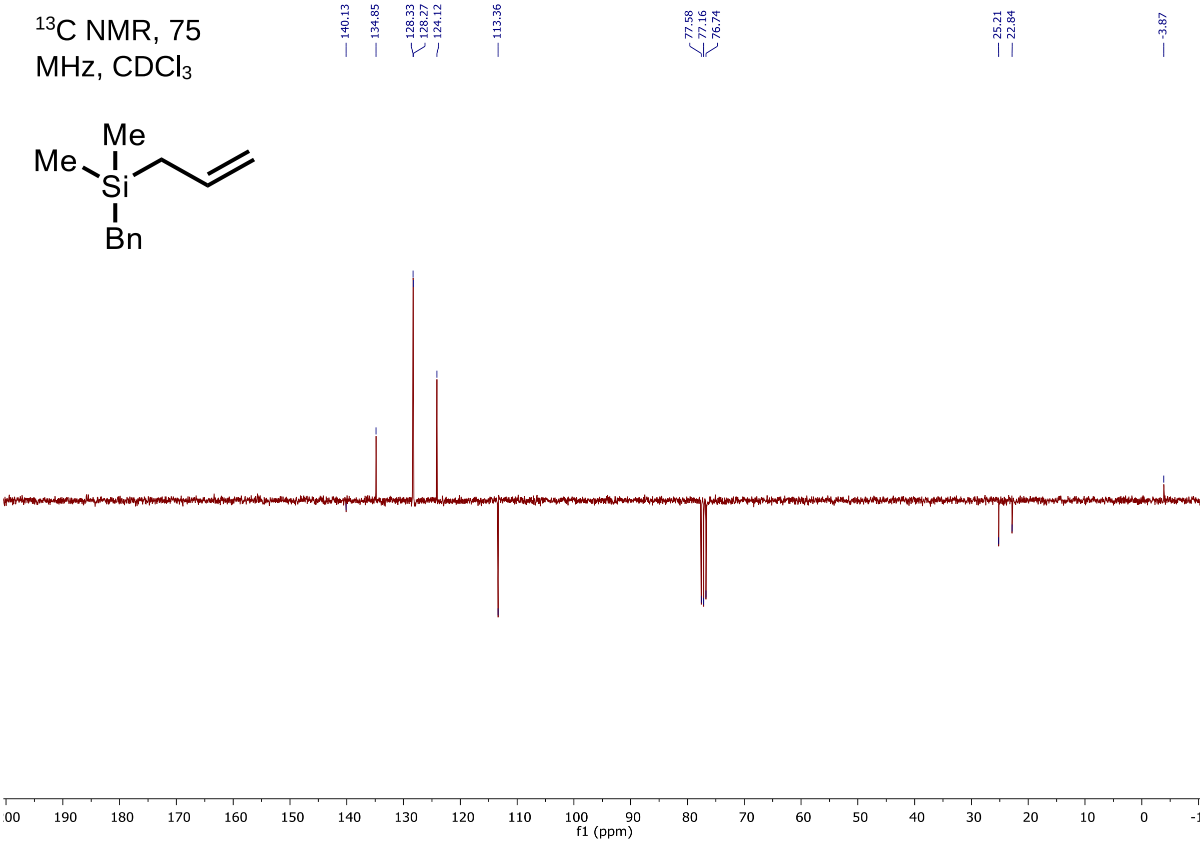

Allyl(3-phenylpropyl)dimethylsilane

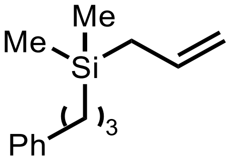

The title compound was prepared according to general procedure 1 using (3-phenylpropyl)dimethylchlorosilane (3 mmol). Purification by flash silica chromatography (eluent = 100% PE) gave the title compound as colourless liquid (0.45 g, 69%); R_f: 0.53 (eluent = 100% PE); **^{1}H NMR (500 MHz, Chloroform-*d*)** δ 7.33 – 7.28 (m, 2H), 7.23 – 7.18 (m, 3H), 5.79 (ddt, *J* = 16.9, 10.2, 8.1 Hz, 1H), 4.88 – 4.81 (m, 2H), 2.67 – 2.62 (m, 2H), 1.70 – 1.61 (m, 2H), 1.56 – 1.51 (m, 2H), 0.63 – 0.58 (m, 2H), 0.01 (s, 6H); **^{13}C NMR (126 MHz, Chloroform-*d*)** δ 142.8, 135.3, 128.6, 128.4, 125.8, 112.8, 40.1, 26.1, 23.4, 14.9, -3.6; **HRMS** (CI) calculated $[C_{11}H_{17}Si]^+$ $(M-C_3H_5)^+$: m/z 177.1094, found 177.1092; **IR** (film, ν_{max} / cm^{-1}) 3026, 2953, 2920, 1630, 1497, 1248, 1153, 831, 743.

^{1}H NMR, 500 MHz, $CDCl_3$

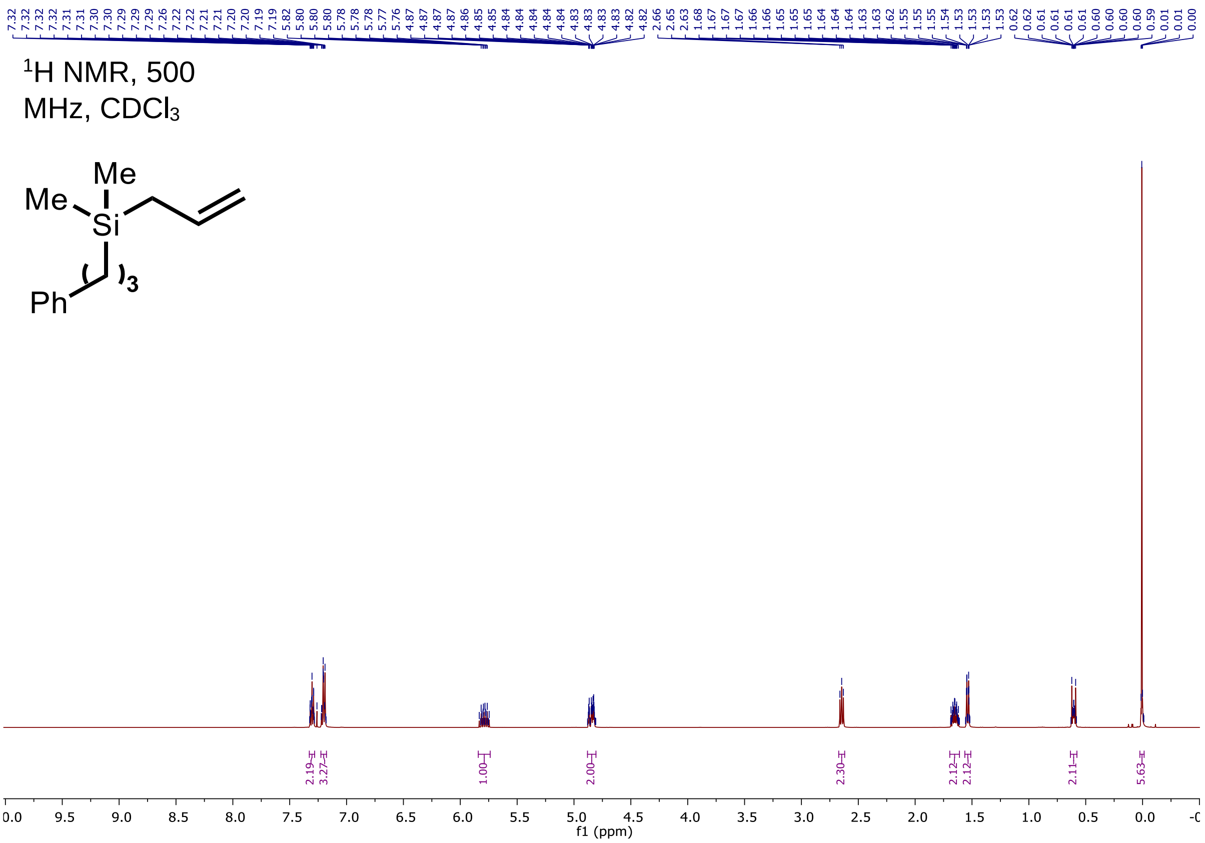

^{13}C NMR, 126 MHz, $CDCl_3$

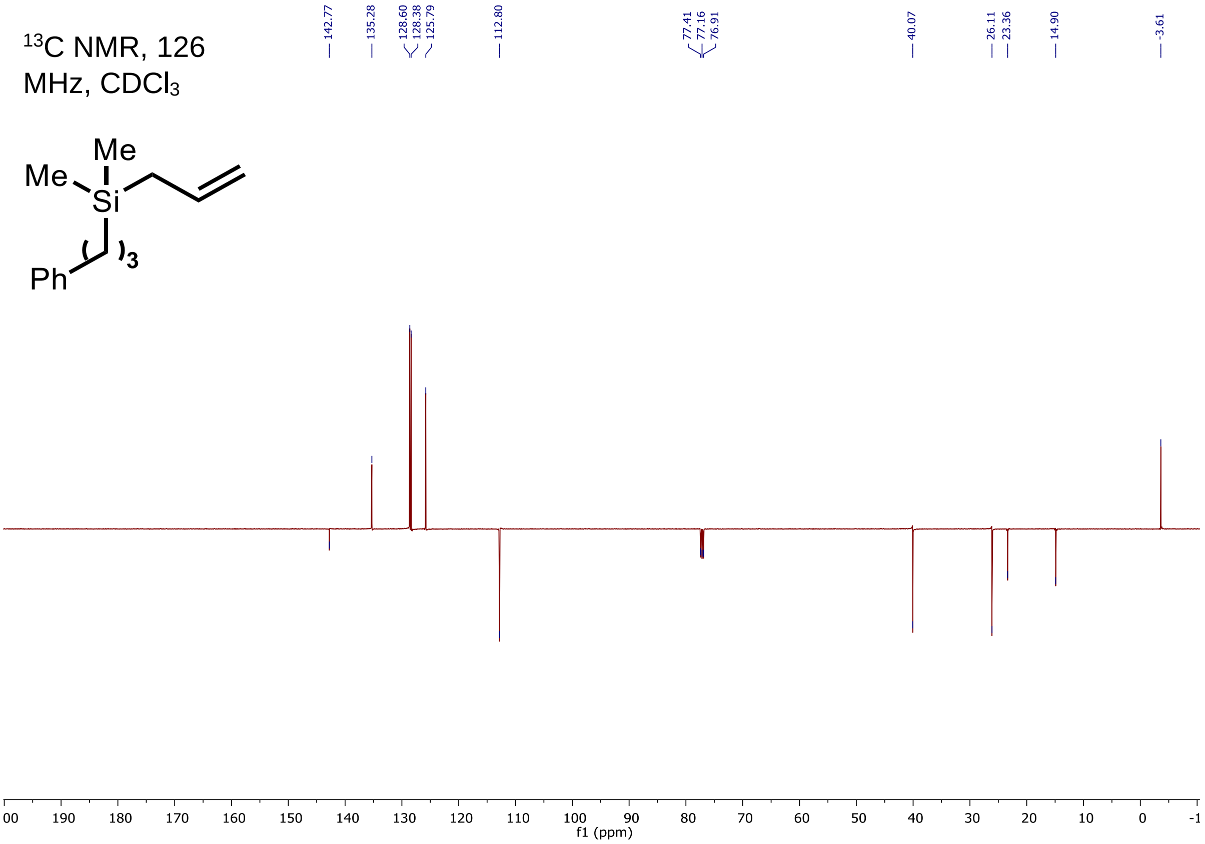

Allyl(decyl)dimethylsilane

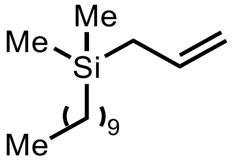

The title compound was prepared according to general procedure 1 using (decyl)dimethylchlorosilane (3 mmol). Purification by flash silica chromatography (eluent = 100% PE) gave the title compound as colourless liquid (0.68 g, 95%); R_f: 0.79 (eluent = 100% PE); **^{1}H NMR (400 MHz, Chloroform-*d*)** δ 5.78 (ddt, *J* = 16.9, 10.1, 8.1 Hz, 1H), 4.88 – 4.77 (m, 2H), 1.51 (dt, *J* = 8.1, 1.2 Hz, 2H), 1.26 (s, 15H), 0.89 (d, *J* = 6.6 Hz, 3H), 0.51 (s, 2H), -0.03 (s, 6H); **^{13}C NMR (101 MHz, Chloroform-*d*)** δ 135.5, 112.6, 33.8, 33.6, 32.1, 29.84, 29.77, 29.5, 23.9, 23.5, 22.9, 18.6, 15.0, 13.8, -3.6; **HRMS** (AP) calculated $[C_{15}H_{32}Si]^+$ $(M)^+$: m/z 240.2273, found 240.2273; **IR** (film, v_{max} / cm^{-1}) 2955, 2920, 2853, 1630, 1250, 1153, 1053, 891, 797.

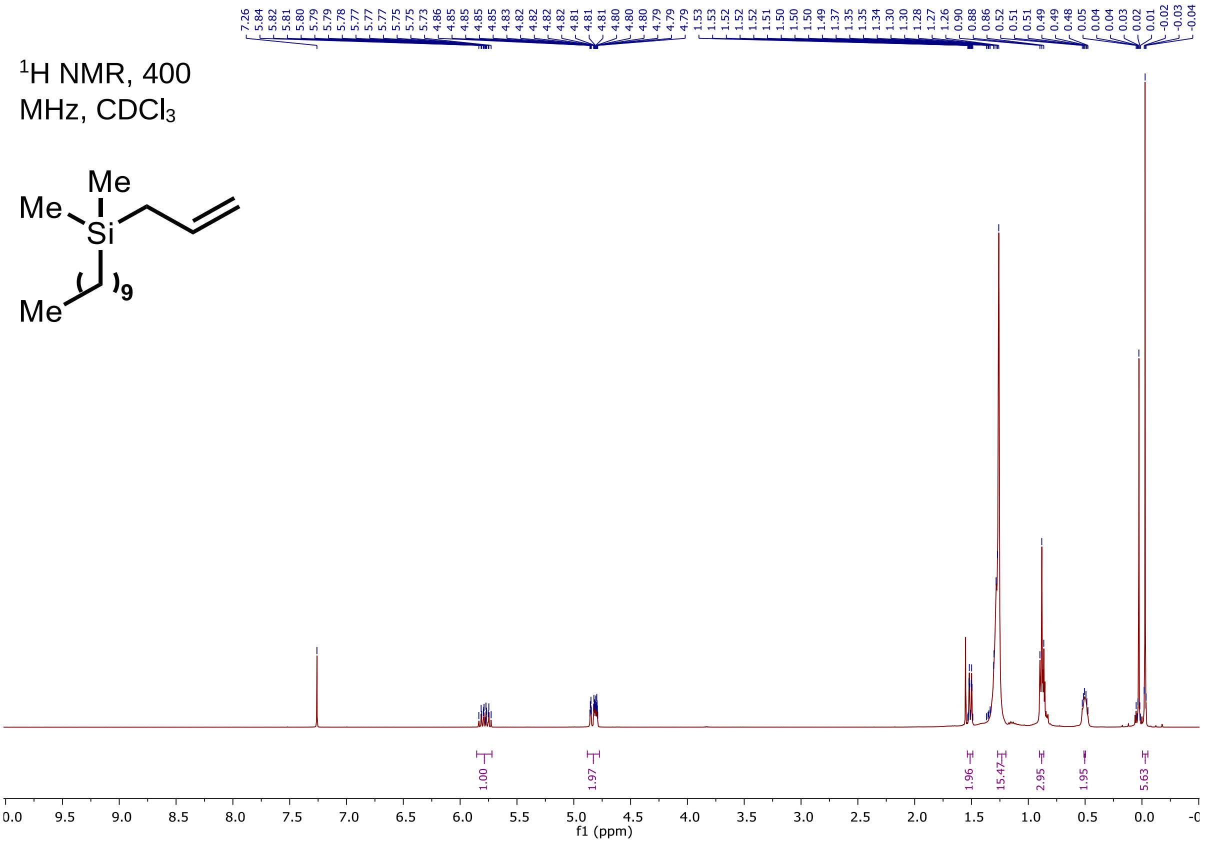

^{13}C NMR, 101 MHz, $CDCl_3$

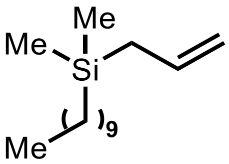

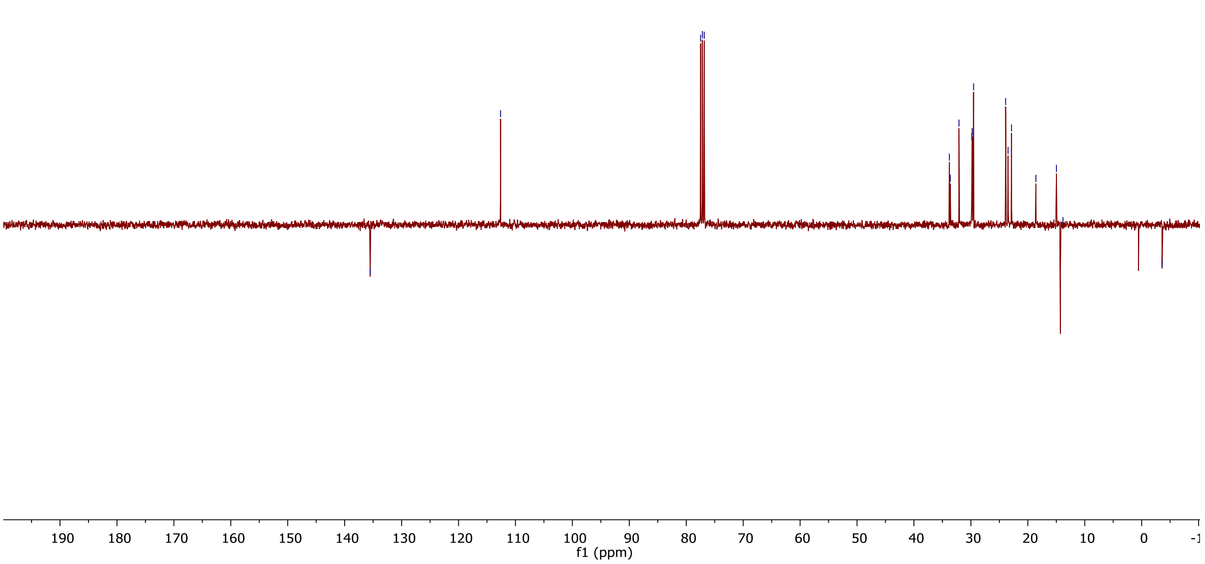

Allyltributylsilane[4]

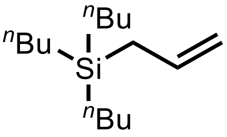

The title compound was prepared according to general procedure 1 using tributylchlorosilane (3 mmol). Purification by flash silica chromatography (eluent = 100% PE) gave the title compound as colourless liquid (0.48 g, 67%); R_f: 0.85 (eluent = 100% PE); **^{1}H NMR (300 MHz, Chloroform-*d*)** δ 5.79 (ddt, *J* = 16.9, 10.1, 8.1 Hz, 1H), 4.91 – 4.76 (m, 2H), 1.53 (ddd, *J* = 8.2, 1.5, 1.1 Hz, 2H), 1.39 – 1.20 (m, 12H), 0.93 – 0.85 (m, 9H), 0.58 – 0.47 (m, 6H); **^{13}C NMR (75 MHz, Chloroform-*d*)** δ 135.7, 112.6, 26.9, 26.2, 20.7, 14.0, 12.0.

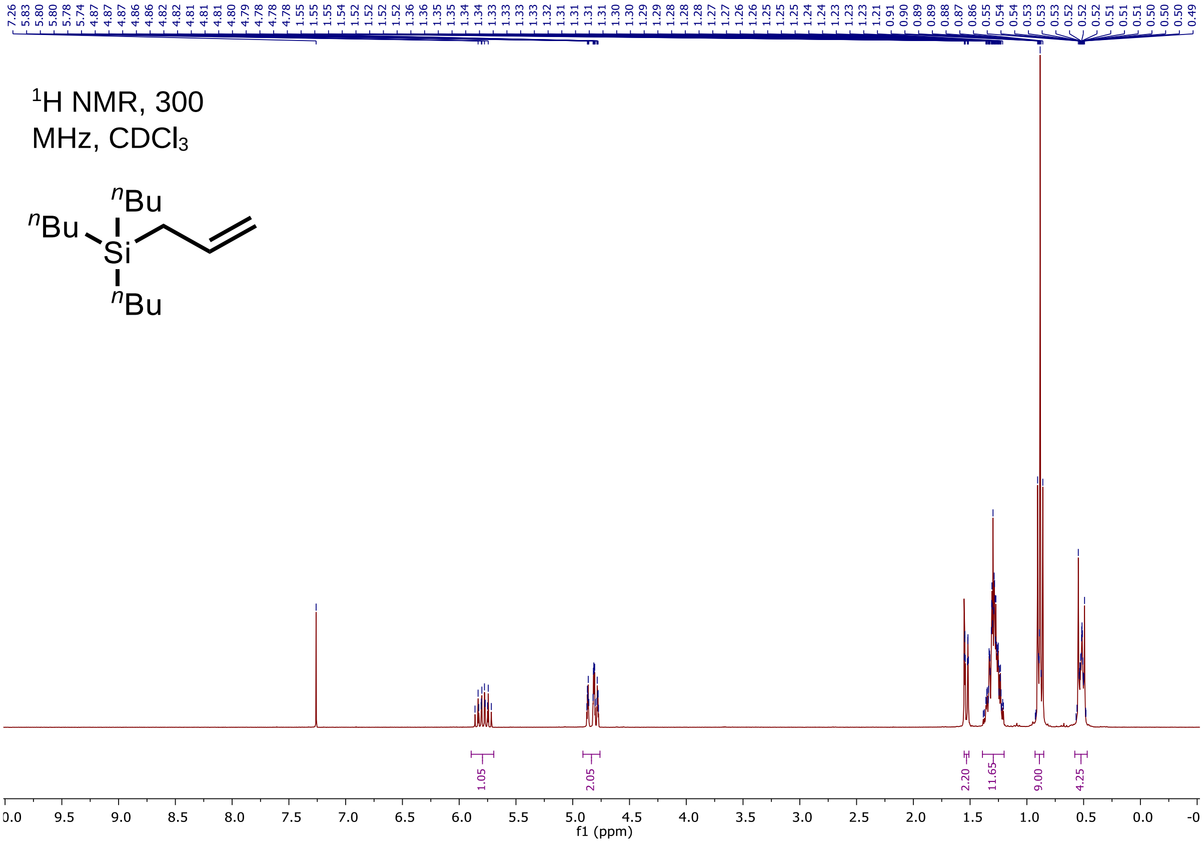
7.26 5.83 5.80 5.78 5.74 4.87 4.87 4.87 4.86 4.82 4.81 4.81 4.80 4.79 4.78 4.78 1.55 1.55 1.55 1.54 1.52 1.52 1.52 1.36 1.36 1.35 1.35 1.34 1.34 1.33 1.33 1.33 1.33 1.32 1.31 1.31 1.31 1.30 1.29 1.29 1.28 1.28 1.28 1.27 1.27 1.26 1.26 1.25 1.25 1.25 1.24 1.24 1.23 1.23 1.23 1.21 0.91 0.90 0.89 0.88 0.87 0.86 0.55 0.54 0.54 0.53 0.53 0.53 0.52 0.52 0.52 0.51 0.51 0.51 0.50 0.50 0.49
1H NMR, 300 MHz, CDCl3
nBu
nBu
Si
nBu
1.05
2.05
2.20
11.65
9.00
4.25
f1 (ppm)

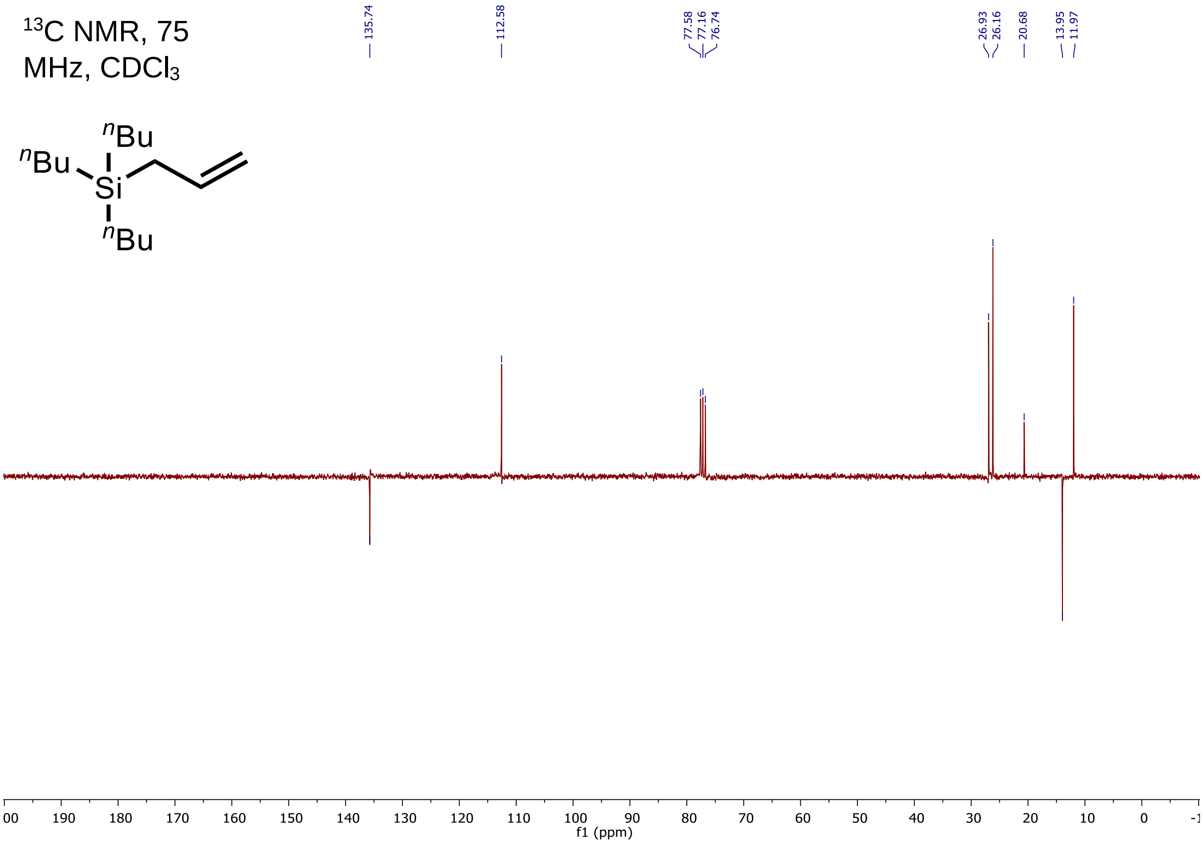
13C NMR, 75 MHz, CDCl3
135.74
112.58
77.58
77.16
76.74
26.93
26.16
20.68
13.95
11.97
nBu
nBu
Si
nBu
f1 (ppm)

Allyltriphenylgermane[5]

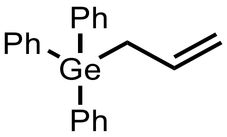

The title compound was prepared according to general procedure 1 using triphenylchlorogermane (3 mmol). Purification by flash silica chromatography (eluent = 100% PE) gave the title compound as white solid (0.62 g, 60%); mp 91–92 ˚C (lit. 90–91 ˚C)[5]; R_f: 0.47 (eluent = 100% PE); **^{1}H NMR (400 MHz, Chloroform-*d*)** δ 7.51 – 7.46 (m, 6H), 7.40 – 7.33 (m, 9H), 5.92 (ddt, *J* = 17.1, 10.1, 8.1 Hz, 1H), 5.04 – 4.80 (m, 2H), 2.49 (dt, *J* = 8.1, 1.2 Hz, 2H); **^{13}C NMR (101 MHz, Chloroform-d)** δ 136.7, 135.1, 134.7, 129.1, 128.3, 114.6, 21.4.

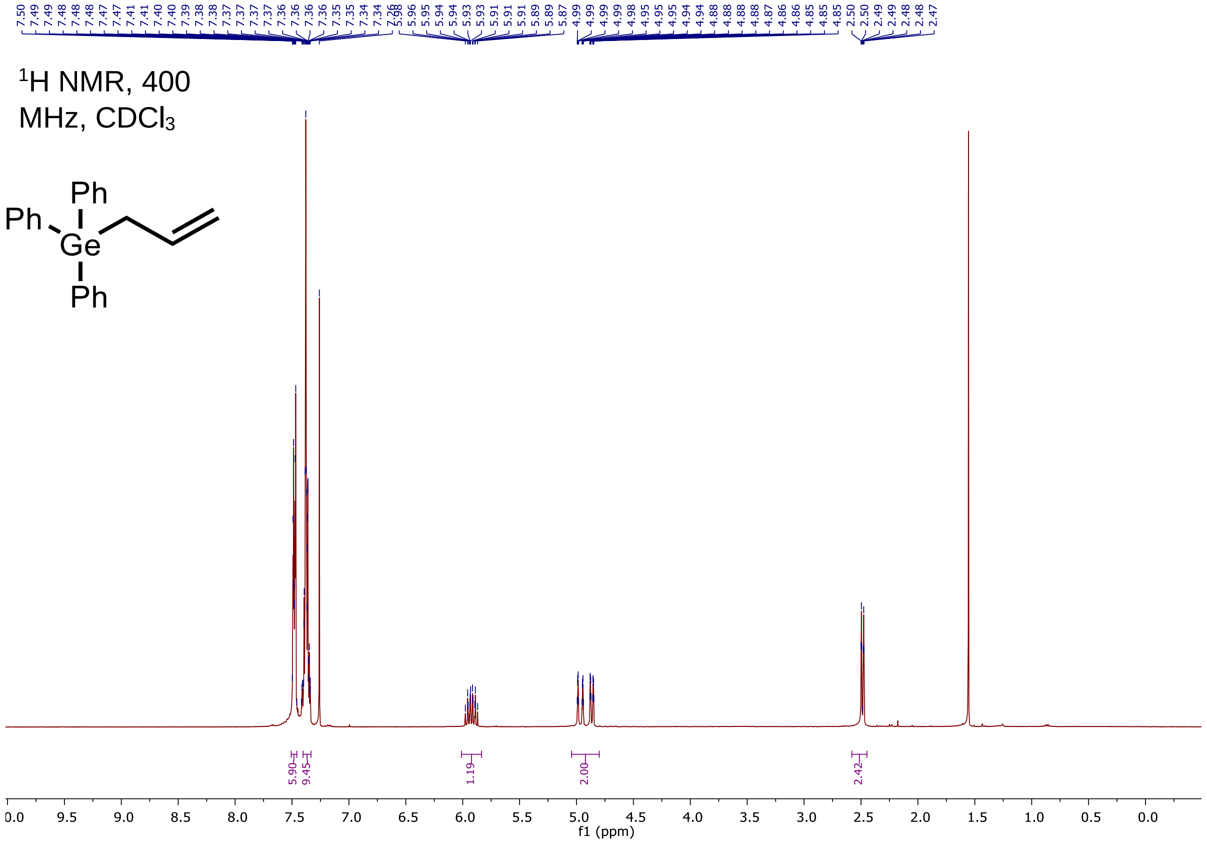

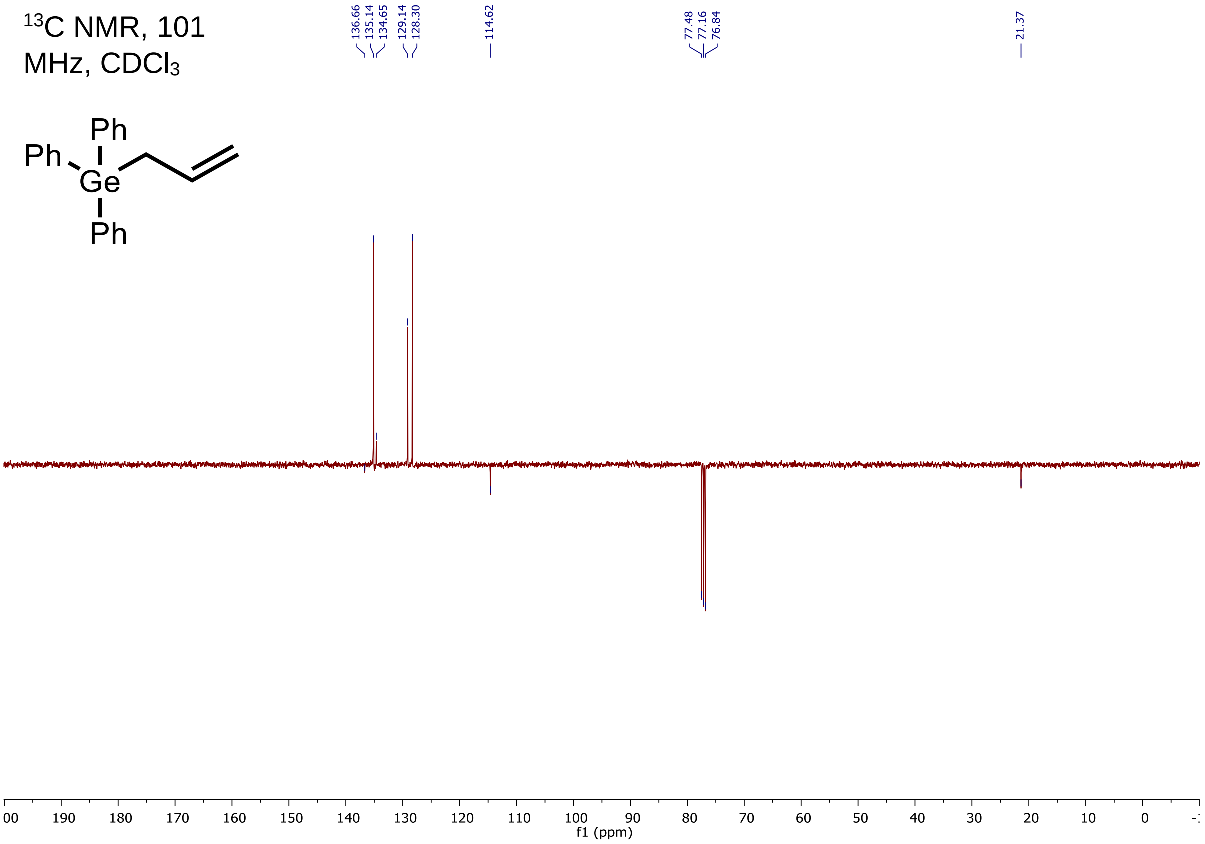

Allyltriethylgermane[6]

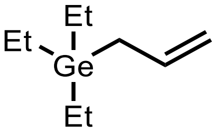

The title compound was prepared according to general procedure 1 using triethylchlorogermane (3 mmol). Purification by flash silica chromatography (eluent = 100% PE) gave the title compound as white solid (0.30 g, 50%); R_f: 0.85 (eluent = 100% PE, $KMnO_4$ stain); **^{1}H NMR (500 MHz, Chloroform-*d*)** δ 5.85 (ddt, *J* = 16.8, 10.0, 8.3 Hz, 1H), 4.91 – 4.72 (m, 2H), 1.68 (ddd, *J* = 8.4, 1.4, 0.9 Hz, 2H), 1.03 (td, *J* = 8.0, 0.5 Hz, 9H), 0.75 (dd, *J* = 7.9, 0.6 Hz, 6H); **^{13}C NMR (126 MHz, Chloroform-d)** δ 136.7, 111.7, 19.0, 9.0, 4.0.

^{1}H NMR, 500 MHz, $CDCl_3$

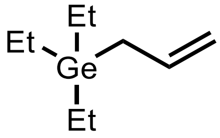

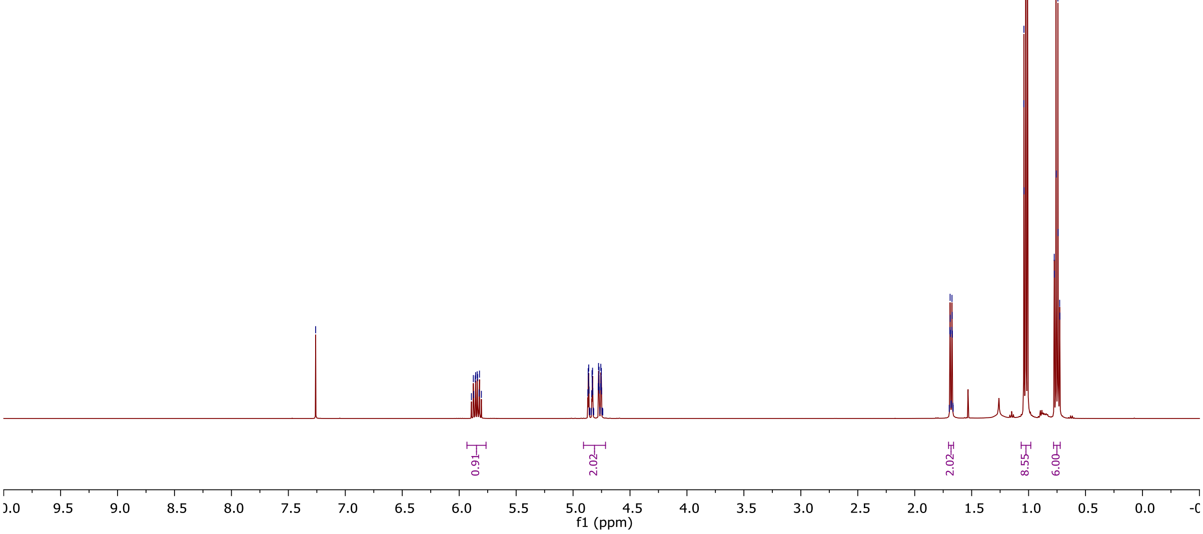

^{13}C NMR, 126 MHz, $CDCl_3$

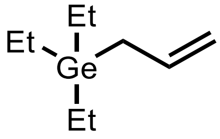

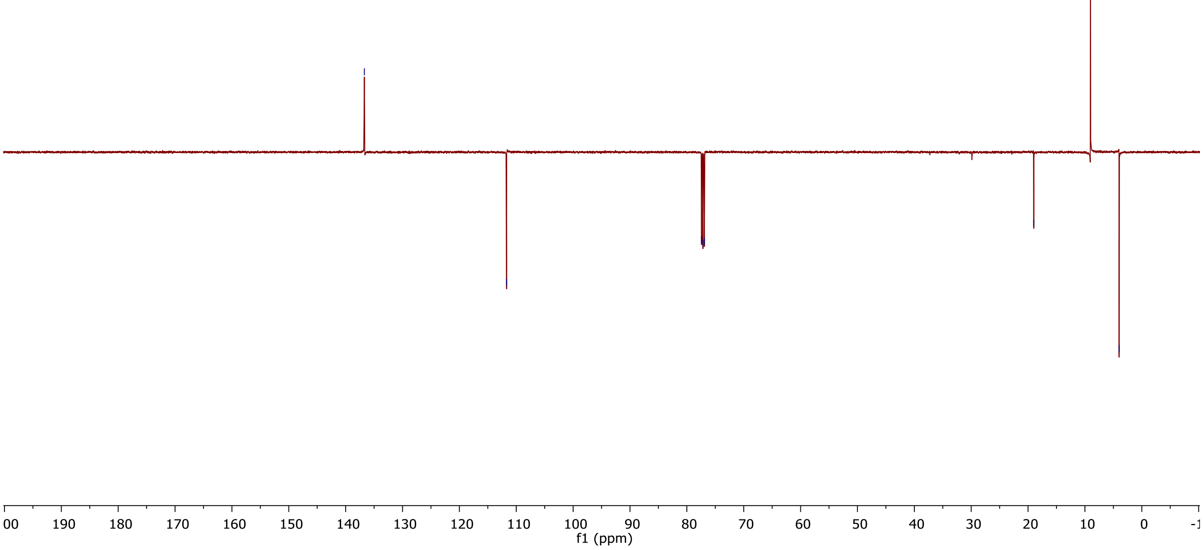

2.1.2. Iodoarenes preparation

***N*,*N*-dibutyl-4-iodoaniline**[7]

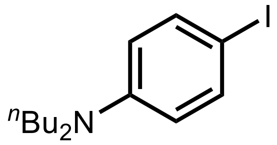

4-iodoaniline (5 mmol, 1.09 g), K_2CO_3 (20 mmol, 2.76 g), and dry DMF (15 mL) were added into 100 mL round-bottomed flask. 1-bromobutane (30 mmol, 3.2 mL) was added to the mixture then stirred at 70 ˚C for 3 days. The mixture was cooled to rt then water (20 mL) was added. The mixture was extracted with EtOAc (1 x 10 mL), washed with brine (3 x 10 mL), dried over $MgSO_4$, filtered, and concentrated *in vacuo*. Purification by flask silica chromatography (eluent = 100% PE) gave the title compound as yellow liquid (0.99 g, 60%); Rf: 0.35 (eluent = 100 PE); **^{1}H NMR (300 MHz, Chloroform-*d*)** δ 7.45 – 7.38 (m, 2H), 6.45 – 6.38 (m, 2H), 3.27 – 3.18 (m, 4H), 1.63 – 1.47 (m, 4H), 1.42 – 1.26 (m, 4H), 1.00 – 0.91 (m, 6H); **^{13}C NMR (75 MHz, Chloroform-*d*)** δ 147.8, 137.8, 114.2, 75.5, 50.9, 29.4, 20.5, 14.1.

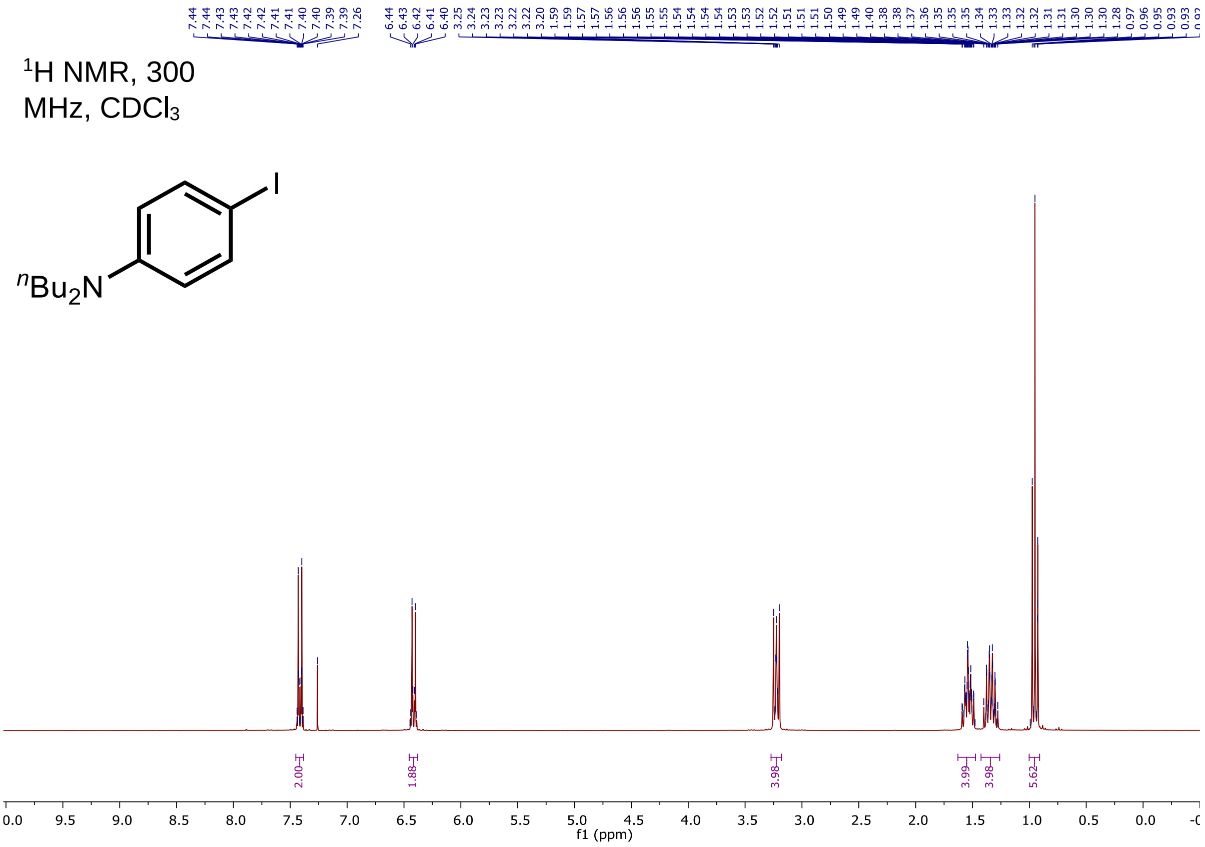

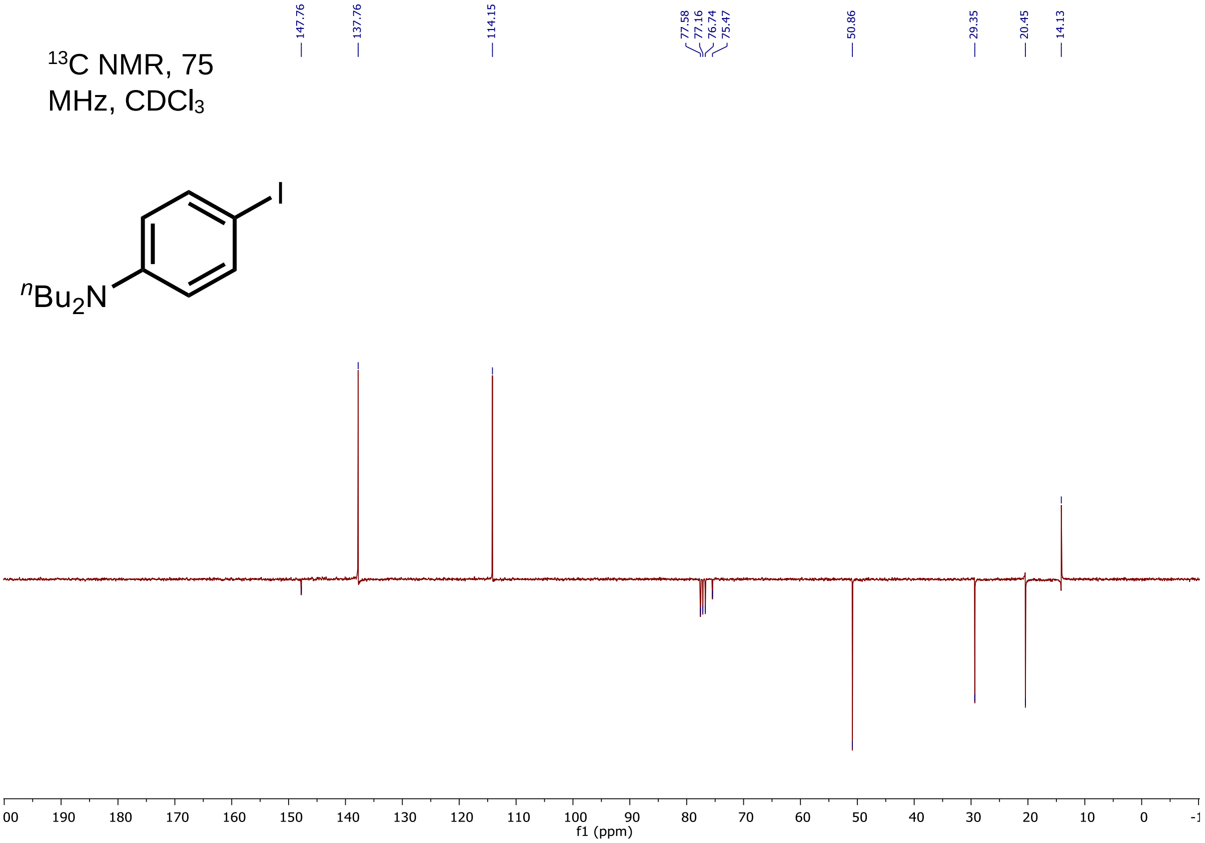

5-Iodo-1-methyl-1*H*-indole[8]

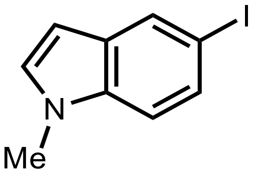

Under nitrogen, 6-iodoindole (5 mmol, 1.21 g) was added to dry DMF (15 mL) in 100 mL round-bottomed flask. NaH 60% in mineral oil (6 mmol, 0.24 g) was added slowly to the mixture and stirred at rt for 1 h. MeI (10 mmol, 0.62 mL) was added then stirred at rt for overnight. Water (10 mL) was added slowly, extracted with EtOAc (1 x 10 mL), washed with brine (3 x 10 mL), dried over $MgSO_4$, filtered, and concentrated *in vacuo*. Purification by flask silica chromatography (eluent = 20% EtOAc in PE) gave the title compound as pale-yellow solid (1.1 g, 86%); mp 73–74 ˚C (lit. 74–75 ˚C)[9]; Rf: 0.56 (eluent = 20% EtOAc in PE); **^{1}H NMR (300 MHz, Chloroform-*d*)** δ 7.97 (dd, *J* = 1.7, 0.6 Hz, 1H), 7.47 (ddd, *J* = 8.6, 1.7, 0.4 Hz, 1H), 7.10 (dt, *J* = 8.6, 0.7 Hz, 1H), 7.01 (d, *J* = 3.1 Hz, 1H), 6.42 (dd, *J* = 3.1, 0.9 Hz, 1H), 3.76 (s, 3H); **^{13}C NMR (75 MHz, Chloroform-*d*)** δ 135.9, 131.1, 129.9, 129.7, 111.3, 100.4, 83.0, 33.0.

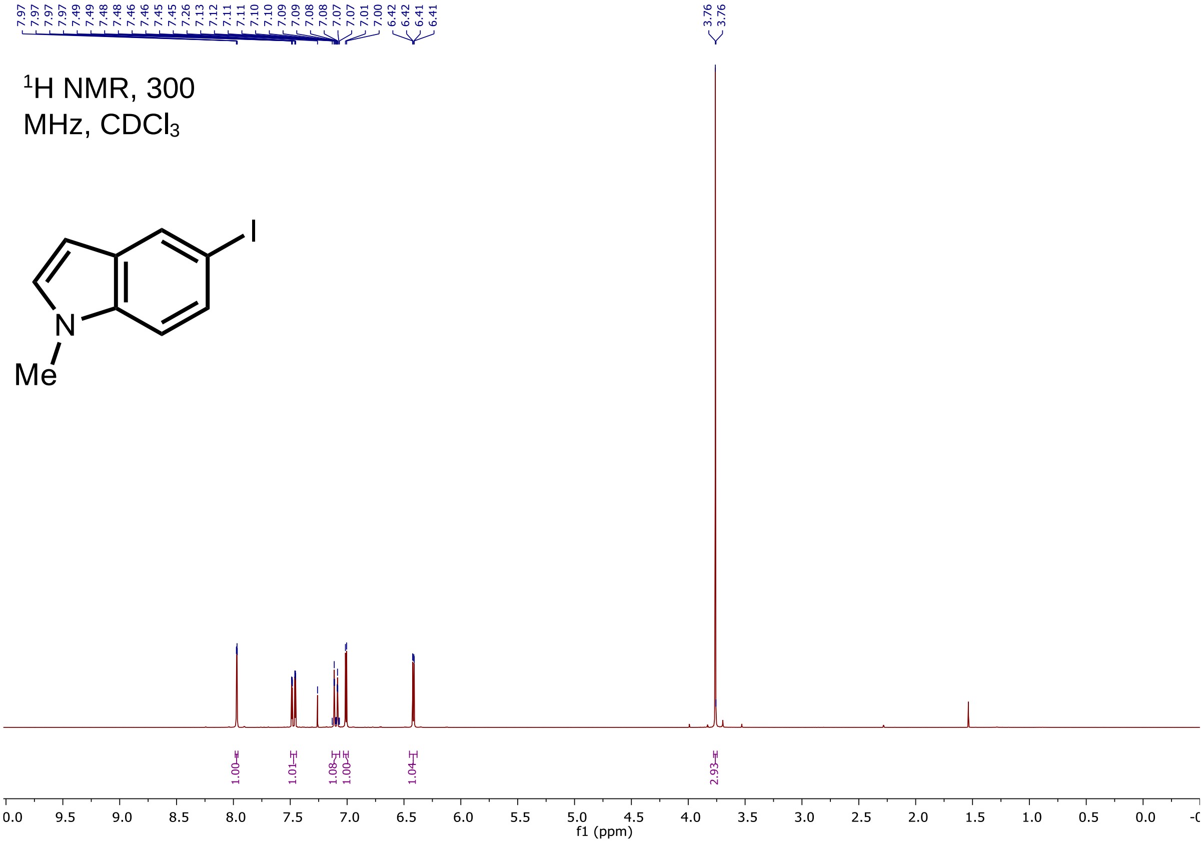
^{1}H NMR, 300 MHz, $CDCl_3$
Me
N
I
7.97 7.97 7.97 7.97 7.49 7.49 7.48 7.48 7.46 7.46 7.45 7.45 7.26 7.13 7.12 7.11 7.11 7.10 7.10 7.09 7.09 7.08 7.08 7.07 7.07 7.01 7.00 6.42 6.42 6.41 6.41
3.76 3.76
1.00 1.01 1.08 1.00 1.04 2.93
f1 (ppm)

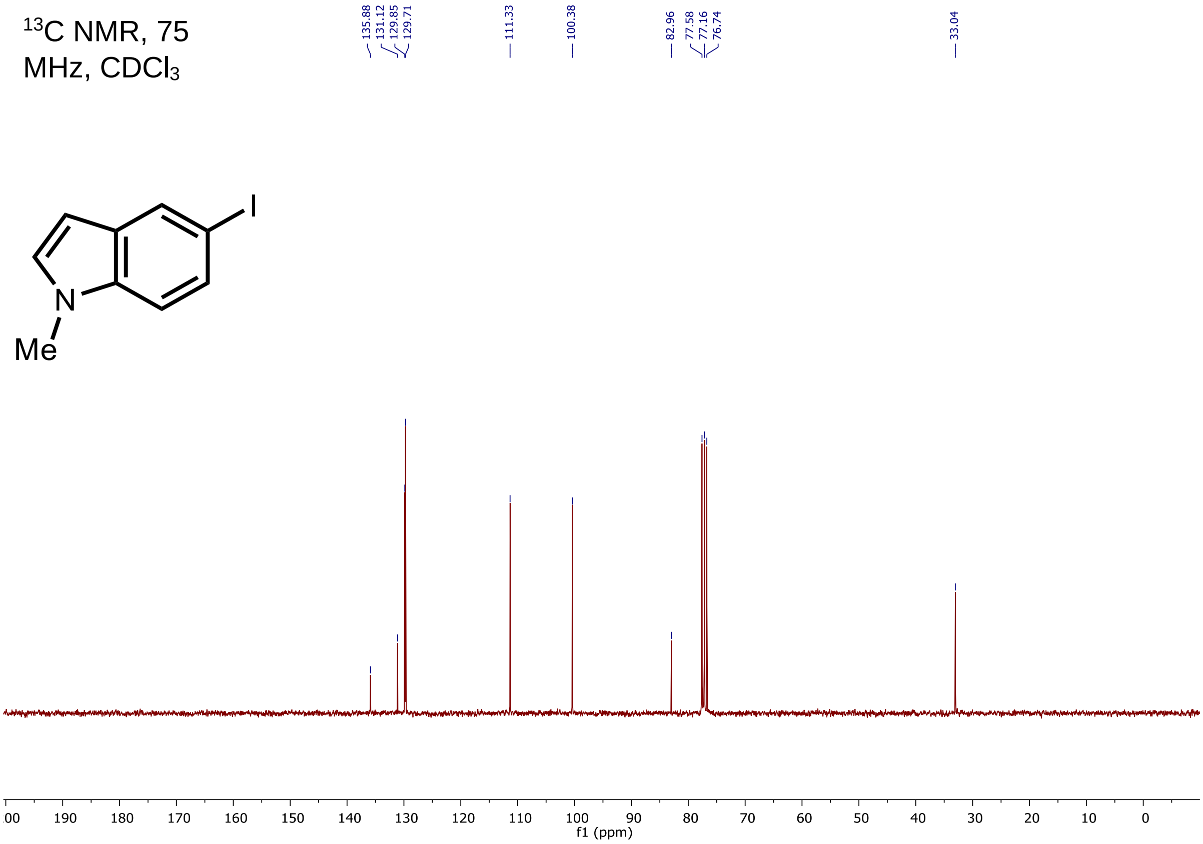
^{13}C NMR, 75 MHz, $CDCl_3$
Me
N
I
135.88 131.12 129.85 129.71
111.33
100.38
82.96 77.58 77.16 76.74
33.04
f1 (ppm)

5-Iodo-1-tosyl-1*H*-indole[10]

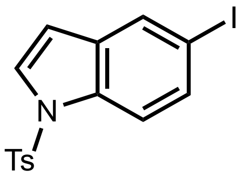

6-iodoindole (5 mmol, 1.21 g) was added to dry DMF (15 mL) in 100 mL round-bottomed flask. NaH 60% in mineral oil (6 mmol, 0.24 g) was added slowly to the mixture and stirred at rt for 1 h. *p*-Toluenesulfonyl chloride (10 mmol, 1.90 g) was added to the mixture slowly then stirred at rt for overnight. Water (10 mL) was added slowly, extracted with EtOAc (1 x 10 mL), washed with brine (3 x 10 mL), dried over $MgSO_4$, filtered, and concentrated *in vacuo*. Purification by flask silica chromatography (eluent = 20% EtOAc in PE) gave the title compound as white solid (0.99 g, 50%); mp 141–142 ˚C (lit. 136–138 ˚C)[e]; Rf: 0.52 (eluent = 20% EtOAc in PE); **^{1}H NMR (500 MHz, Chloroform-*d*)** δ 7.87 (d, *J* = 1.7 Hz, 1H), 7.77 – 7.72 (m, 3H), 7.57 (dd, *J* = 8.7, 1.7 Hz, 1H), 7.52 (d, *J* = 3.7 Hz, 1H), 7.25 – 7.20 (m, 2H), 6.57 (dd, *J* = 3.7, 0.7 Hz, 1H), 2.35 (s, 3H); **^{13}C NMR (126 MHz, Chloroform-*d*)** δ 145.4, 135.2, 134.2, 133.2, 130.4, 130.1, 127.3, 126.9, 115.5, 108.1, 87.6, 21.7.

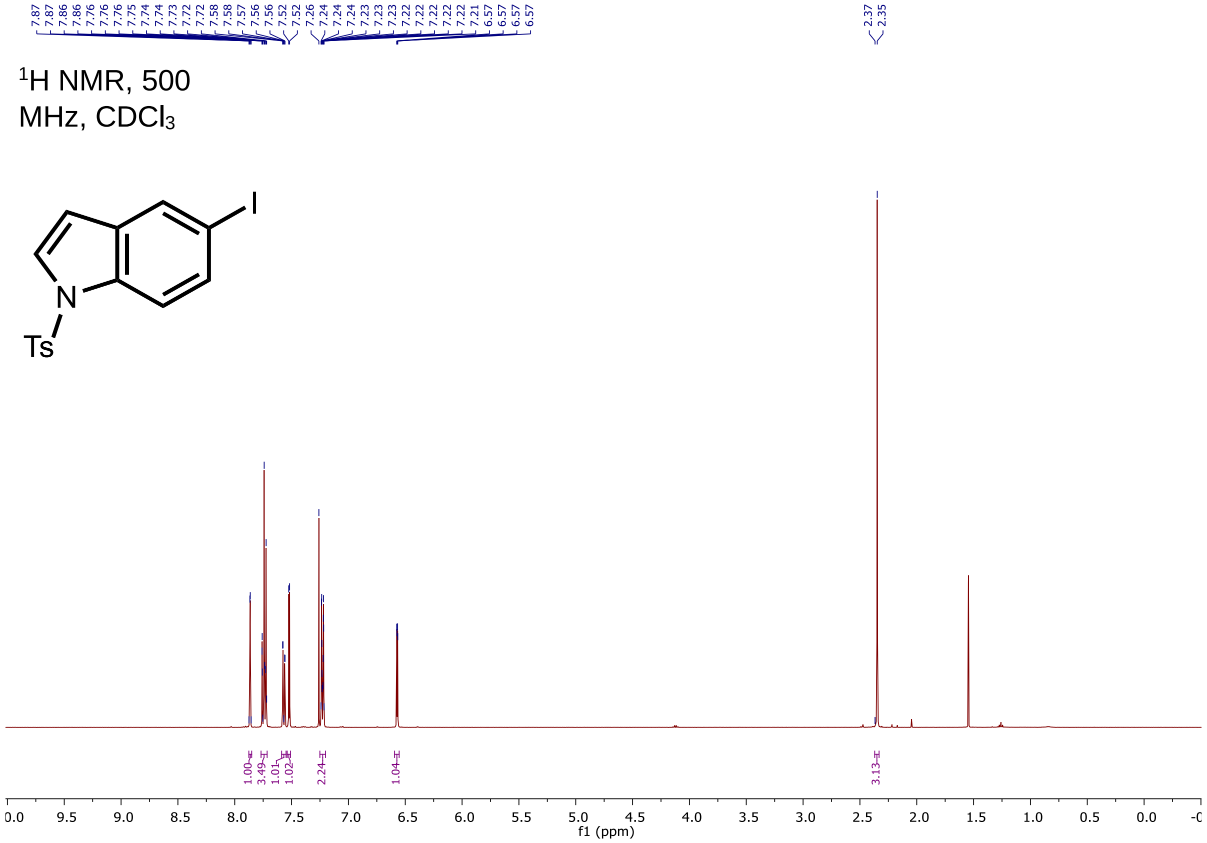

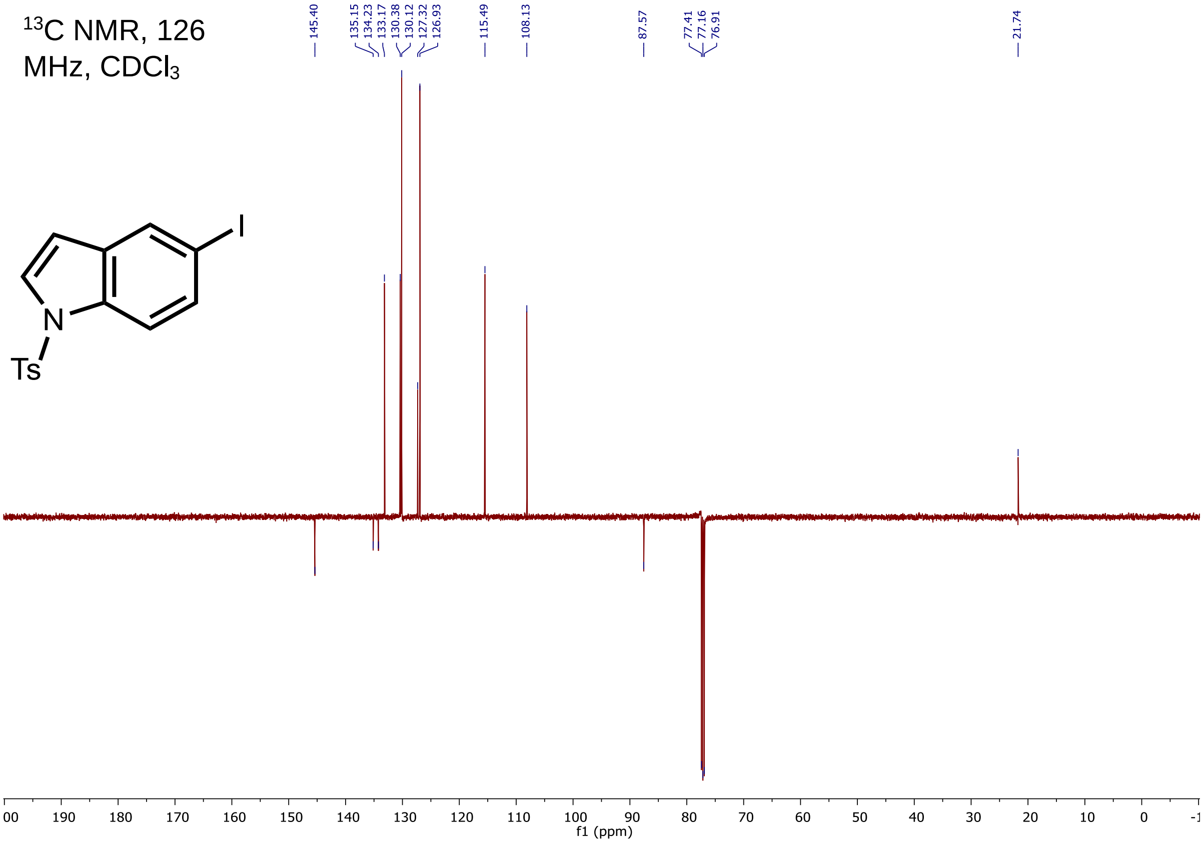

1-(Benzyloxy)-4-iodobenzene[10]

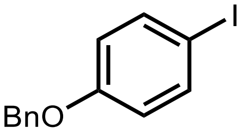

4-iodophenol (10 mmol, 2.20 g), K_2CO_3 (25 mmol, 3.45 g) were added to acetone in 100 mL round-bottomed flask. Benzyl bromide (10 mmol, 1.20 mL) was added and refluxed for overnight. The mixture was cooled to rt then water was added. The mixture was extracted with EtOAc (1 x 10 mL), washed with brine (1 x 10 mL), dried over $MgSO_4$, filtered, and concentrated *in vacuo*. Purification by flask silica chromatography (eluent = 100% PE) gave the title compound as white solid (2.9 g, 95%); mp 62–63 ˚C (lit. 61–63 ˚C)[11]; Rf: 0.41 (eluent = 100% PE); **^{1}H NMR (300 MHz, Chloroform-*d*)** δ 7.59 – 7.52 (m, 2H), 7.44 – 7.29 (m, 5H), 6.78 – 6.72 (m, 2H), 5.03 (s, 2H); **^{13}C NMR (75 MHz, Chloroform-*d*)** δ 138.4, 136.6, 135.7, 128.8, 128.3, 127.6, 117.4, 115.7, 70.2.

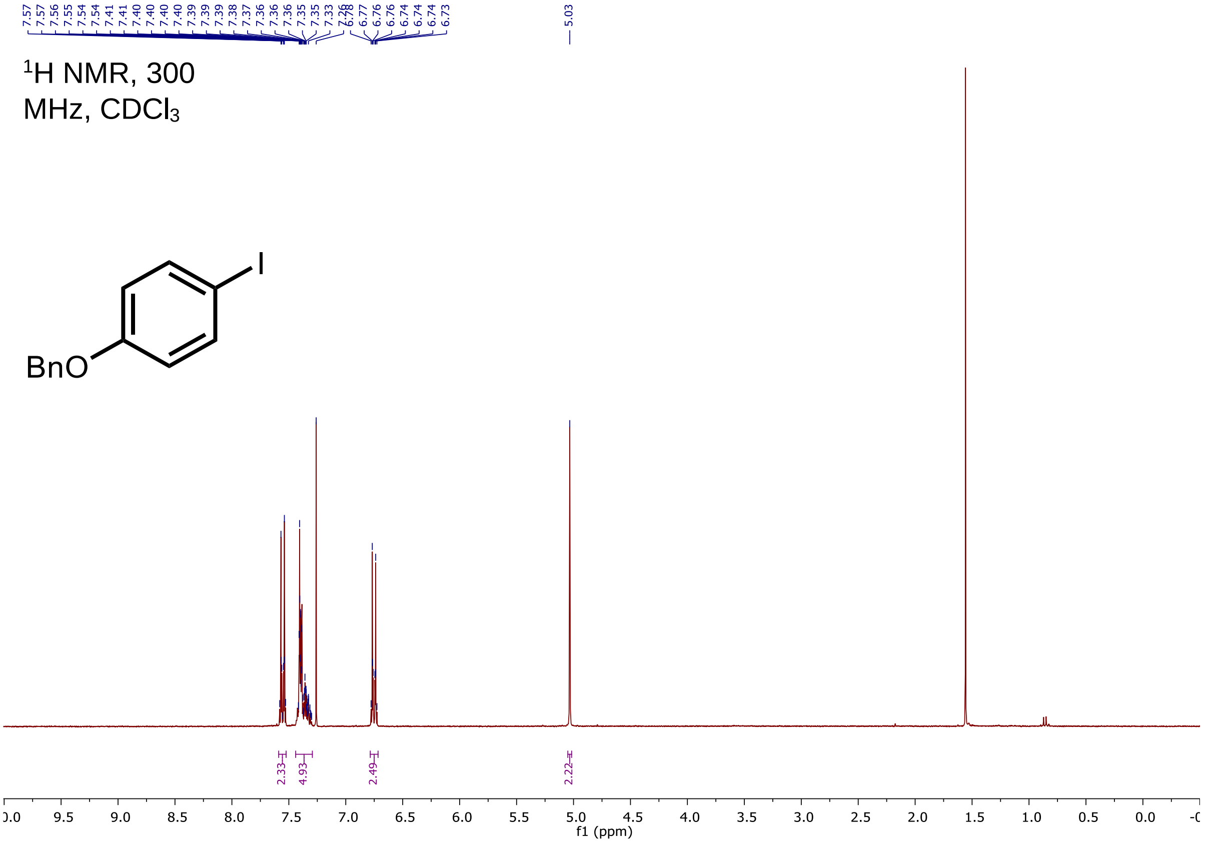

^{1}H NMR, 300 MHz, CDCl$_3$
BnO
I
5.03
2.33
4.93
2.49
2.22
f1 (ppm)

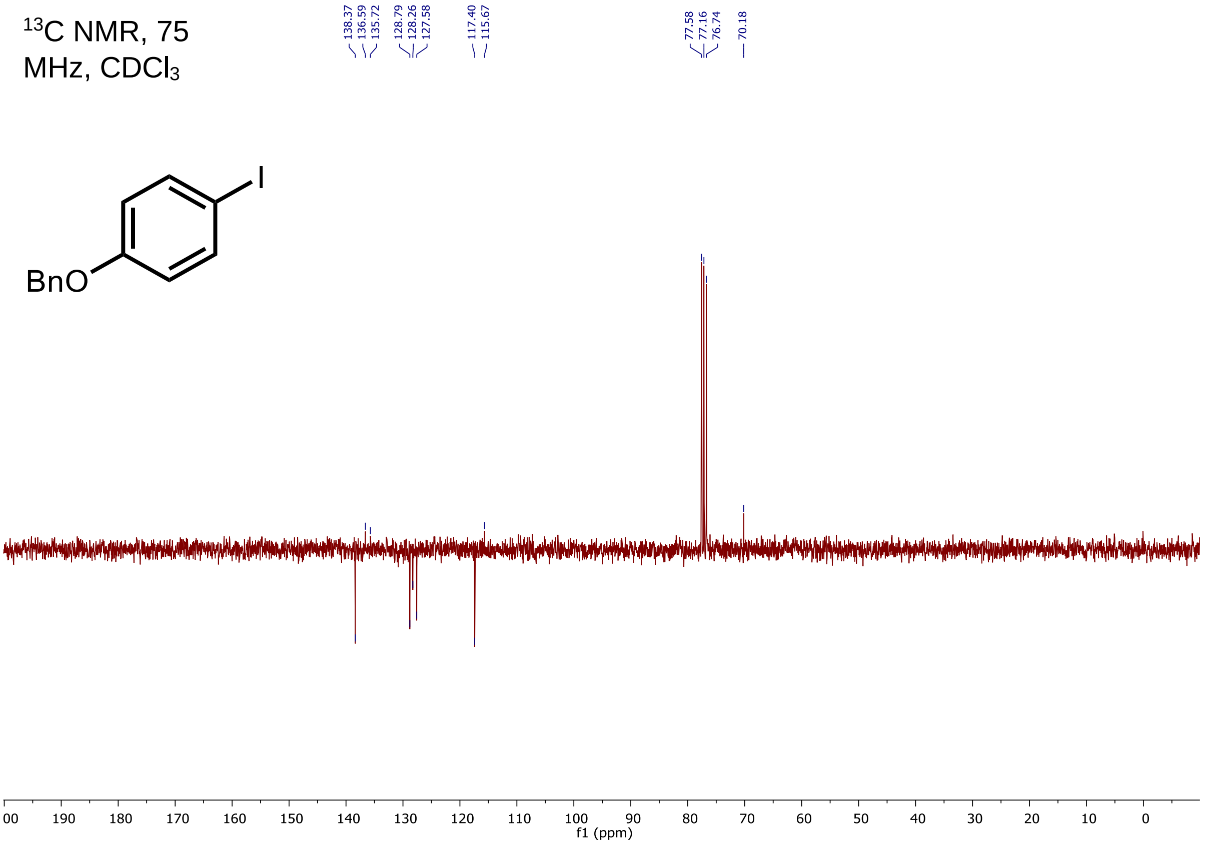

^{13}C NMR, 75 MHz, CDCl$_3$
BnO
I
138.37
136.59
135.72
128.79
128.26
127.58
117.40
115.67
77.58
77.16
76.74
70.18
f1 (ppm)

1-Iodo-4-(phenoxymethyl)benzene[12]

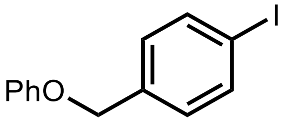

Phenol (5 mmol, 0.47 g), K_2CO_3 (25 mmol, 3.45 g) were added to acetone in 100 mL round-bottomed flask. 4-iodobenzyl bromide (5 mmol, 1.48 g) was added and refluxed for overnight. the mixture was cooled to rt then water was added. The mixture was extracted with EtOAc (1 x 10 mL), washed with brine (1 x 10 mL), dried over $MgSO_4$, filtered, and concentrated *in vacuo*. Purification by flask silica chromatography (eluent = 5% EtOAc in PE) gave the title compound as white solid (1.00 g, 65%); mp 99–100 ˚C (lit. 97.7–100.3 ˚C)[12]; Rf: 0.59 (eluent = 5% EtOAc in PE); **^{1}H NMR (300 MHz, Chloroform-*d*)** δ 7.74 – 7.68 (m, 2H), 7.33 – 7.26 (m, 2H), 7.21 – 7.16 (m, 2H), 7.01 – 6.92 (m, 3H), 5.01 (s, 2H); **^{13}C NMR (75 MHz, Chloroform-*d*)** δ 159.0, 138.4, 137.8, 129.7, 129.4, 121.3, 114.9, 93.6, 69.3.

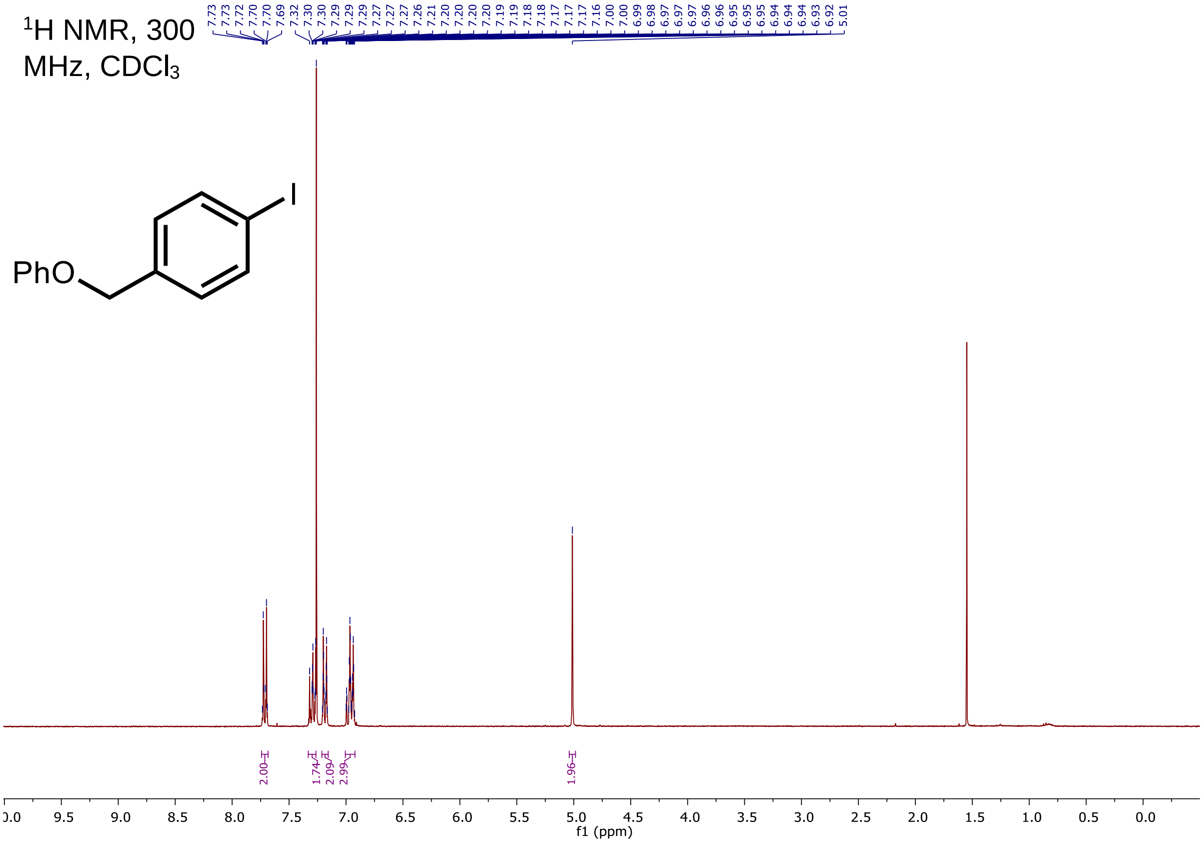

^{13}C NMR, 75 MHz, $CDCl_3$

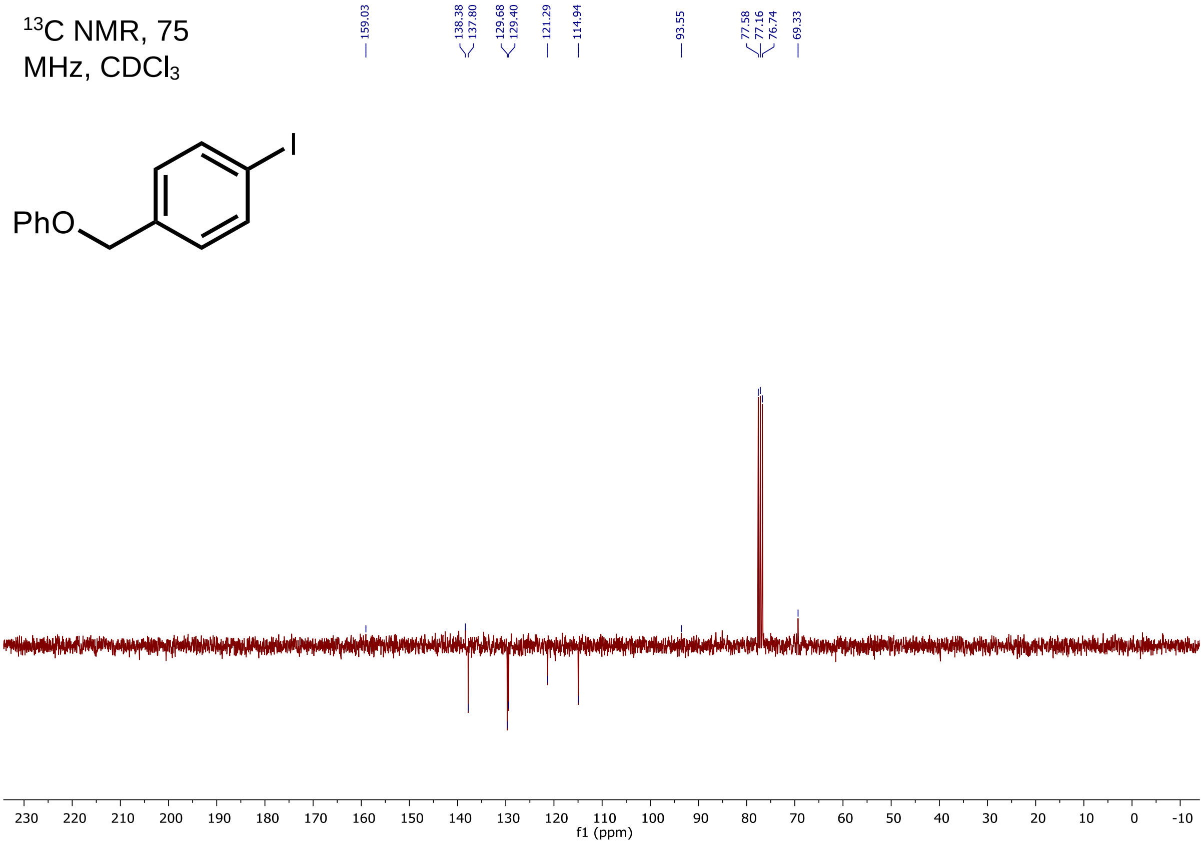

Butyl 2-(4-iodophenyl)acetate

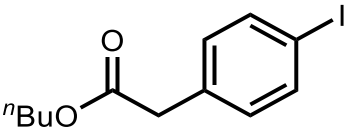

4-iodophenylacetic acid (5 mmol, 1.31 g), K_2CO_3 (25 mmol, 3.45 g) were added to acetone in 100 mL round-bottomed flask. 1-bromobutane (10 mmol, 1.1 mL) was added and refluxed for overnight. The mixture was cooled to rt then water was added. The mixture was extracted with EtOAc (1 x 10 mL), washed with brine (1 x 10 mL), dried over $MgSO_4$, filtered, and concentrated *in vacuo*. Purification by flask silica chromatography (eluent = 10% EtOAc in PE) gave the title compound as pale-yellow liquid (0.72 g, 45%); Rf: 0.47 (eluent = 10% EtOAc in PE); **^{1}H NMR (300 MHz, Chloroform-*d*)** δ 7.68 – 7.61 (m, 2H), 7.07 – 7.00 (m, 2H), 4.09 (t, *J* = 6.7 Hz, 2H), 3.55 (s, 2H), 1.66 – 1.53 (m, 2H), 1.41 – 1.27 (m, 2H), 0.91 (t, *J* = 7.3 Hz, 3H); **^{13}C NMR (75 MHz, Chloroform-*d*)** δ 171.2, 137.7, 133.9, 131.4, 92.7, 65.1, 41.1, 30.7, 19.2, 13.8; **IR** (film, ν_{max} / cm^{-1}) 2957, 2932, 2872, 1730, 1485, 1400, 1248, 1155, 1061, 797; **HRMS** (CI) calculated $[C_{12}H_{15}IO_2]^+$ $(M)^+$: m/z 318.0111, found 318.0108.

^{1}H NMR, 300 MHz, $CDCl_3$

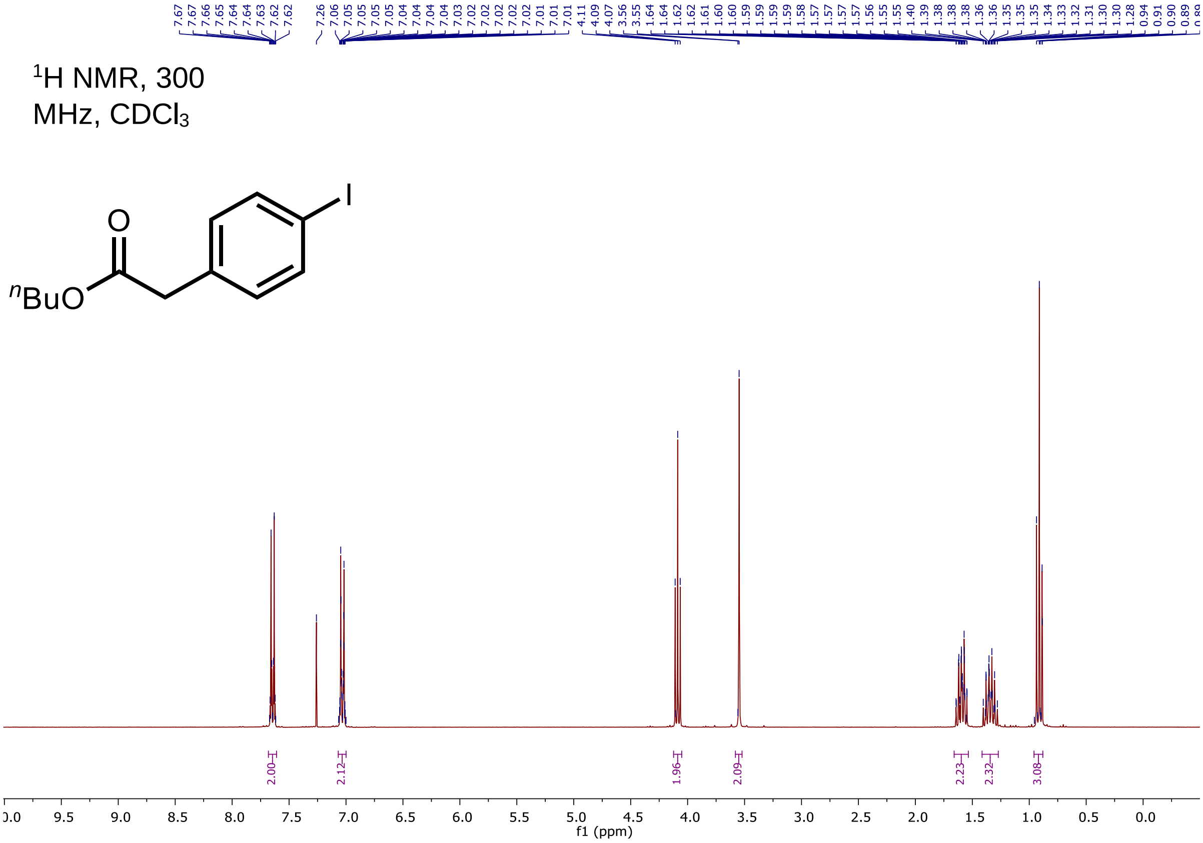

^{13}C NMR, 75 MHz, $CDCl_3$

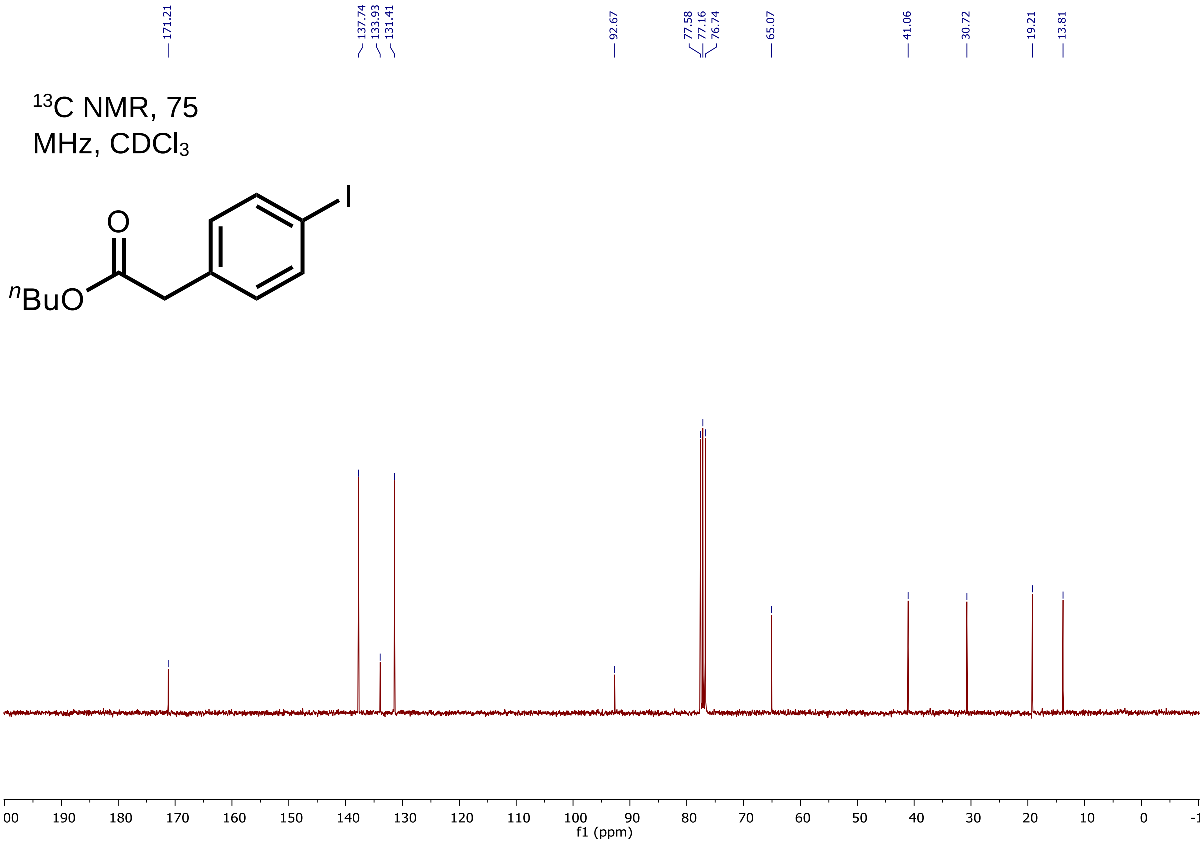

Phenyl 4-iodobenzoate[13]

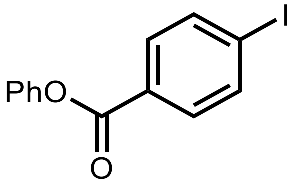

Under N_2, 4-iodobenzoyl chloride (5 mmol, 1.33 g) and phenol (5 mmol, 0.47 g) were added to dry THF (20 mL). Et_3N (20 mmol, 2.8 mL) was added then refluxed for overnight. The mixture was cooled to rt then water was added. The organic phase was separated, washed with brine (1 x 10 mL), dried over $MgSO_4$, filtered, and concentrated *in vacuo*. Purification by flask silica chromatography (eluent = 10% EtOAc in PE) gave the title compound as pale yellow solid (1.13 g, 70%); mp 131–132 ˚C (lit. 130–132 ˚C)[13]; Rf: 0.46 (eluent = 10% EtOAc in PE); **^{1}H NMR (500 MHz, Chloroform-*d*)** δ 7.93 – 7.86 (m, 4H), 7.46 – 7.41 (m, 2H), 7.31 – 7.26 (m, 1H), 7.24 – 7.18 (m, 2H); **^{13}C NMR (126 MHz, Chloroform-*d*)** δ 164.9, 150.9, 138.1, 131.7, 129.7, 129.2, 126.2, 121.8, 101.7.

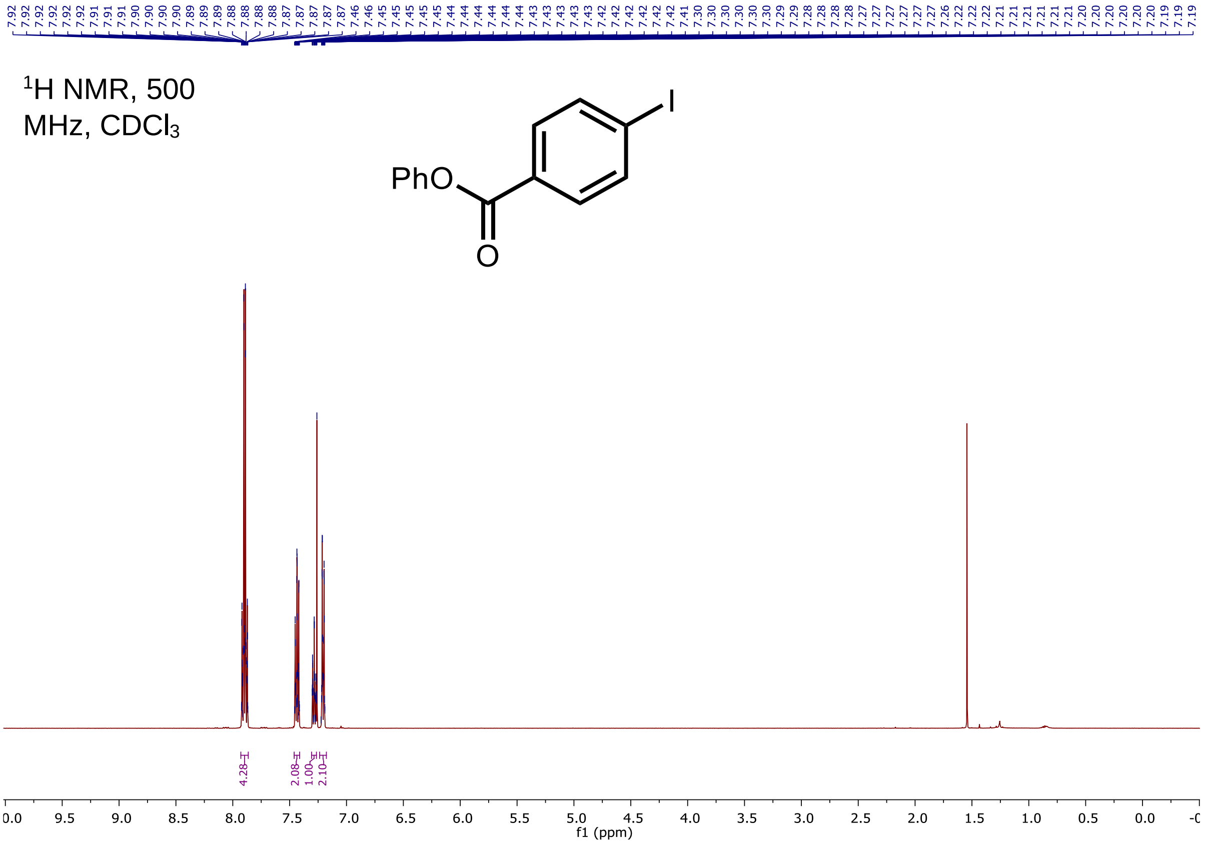

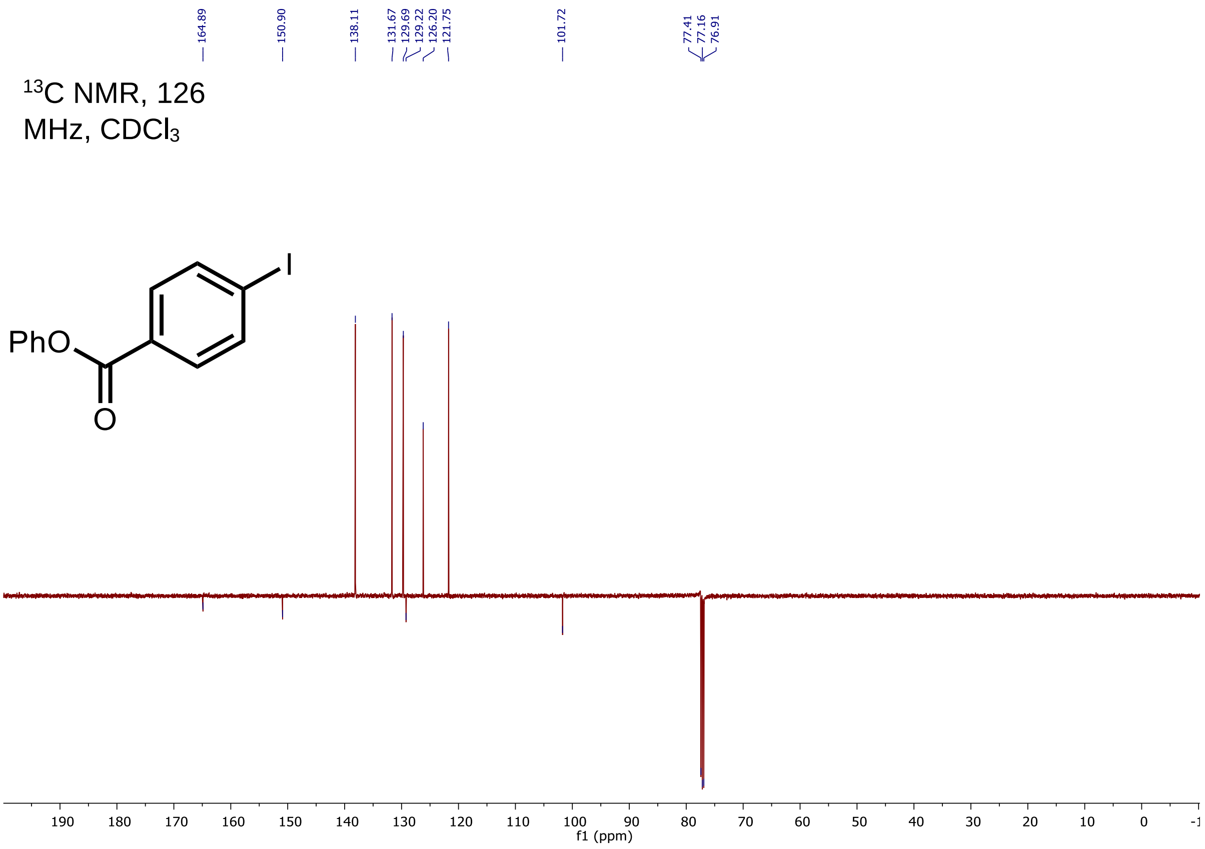

4-Iodo-*N*,*N*-dimethylbenzenesulfonamide[14]

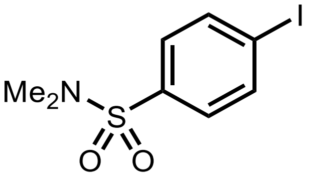

4-Iodobenzenesulfonamide (5 mmol, 1.42 g), NaOH (30 mmol, 1.20 g), and K_2CO_3 (30 mmol, 4.20 g) were added to dry DMF (20 mL) in 100 mL round-bottomed flask. MeI (20 mmol, 1.87 mL) was added to the mixture then stirred at 70 ˚C for 2 days. The reaction was cooled to rt then water (20 mL) was added. The mixture was extracted with EtOAc (1 x 10 mL), washed with brine (3 x 10 mL), dried over $MgSO_4$, filtered, and concentrated *in vacuo*. Purification by flask silica chromatography (eluent = 1:1 EtOAc in PE) gave the title compound as white solid (1.1 g, 70%); mp 135–136 ˚C (lit. 134–135 ˚C)[14]; Rf: 0.59 (eluent = 1:1 EtOAc in PE); **^{1}H NMR (300 MHz, Chloroform-*d*)** δ 7.93 – 7.88 (m, 2H), 7.52 – 7.46 (m, 2H), 2.71 (s, 6H); **^{13}C NMR (75 MHz, Chloroform-*d*)** δ 138.4, 135.5, 129.2, 100.3, 38.0.

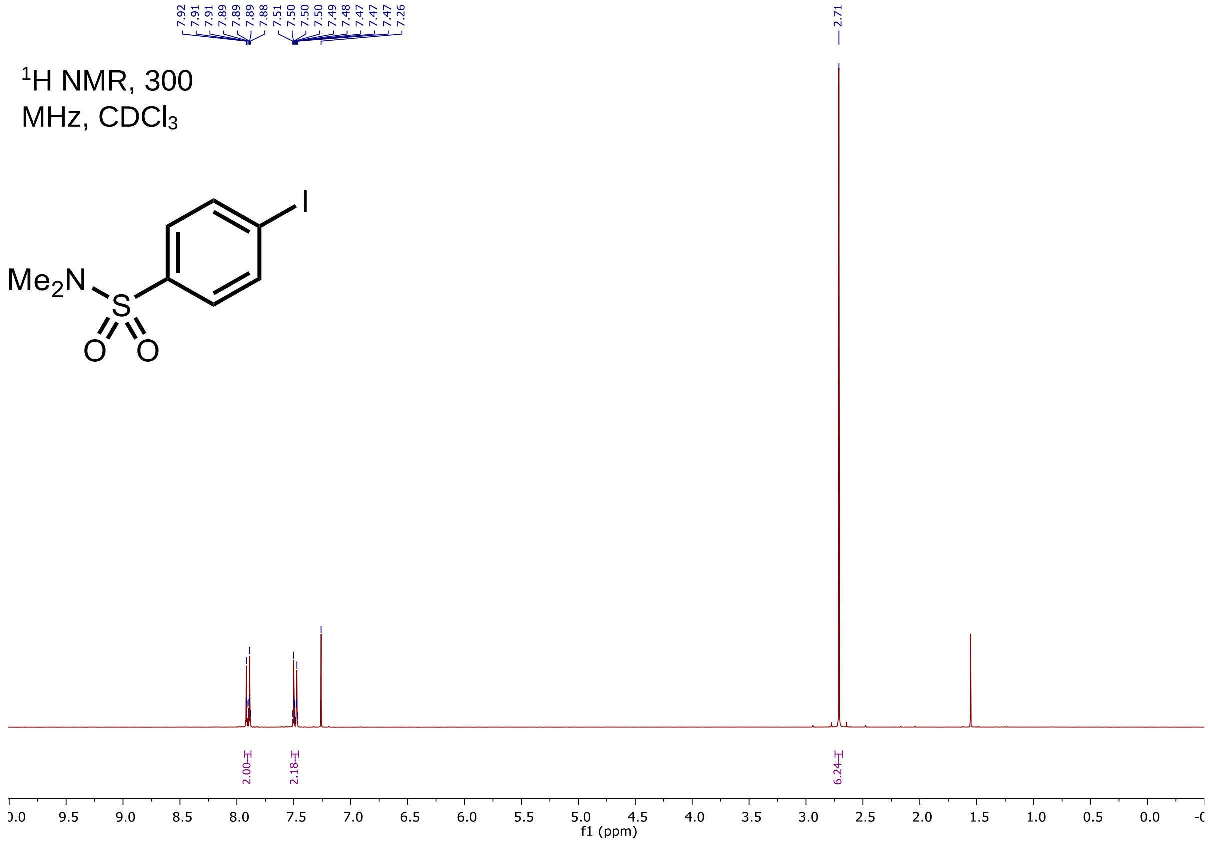

1H NMR, 300 MHz, CDCl3
Me2N
S
O
O
I
7.92
7.91
7.91
7.89
7.89
7.89
7.88
7.51
7.50
7.50
7.50
7.49
7.48
7.47
7.47
7.47
7.26
2.71
2.00
2.18
6.24
f1 (ppm)

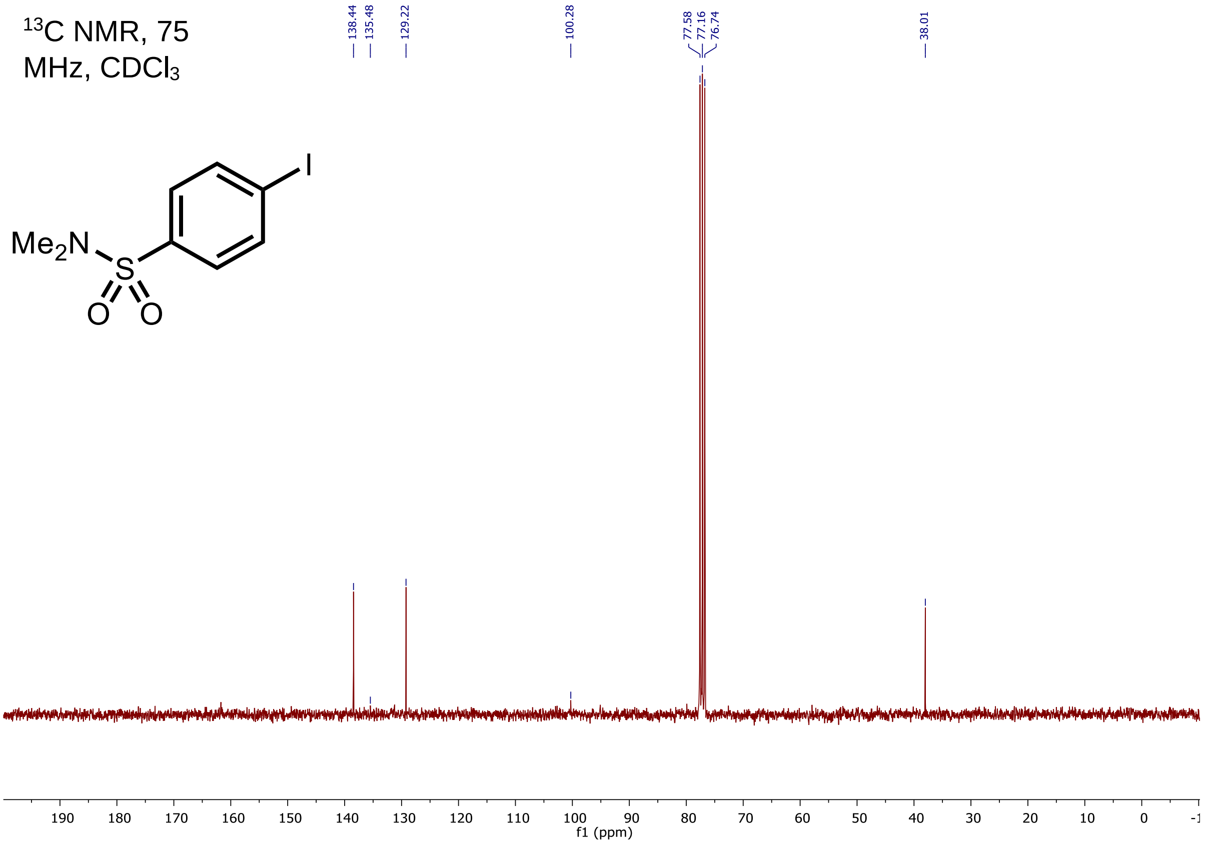

13C NMR, 75 MHz, CDCl3
Me2N
S
O
O
I
138.44
135.48
129.22
100.28
77.58
77.16
76.74
38.01
f1 (ppm)

2.2. Optimizations

2.2.1. Optimization of allylsilane isomerization

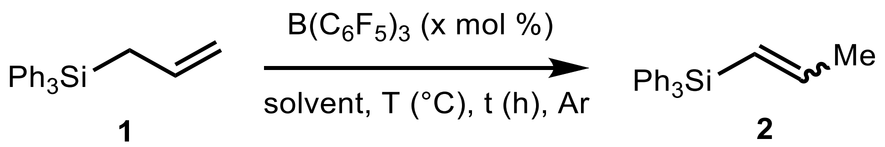

In the glovebox under Ar, an oven-dried 10 mL microwave vial equipped with a magnetic stirrer bar was charged with $B(C_6F_5)_3$ (x mol%), allyltriphenylsilane (0.1 mmol), and solvent (0.4 mL). The vial was sealed with an aluminium crimp cap and removed from the glovebox then stirred at the stated temperature (˚C) and time (h). It was then cooled to rt, 1,3,5-trimethylbenzene (30 μL, 0.2 mmol) and wet solvent (0.2 mL) were added. The mixture was stirred for 5 minutes and analysed using ^{1}H NMR.

Entry	SM (mmol)	BCF (mol%)	Solvent	T (˚C)	t (h)	Results (%)[a]		
						SM	*E*/*Z*	Total
1	0.2	20	toluene	150	24	4	91:9	57
2	0.2	10	toluene	150	24	4	92:8	70
3	0.2	5	toluene	150	24	8	94:6	76
4	0.2	10	toluene	150	48	1	91:9	69
5	0.2	5	toluene	150	48	7	93:7	85(80)[b]
6	0.2	2	toluene	150	48	92	93:7	7
7	0.2	5	xylene	150	48	3	91:9	79
8	0.2	5	nitrobenz	150	48	>95	n.d	<2
9	0.2	5	chlorobenz	150	48	8	94:6	77
10	0.2	5	Bromobenz	150	48	6	92:8	72
11	0.2	5	mesitylene	150	48	72	92:8	25
12	0.2	5	anisole	150	48	72	92:8	12
13	0.2	10	toluene	140	24	6	93:7	66
14	0.2	5	toluene	140	24	8	95:5	75
15	0.2	5	toluene	140	48	8	95:5	72
16	0.2	10	toluene	130	24	6	97:3	68
17	0.2	5	toluene	130	24	8	98:2	78
18	0.2	5	toluene	130	48	8	98:2	75
19	0.2	5	toluene	120	24	33	98:2	45
20	0.2	5	toluene	120	48	33	98:2	45
21	0.1 [0.25M]	10	toluene	150	24	7	93:7	69
22	0.1 [0.25M]	5	toluene	150	24	15	97:3	68
23	0.1 [0.5M]	5	toluene	150	48	9	94:6	75
24	0.1 [0.25M]	10	toluene	140	24	6	93:7	72
25	0.1 [0.25M]	5	toluene	140	24	12	99:1	72
26	0.1 [0.25M]	5	toluene	140	48	8	97:3	85(80)[b]
27	0.1 [0.25M]	5	toluene	130	24	81	84:16	6
28	0.1 [0.5M]	5	toluene	140	48	6	94:6	72
29	0.04 [0.1M]	5	toluene	140	48	4	98:2	65

30	0.1 [0.25M]	0	toluene	140	48	>95	n.d	<2
31	0.1 [0.25M]	5	xylene	140	48	50	>98:<2	30
32	0.1 [0.25M]	5	nitrobenz	140	48	90	n.d	<2
33	0.1 [0.25M]	5	bromobenz	140	48	3	96:4	55
34	0.1 [0.25M]	5	chlorobenz	140	48	5	96:4	76
35	0.1 [0.25M]	5	anisole	140	48	81	>98:<2	7
36[c]	0.1 [0.25M]	5	mesitylene	140	48	94	>98:<2	6

[a]determined by crude NMR yield with 1,3,5-trimethylbenzene as internal standard; [b]isolated yield; [c]determined by crude NMR yield with anisole as internal standard; SM = starting material; n.d.: not determined.

2.2.2. Optimization of one-pot isomerization/Hiyama cross-coupling

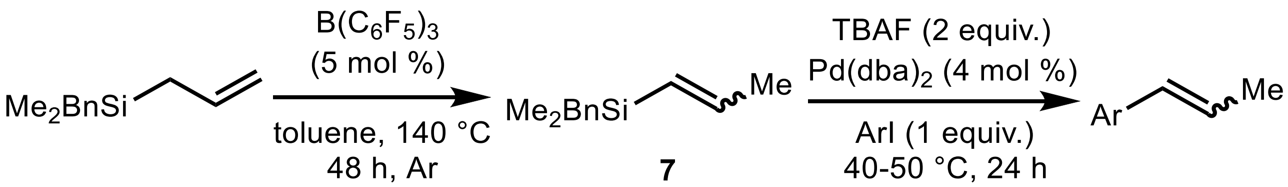

Entry	Allylsilane (mmol)	Pd(dba) (mol%)	T (˚C)	Results (%)[a]				
				Allyl silane	Me-vinyl silane	Ar-I	*E*:*Z*	Coupling product
1	0.1	2.5	rt	<2	22	43	88:12	57
2	0.1	4	rt	<2	30	40	85:15	59
3	0.1	2.5	40	<2	23	33	85:15	67
4	0.1	4	40	<2	<2	9	87:13	91 (85)[b]

[a]determined by crude NMR yield with 1,3,5-trimethylbenzene as internal standard; [b]isolated yield; ArI = 1-iodo-4-(phenoxymethyl)benzene.

2.3. Substrate scope

2.3.1. General procedure 2

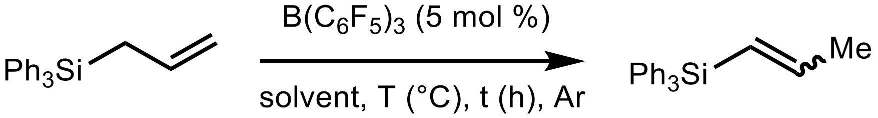

In the glovebox under Ar, an oven-dried 10 mL microwave vial equipped with a magnetic stirrer bar was charged with $B(C_6F_5)_3$ (5 mol %), allylsilane (0.1 mmol), and toluene (0.4 mL). The vial was sealed with an aluminium crimp cap and stirred at 140 ˚C for 48 h. It was cooled to rt, 1,3,5-trimethylbenzene (15 μL, 0.1 mmol) was added and analysed using ^{1}H NMR. Purification was done by quenching with brine (0.4 mL). The organic phase was separated, dried over $MgSO_4$, filtered, and concentrated *in vacuo*.

2.3.2. Characterization of allylsilane isomerization products

(*E*)-Triphenyl(prop-1-en-1-yl)silane (2)[15]

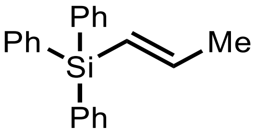

The title compound was prepared according to general procedure 2 using allyltriphenylsilane (0.1 mmol). Purification by flash silica chromatography (eluent = 100% PE) gave the title compound as a white solid (24 mg, 80%, *E*:*Z* = 95:5); mp 87–88 ˚C (lit. 90–92 ˚C)[16]; R_f = 0.40 (eluent = 100% PE). NMR yield = 85% (*E*:*Z* = 97:3).

<u>Signals of the major isomer (*E*)-triphenyl(prop-1-en-1-yl)silane:</u>

^{1}H NMR (300 MHz, Chloroform-*d*) δ 7.59 – 7.54 (m, 6H), 7.44 – 7.38 (m, 9H), 6.24 (d, *J* = 3.8 Hz, 2H), 2.05 – 1.89 (m, 3H).

^{13}C NMR (75 MHz, Chloroform-*d*) δ 148.5, 136.1, 135.1, 129.5, 127.9, 125.2, 23.1.

<u>Resolved signals of the minor isomer (*Z*)-triphenyl(prop-1-en-1-yl)silane:</u>

^{1}H NMR (300 MHz, Chloroform-*d*) δ 6.87 (dq, *J* = 13.7, 6.9 Hz, 1H), 6.12 (dq, *J* = 14.0, 1.5 Hz, 1H), 1.66 (dd, *J* = 6.9, 1.5 Hz, 3H).

NMR yield determination:

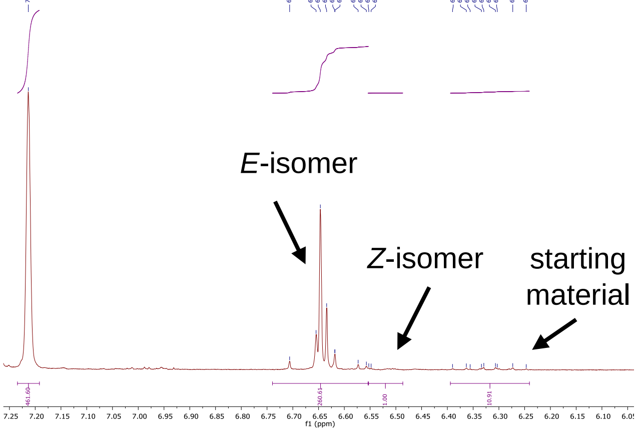

$$\% \text{ yield} = \frac{cmp\ \ integral}{std\ integral} \times \frac{std\ proton}{cmp\ proton} \times 100$$

$$E\text{-isomer} = \frac{260.61}{461.60} \times \frac{3}{2} \times 100 = 84\%$$

$$Z\text{-isomer} = \frac{1.00}{461.60} \times \frac{3}{1} \times 100 = 1\%$$

cmp = compound

std = standard (1,3,5-trimethylbenzene)

^{1}H NMR, 300 MHz, $CDCl_3$

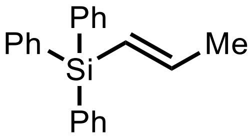

2

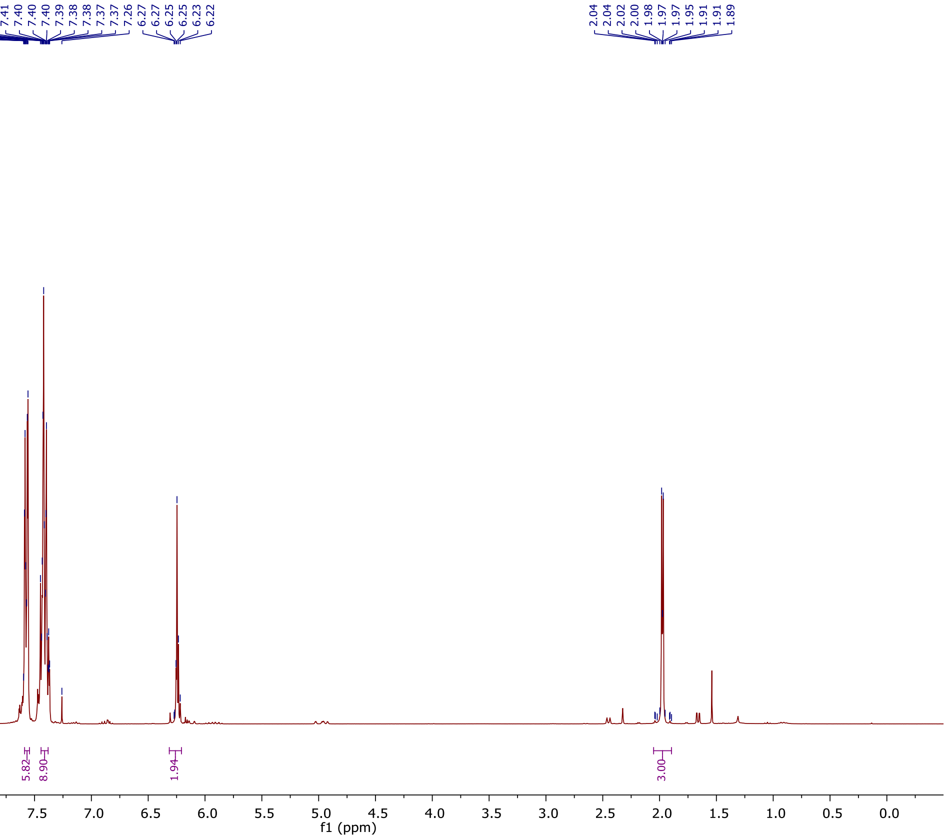

^{13}C NMR, 75 MHz, $CDCl_3$

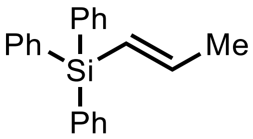

2

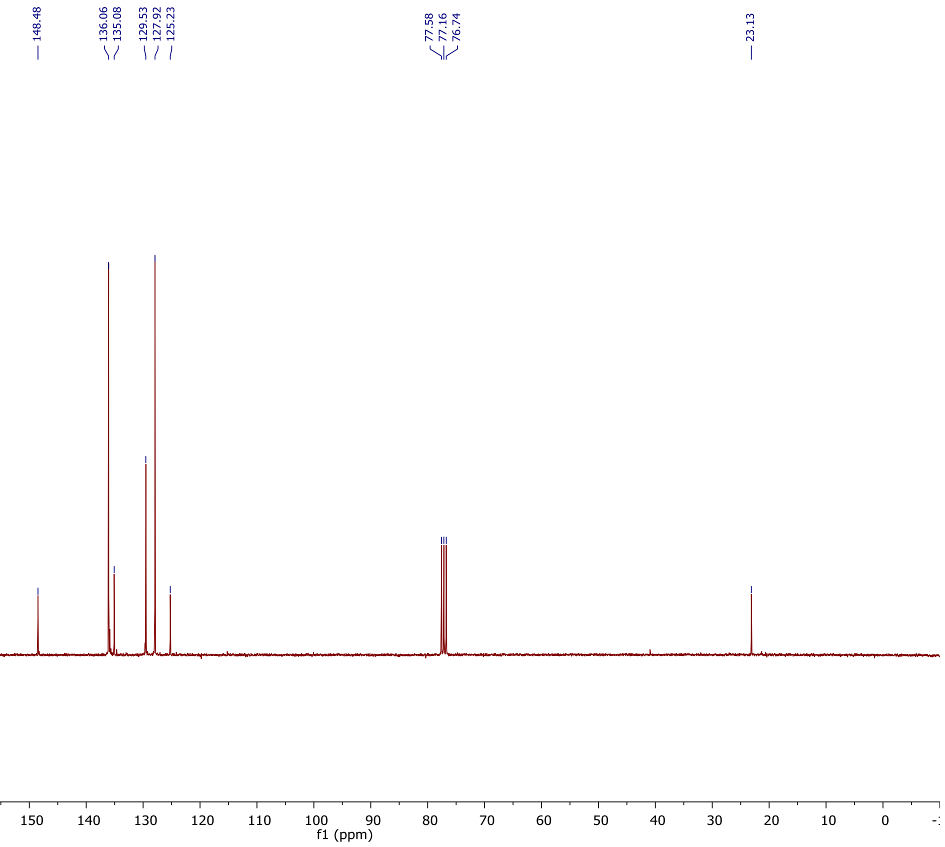

(*E*)-*tert*-Butyldiphenyl-1-propenylsilane (3) [17]

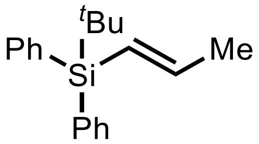

The title compound was prepared according to general procedure 2 using 3-(*tert*-butyldiphenylsilyl)propene (0.1 mmol). Purification by flash silica chromatography (eluent = 100% PE) gave the title compound as a colourless liquid (24.6 mg, 88%, *E*:*Z* = >98:<2); R_f = 0.39 (eluent = 100% PE). NMR yield = 91% (*E*:*Z* = 98:2).

Signals of the major isomer (*E*)-*tert*-butyldiphenyl(prop-1-en-1-yl)silane:

^{1}H NMR (300 MHz, Chloroform-*d*) δ 7.65 – 7.60 (m, 4H), 7.44 – 7.31 (m, 6H), 6.12 – 6.04 (m, 2H), 1.97 – 1.88 (m, 3H), 1.09 (s, 9H).

^{13}C NMR (75 MHz, Chloroform-*d*) δ 147.9, 135.3, 129.1, 127.6, 124.7, 27.9, 23.2, 18.3.

Resolved signals of the minor isomer (*Z*)-*tert*-butyldiphenyl(prop-1-en-1-yl)silane:

^{1}H NMR (300 MHz, Chloroform-*d*) δ 6.82 (dd, *J* = 14.1, 7.0 Hz, 1H), 1.43 (dd, *J* = 6.9, 1.5 Hz, 3H).

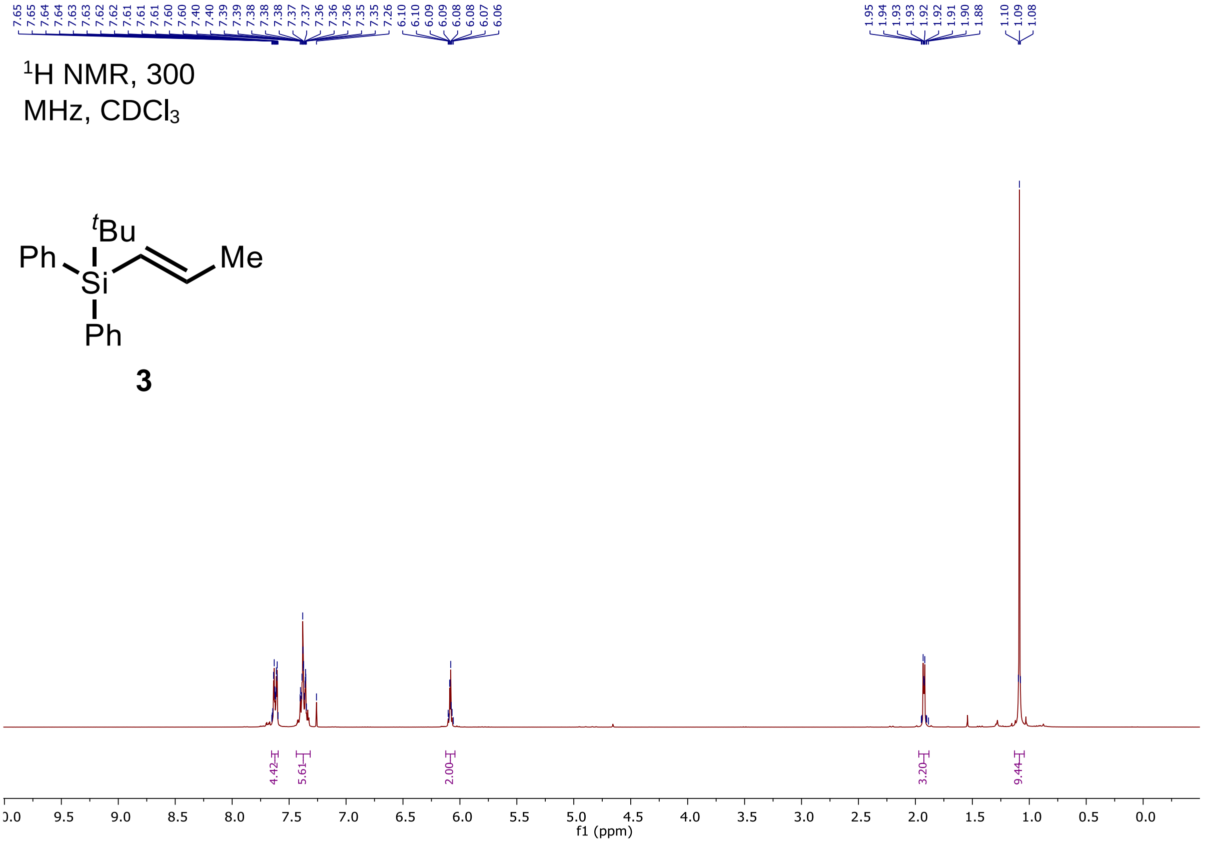

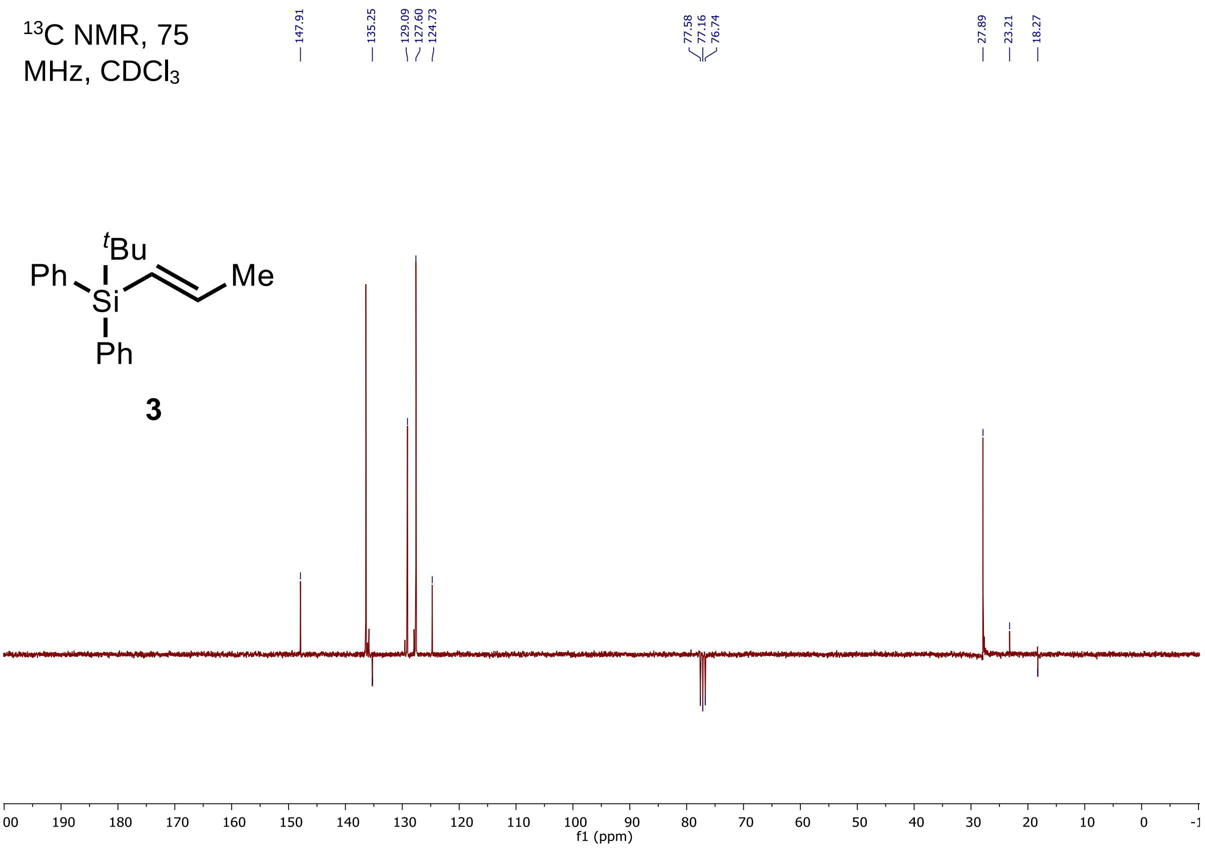

(*E*)-methyldiphenyl(prop-1-en-1-yl)silane (4)[18]

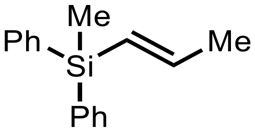

The title compound was prepared according to general procedure 2 using allyldiphenyl(methyl)silane (0.1 mmol). Purification by flash silica chromatography (eluent = 100% PE) gave the title compound as a colourless liquid (16.7 mg, 70%, *E*:*Z* = 93:7); R_f = 0.44 (eluent = 100% PE). NMR yield = 78% (*E*:*Z* = 93:7).

<u>Signals of the major isomer (*E*)-methyldiphenyl(prop-1-en-1-yl)silane:</u>

^{1}H NMR (500 MHz, Chloroform-*d*) δ 7.54 (dt, *J* = 6.3, 1.9 Hz, 4H), 7.42 – 7.33 (m, 6H), 6.18 (dq, *J* = 18.4, 6.1 Hz, 1H), 5.99 (dq, *J* = 18.4, 1.6 Hz, 1H), 1.90 (dd, *J* = 6.1, 1.6 Hz, 3H), 0.61 (s, 3H).

^{13}C NMR (126 MHz, Chloroform-*d*) δ 146.4, 137.2, 135.0, 129.3, 127.9, 127.2, 23.0, -3.6.

<u>Resolved signals of the minor isomer (*Z*)-methyldiphenyl(prop-1-en-1-yl)silane:</u>

^{1}H NMR (500 MHz, Chloroform-*d*) δ 6.70 (dq, *J* = 13.8, 6.9 Hz, 1H), 5.88 – 5.84 (m, 1H), 1.67 (dd, *J* = 6.8, 1.5 Hz, 3H), 0.68 (s, 3H).

^{1}H NMR, 500 MHz, $CDCl_3$

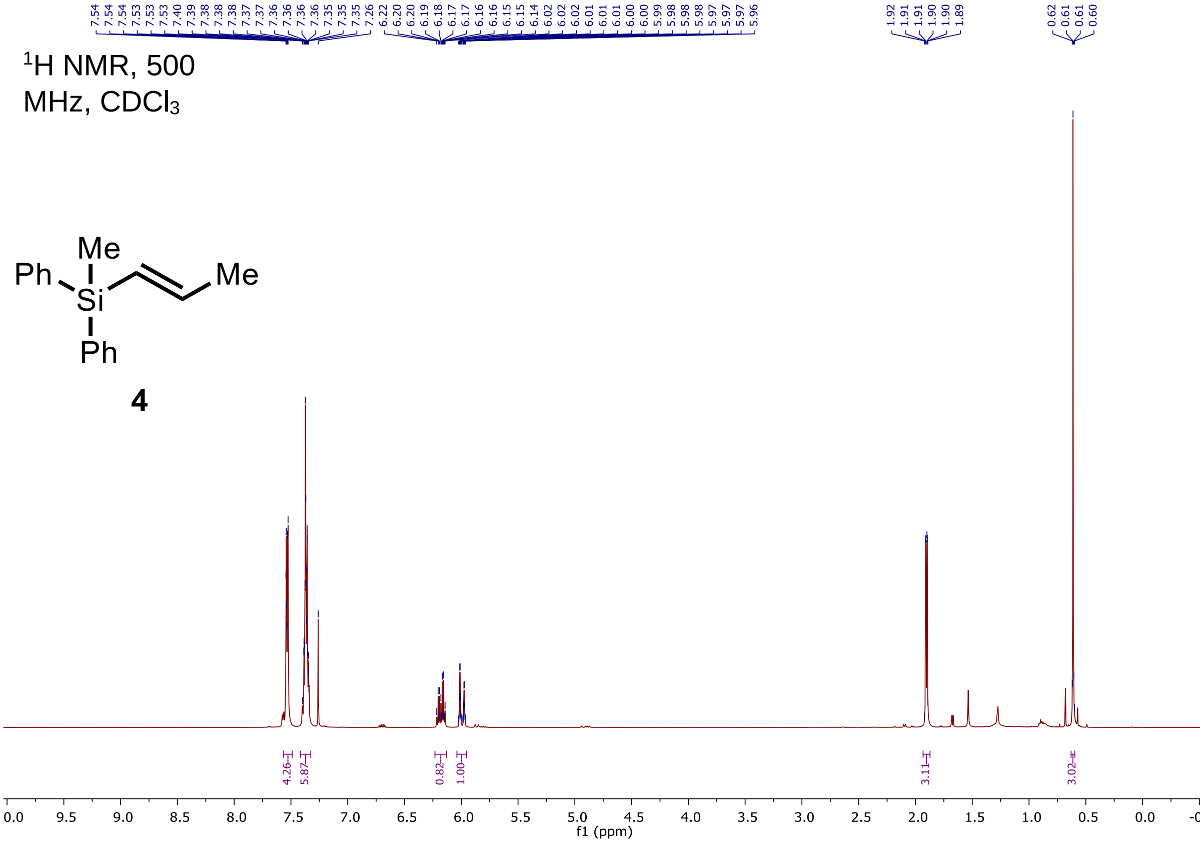

4

^{13}C NMR, 126 MHz, $CDCl_3$

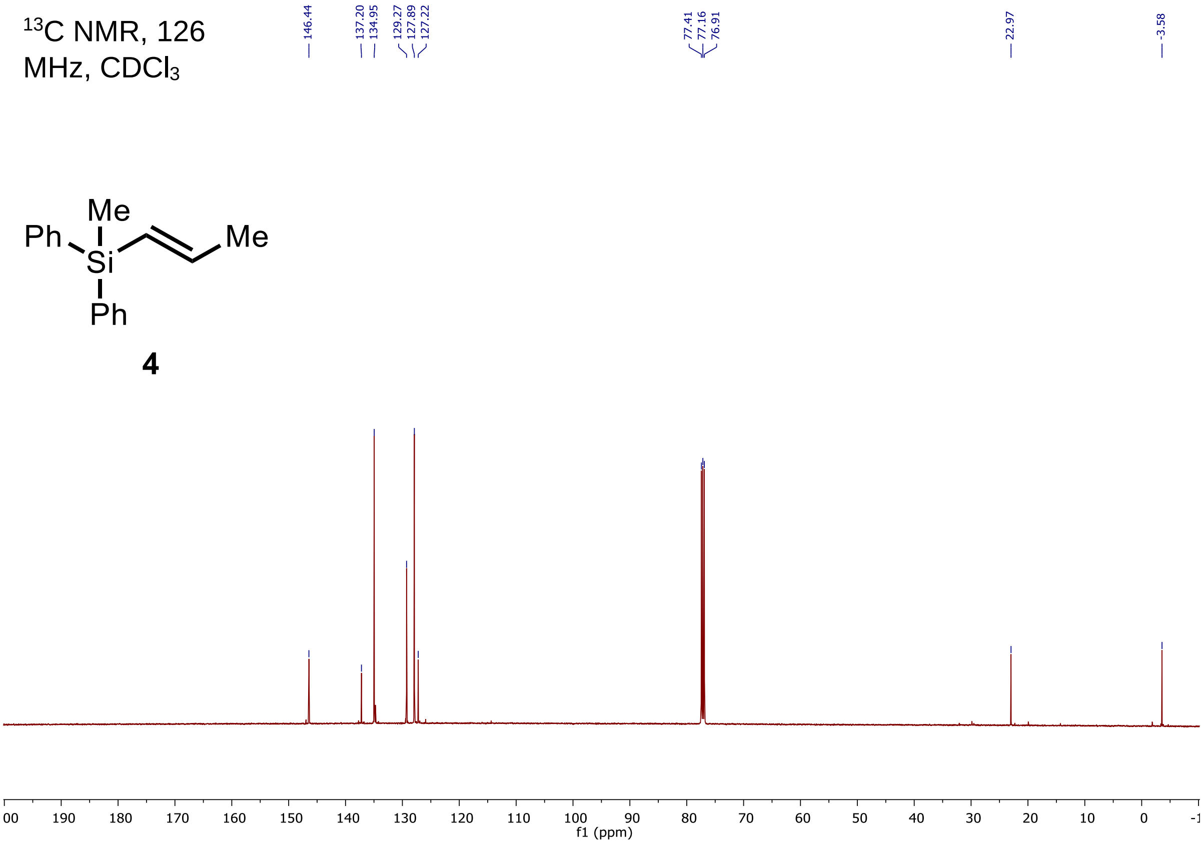

4

(*E*)-dimethyl(phenyl)(prop-1-en-1-yl)silane (5)[19]

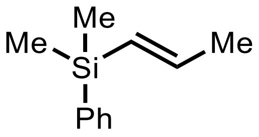

The title compound was prepared according to general procedure 2 using allyldimethylphenylsilane (0.1 mmol). Purification by flash silica chromatography (eluent = 100% PE) gave the title compound as a colourless liquid (9 mg, 50%, *E*:*Z* = 97:3); R_f = 0.59 (eluent = 100% PE). NMR yield = 80% (*E*:*Z* = 92:8).

<u>Signals of the major isomer (*E*)-dimethyl(phenyl)(prop-1-en-1-yl)silane:</u>

^{1}H NMR (300 MHz, Chloroform-*d*) δ 7.54 – 7.50 (m, 2H), 7.37 – 7.33 (m, 3H), 6.13 (dq, *J* = 18.4, 6.1 Hz, 1H), 5.78 (dq, *J* = 18.4, 1.6 Hz, 1H), 1.85 (dd, *J* = 6.1, 1.6 Hz, 3H), 0.31 (s, 6H).

^{13}C NMR (75 MHz, Chloroform-*d*) δ 144.2, 139.4, 133.9, 129.4, 128.9, 127.8, 22.8, -2.4.

<u>Resolved signals of the minor isomer (*Z*)-dimethyl(phenyl)(prop-1-en-1-yl)silane:</u>

^{1}H NMR (300 MHz, Chloroform-*d*) δ 6.54 (dd, *J* = 14.0, 6.9 Hz, 1H), 1.72 (dd, *J* = 6.8, 1.5 Hz, 3H), 0.46 (s, 6H).

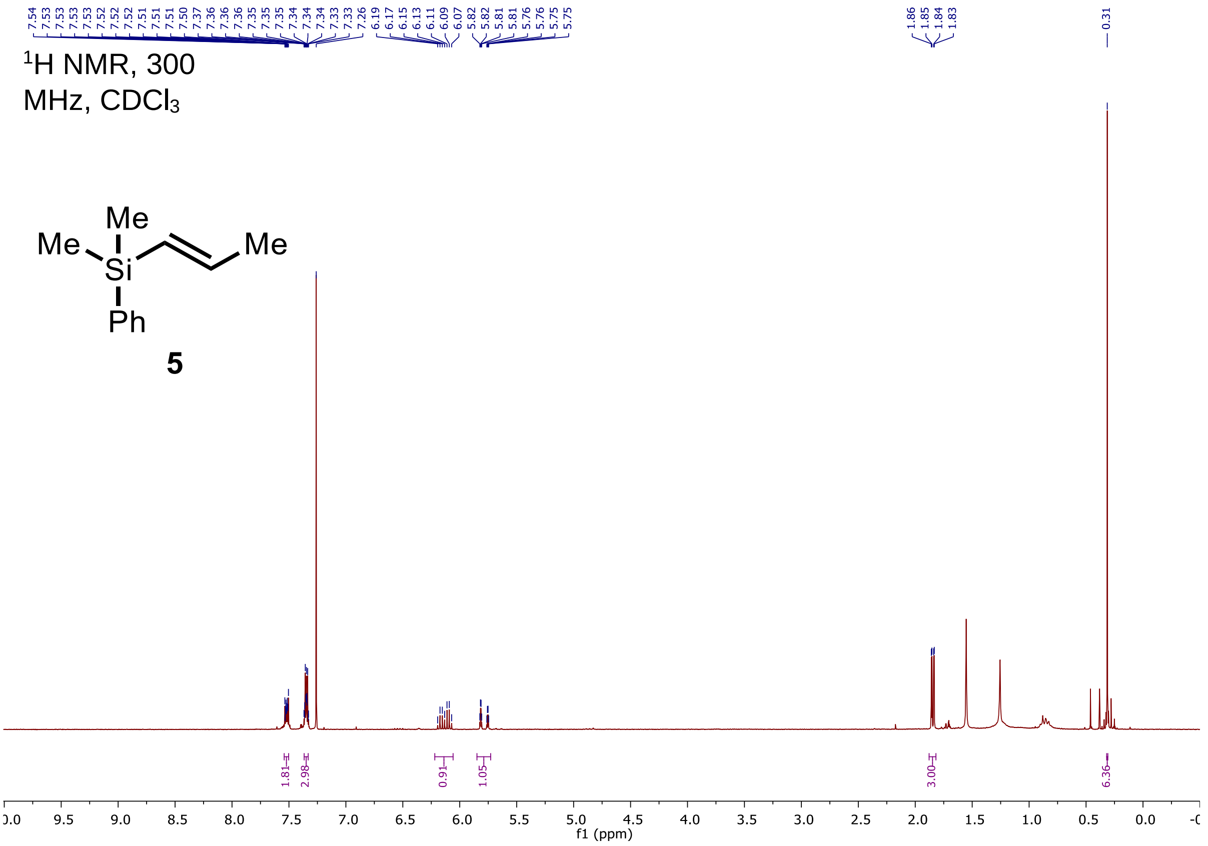

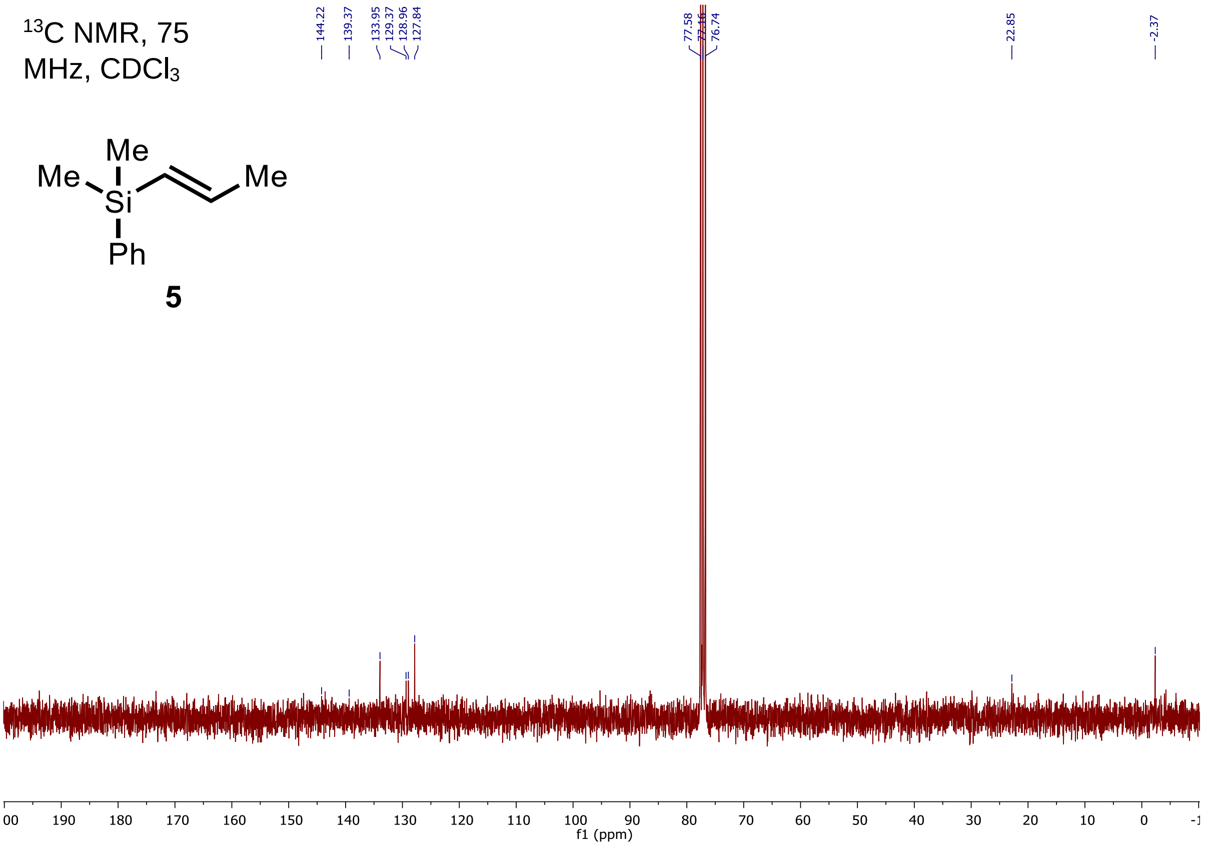

(*E*)-hexyl(methyl)(phenyl)(prop-1-en-1-yl)silane (6)

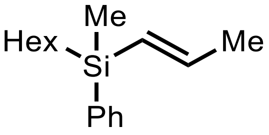

The title compound was prepared according to general procedure 2 using allylhexylmethylphenylsilane (0.1 mmol). Yield determined by crude ^{1}H NMR using 1,3,5-trimethylbenzene as internal standard: NMR yield = 50% (*E*:*Z* = 92:8).

<u>Resolved signals of the major isomer (*E*)-hexyl(methyl)(phenyl)(prop-1-en-1-yl)silane:</u>

^{1}H NMR (300 MHz, Chloroform-*d*) δ 6.57 (dq, *J* = 18.4, 6.1 Hz, 1H), 6.25 (dq, *J* = 18.4, 1.6 Hz, 1H), 2.27 (dd, *J* = 6.1, 1.6 Hz, 3H), 0.77 (s, 3H).

<u>Resolved signals of the minor isomer (*Z*)-hexyl(methyl)(phenyl)(prop-1-en-1-yl)silane:</u>

^{1}H NMR (300 MHz, Chloroform-*d*) δ 2.13 (dd, *J* = 6.7, 1.7 Hz, 3H), 0.59 (s, 3H).

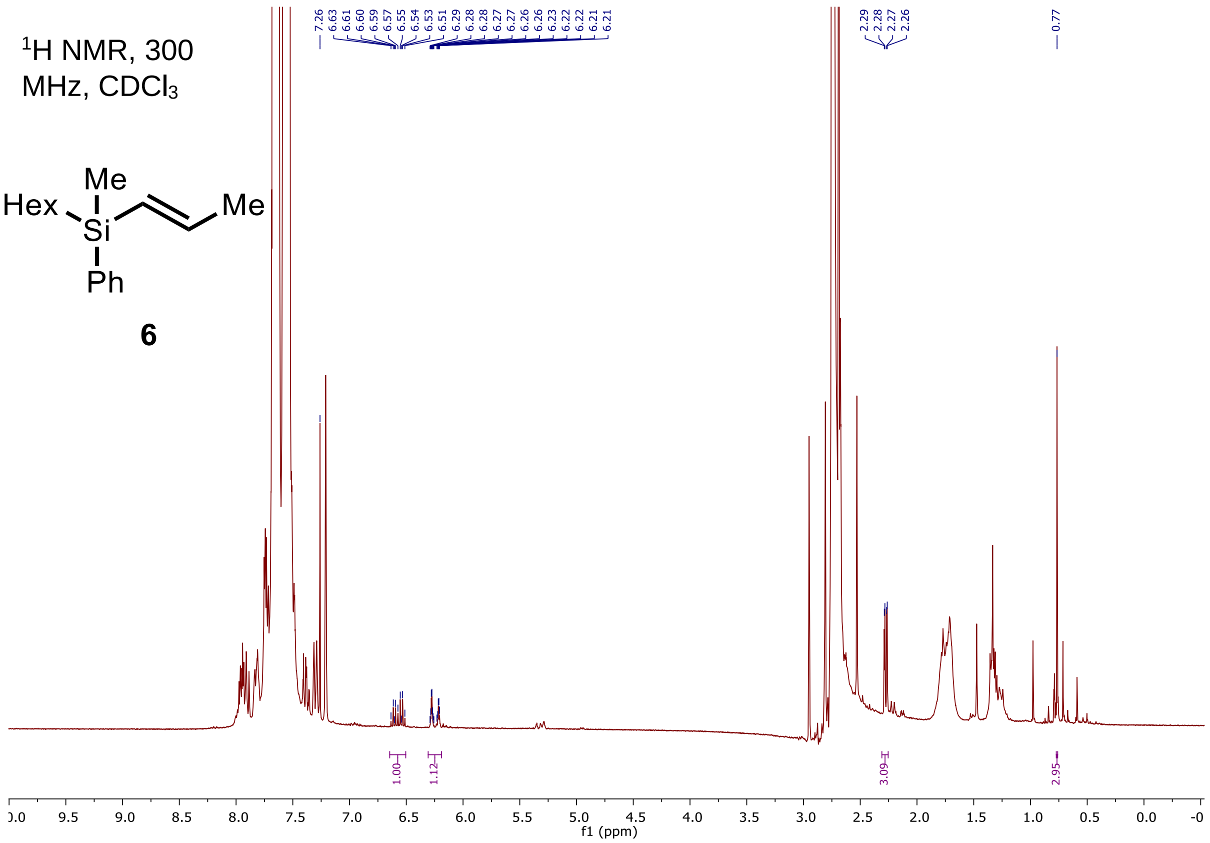

(*E*)-benzyldimethyl(prop-1-en-1-yl)silane (7)[20]

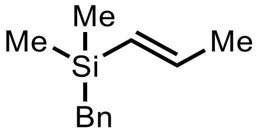

The title compound was prepared according to general procedure 2 using allylbenzyldimethylsilane (0.1 and 2 mmol). Purification of 0.1 mmol scale by flash silica chromatography (eluent = 100% PE) gave the title compound as a colourless liquid (9.5 mg, 50%, *E*:*Z* = 92:8); R_f = 0.44 (eluent = 100% PE). NMR yields = 87% (*E*:*Z* = 92:8) in 0.1 mmol and 80% (*E*:*Z* = 93:7) in 2 mmol scale. Note: product isolation by silica chromatography of 2 mmol scale was unsuccessful presumably due to hydrolysis.

<u>Signals of the major isomer (*E*)-benzyldimethyl(prop-1-en-1-yl)silane:</u>

^{1}H NMR (500 MHz, Chloroform-*d*) δ 7.20 (t, *J* = 7.6 Hz, 2H), 7.09 – 7.04 (m, 1H), 7.03 – 6.96 (m, 2H), 6.04 (dqd, *J* = 18.5, 6.1, 0.4 Hz, 1H), 5.63 (dqd, *J* = 18.4, 1.6, 0.4 Hz, 1H), 2.11 (s, 2H), 1.81 (ddd, *J* = 6.2, 1.7, 0.4 Hz, 3H), 0.02 (d, *J* = 0.5 Hz, 6H).

^{13}C NMR (126 MHz, Chloroform-*d*) δ 143.5, 140.4, 129.6, 128.4, 128.2, 124.0, 26.4, 22.8, -3.2.

<u>Resolved signals of the minor isomer (*Z*)-benzyldimethyl(prop-1-en-1-yl)silane:</u>

^{1}H NMR (500 MHz, Chloroform-*d*) δ 6.44 (dq, *J* = 13.7, 6.8 Hz, 1H), 5.47 (dq, *J* = 14.1, 1.5 Hz, 1H), 2.18 (d, *J* = 0.9 Hz, 2H), 1.71 (ddd, *J* = 6.8, 1.5, 0.4 Hz, 3H), 0.11 (d, *J* = 0.5 Hz, 6H).

^{1}H NMR, 500 MHz, $CDCl_3$

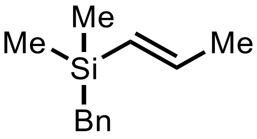

7

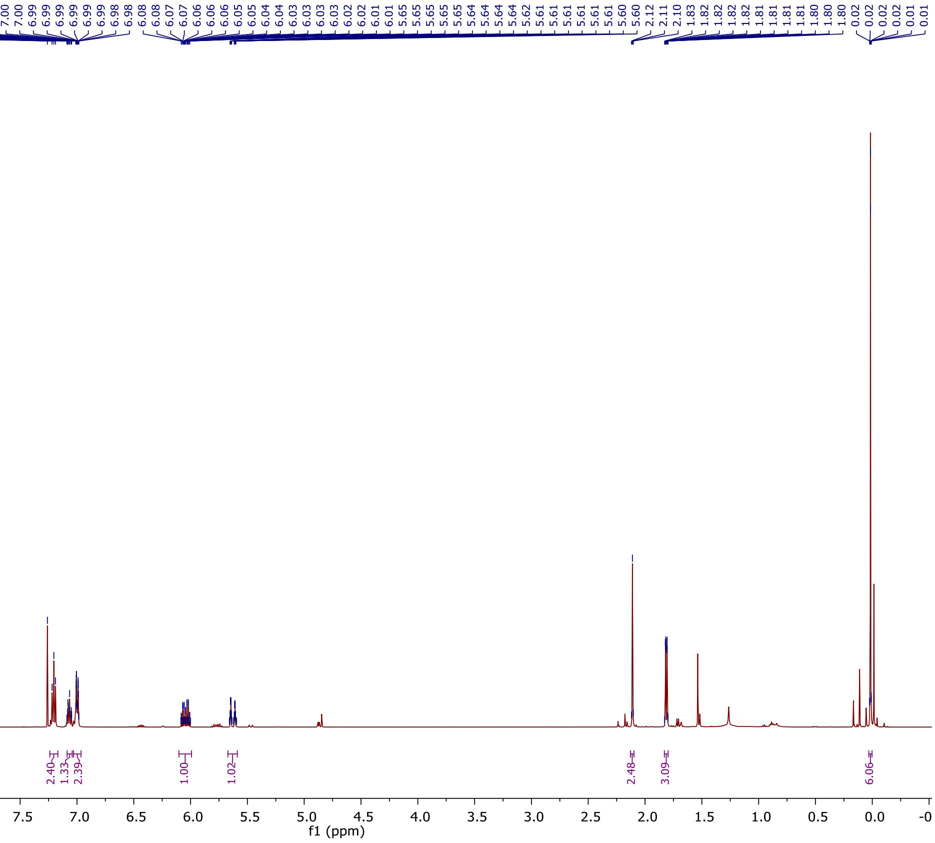

^{13}C NMR, 126 MHz, $CDCl_3$

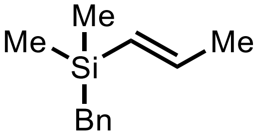

7

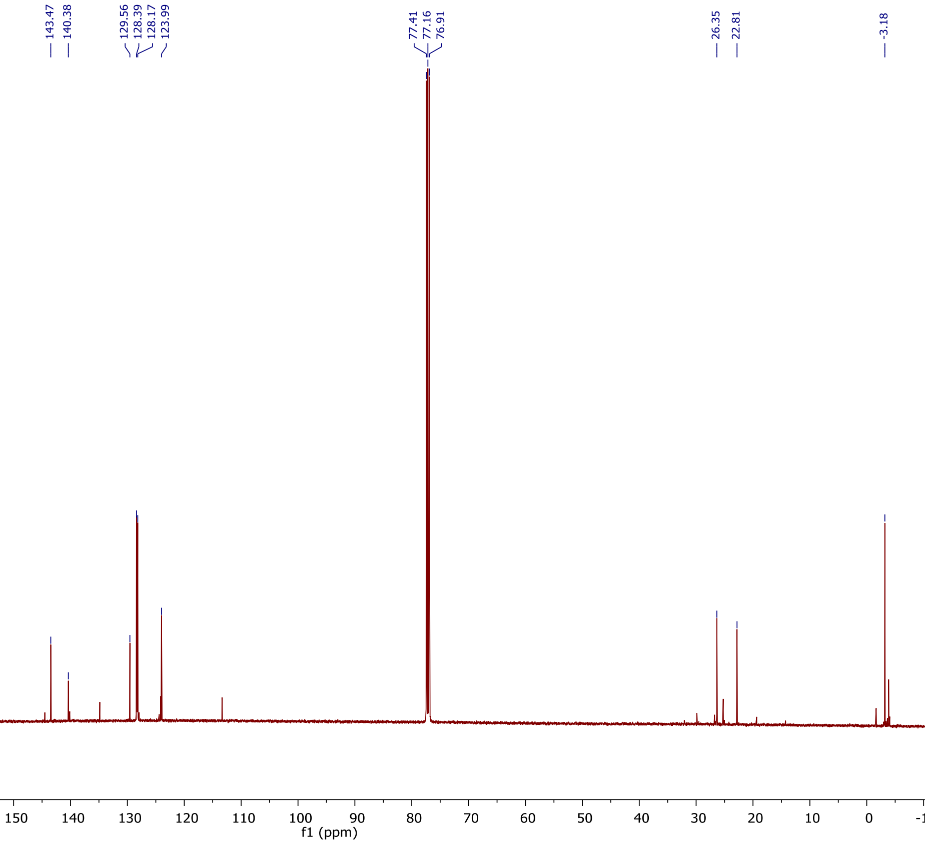

(*E*)-*tert*-butyldimethyl(prop-1-en-1-yl)silane (8)[20]

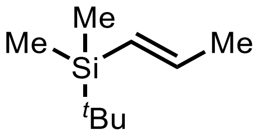

The title compound was prepared according to general procedure 2 using allyl*tert*butyldimethylsilane (0.1 mmol). Yield determined by crude ^{1}H NMR using 1,3,5-trimethylbenzene as internal standard: 49% (*E*:*Z* = 92:8).

Resolved signals of the major isomer (*E*)-*tert*-butyldimethyl(prop-1-en-1-yl)benzene:

^{1}H NMR (500 MHz, Chloroform-*d*) δ 6.55 – 6.44 (m, 1H), 6.11 (dq, *J* = 18.5, 1.7 Hz, 1H), 2.25 (dt, *J* = 6.1, 1.5 Hz, 3H), 0.47 (d, *J* = 1.2 Hz, 6H).

Resolved signals of the minor isomer (*Z*)-*tert*-butyldimethyl(prop-1-en-1-yl)benzene:

^{1}H NMR (500 MHz, Chloroform-*d*) δ 6.01 – 5.94 (m, 1H), 2.20 (dd, *J* = 6.9, 1.6 Hz, 3H), 0.58 (s, 6H).

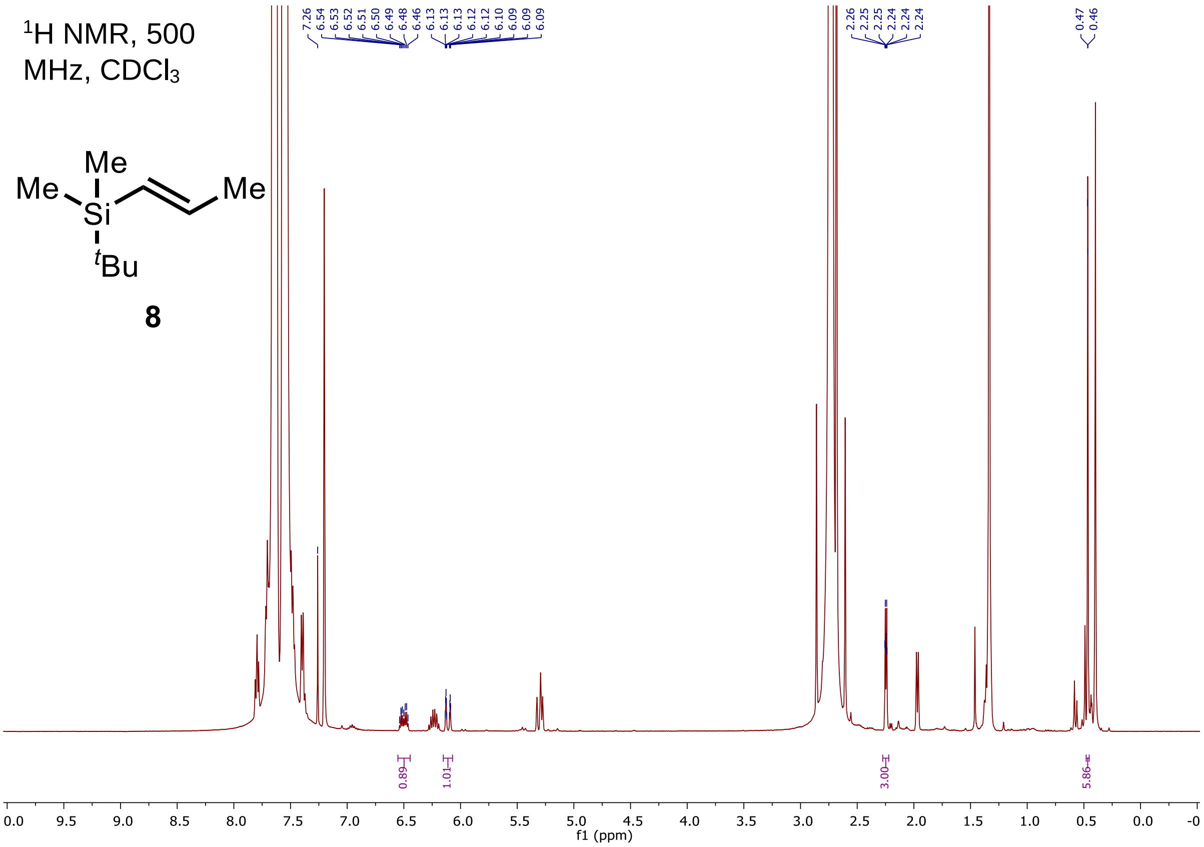

(*E*)-decyldimethyl(prop-1-en-1-yl)silane (9)

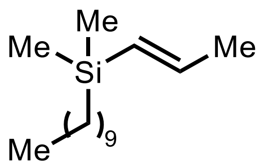

The title compound was prepared according to general procedure 2 using allyl(decyl)dimethylsilane (0.1 mmol). Yield determined by crude ^{1}H NMR using 1,3,5-trimethylbenzene as internal standard: 40% (*E*:*Z* = 95:5).

Resolved signals of the major isomer (*E*)-decyldimethyl(prop-1-en-1-yl)silane:

^{1}H NMR (300 MHz, Chloroform-*d*) δ 6.46 (dq, *J* = 18.4, 6.1 Hz, 1H), 6.06 (dq, *J* = 18.4, 1.6 Hz, 1H), 2.20 (dd, *J* = 6.1, 1.6 Hz, 3H), 0.45 (s, 6H).

Resolved signals of the minor isomer (*Z*)-decyldimethyl(prop-1-en-1-yl)silane:

^{1}H NMR (300 MHz, Chloroform-*d*) δ 6.82 (dd, *J* = 13.9, 6.8 Hz, 1H), 5.92 (dd, *J* = 14.0, 1.5 Hz, 1H), 2.17 (dd, *J* = 6.8, 1.5 Hz, 3H), 0.56 (s, 6H).

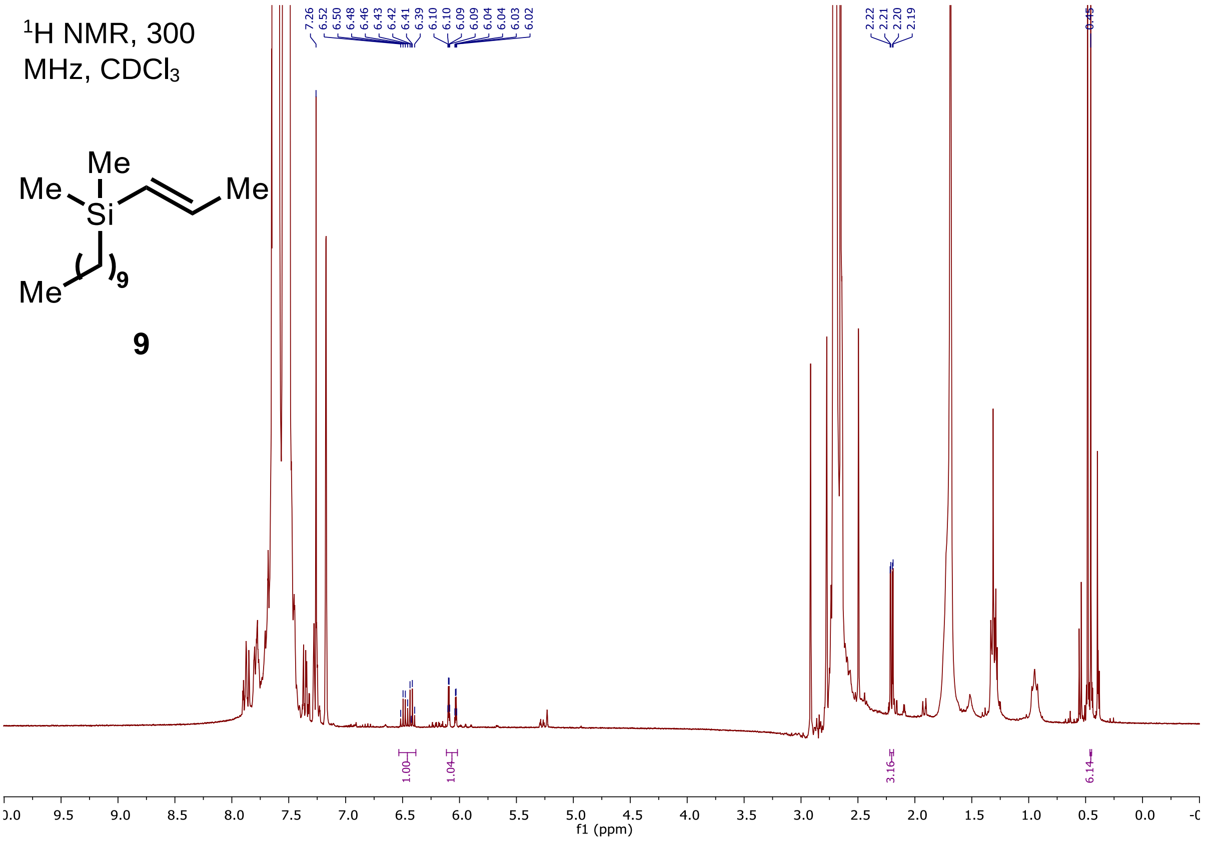

(*E*)-dimethyl(3-phenylpropyl)(prop-1-en-1-yl)silane (10)

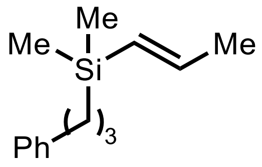

The title compound was prepared according to general procedure 2 using allyl(3-phenylpropyl)dimethylsilane (0.1 mmol). Yield determined by crude ^{1}H NMR using 1,3,5-trimethylbenzene as internal standard: 70% (*E*:*Z* = 94:6).

Resolved signals of the major isomer (*E*)-dimethyl(3-phenylpropyl)(prop-1-en-1-yl)benzene:

^{1}H NMR (300 MHz, Chloroform-*d*) δ 6.50 (dq, *J* = 18.4, 6.1 Hz, 1H), 6.11 (dq, *J* = 18.4, 1.6 Hz, 1H), 2.26 (dd, *J* = 6.1, 1.6 Hz, 3H), 1.09 – 1.02 (m, 2H), 0.52 (s, 6H).

Resolved signals of the minor isomer (*Z*)-dimethyl(3-phenylpropyl)(prop-1-en-1-yl)benzene:

^{1}H NMR (300 MHz, Chloroform-*d*) δ 6.87 (dd, *J* = 14.0, 6.8 Hz, 1H), 5.97 (dd, *J* = 14.0, 1.5 Hz, 1H), 2.21 (dd, *J* = 6.8, 1.5 Hz, 3H), 0.60 (s, 6H).

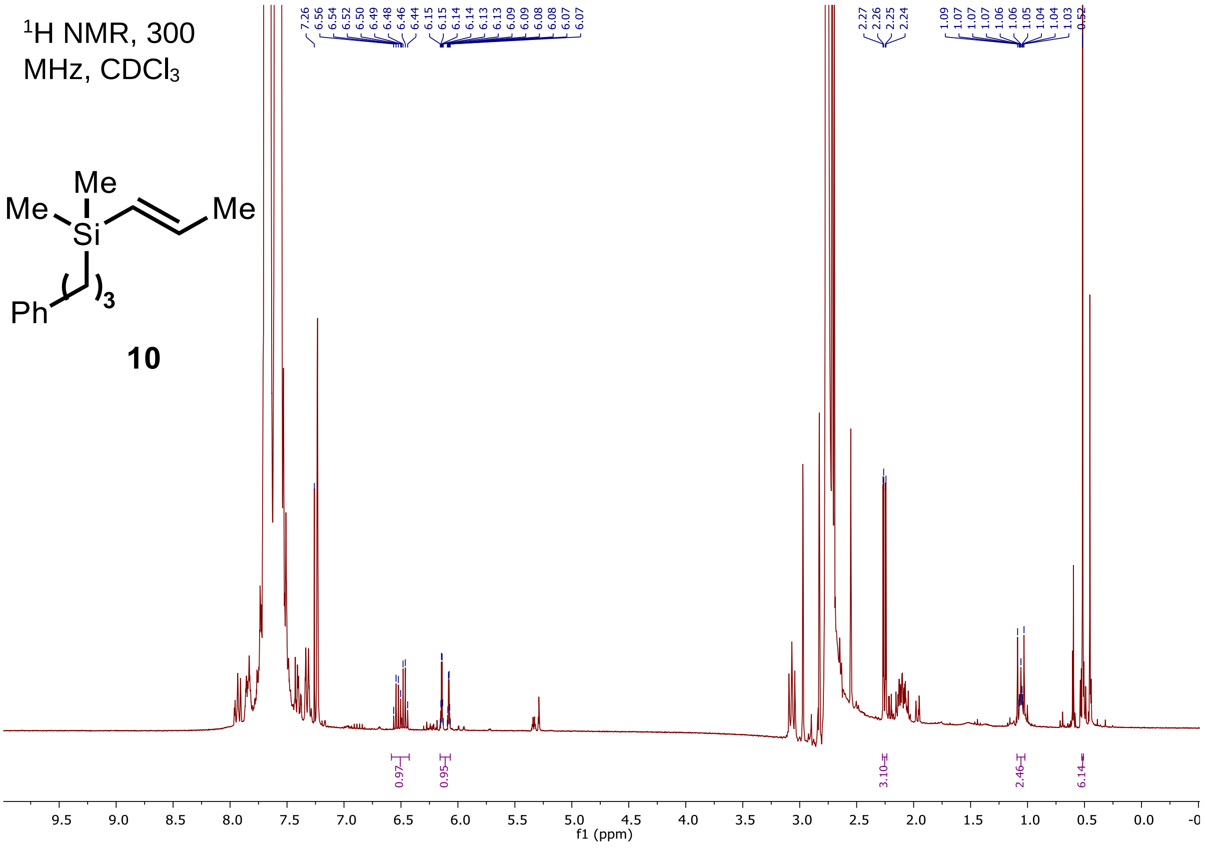

(*E*)-tributyl(prop-1-en-1-yl)silane (11)

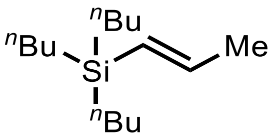

The title compound was prepared according to general procedure 2 using allyltributylsilane (0.1 mmol). Yield determined by crude ^{1}H NMR using 1,3,5-trimethylbenzene as internal standard: 76% (*E*:*Z* = 90:10).

Resolved signals of the major isomer (*E*)-tributyl(prop-1-en-1-yl)silane:

^{1}H NMR (300 MHz, Chloroform-*d*) δ 6.42 (dq, *J* = 18.4, 6.1 Hz, 1H), 5.98 (dq, *J* = 18.5, 1.6 Hz, 1H), 2.17 (dd, *J* = 6.1, 1.6 Hz, 3H).

Resolved signals of the minor isomer (*Z*)-tributyl(prop-1-en-1-yl)silane:

^{1}H NMR (300 MHz, Chloroform-*d*) δ 6.80 (dt, *J* = 13.7, 6.9 Hz, 1H), 5.84 (dd, *J* = 14.1, 1.5 Hz, 1H), 2.13 (dd, *J* = 6.8, 1.5 Hz, 3H).

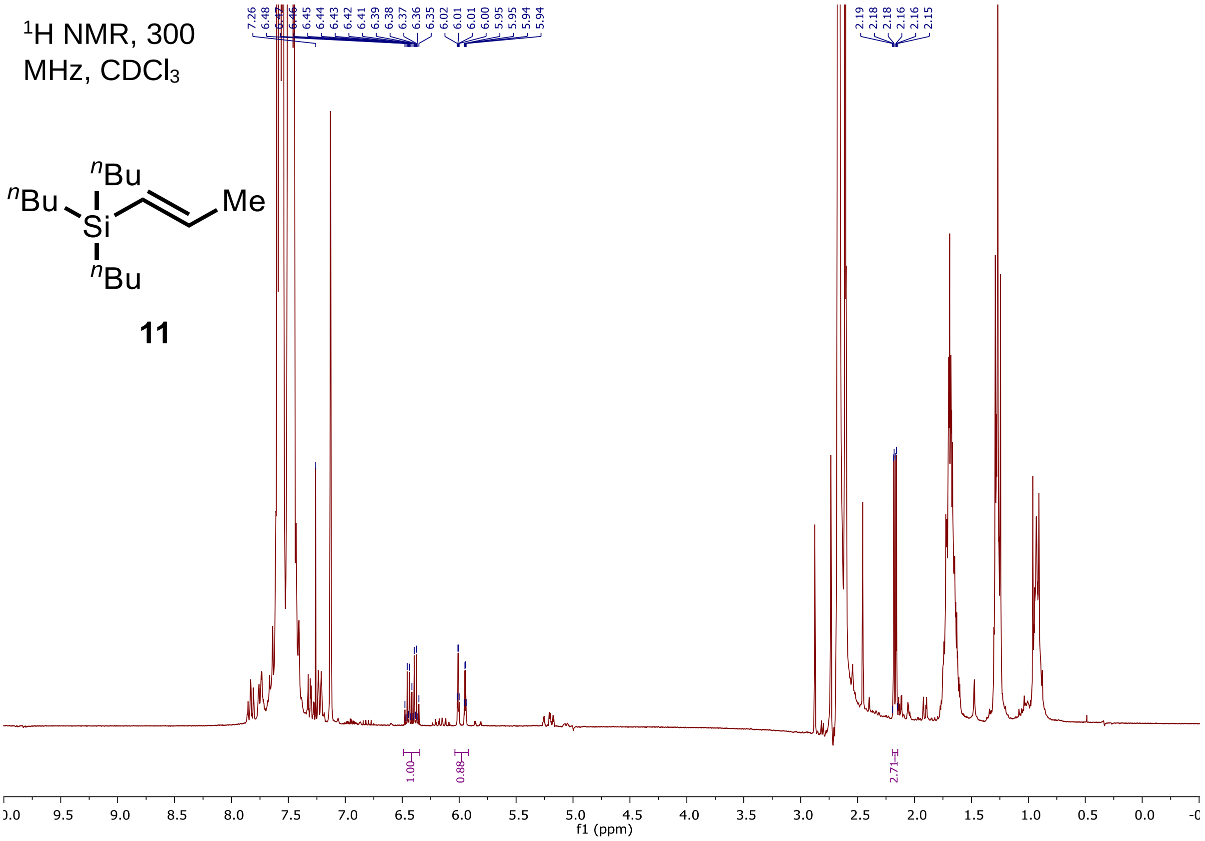

(*E*)-triisopropyl(prop-1-en-1-yl)silane (12)[20]

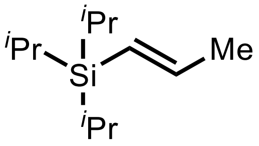

The title compound was prepared according to general procedure 2 using allyltriisopropylsilane (0.1 mmol). Yield determined by crude ^{1}H NMR using 1,3,5-trimethylbenzene as internal standard: 78% (*E*:*Z* = 98:2).

<u>Resolved signals of the major isomer (*E*)-triisopropyl(prop-1-en-1-yl)silane:</u>

^{1}H NMR (500 MHz, Chloroform-*d*) δ 6.52 (dq, *J* = 18.5, 6.1 Hz, 1H), 5.98 (dq, *J* = 18.7, 1.7 Hz, 1H), 2.25 (dd, *J* = 6.1, 1.7 Hz, 3H).

<u>Resolved signals of the minor isomer (*Z*)-triisopropyl(prop-1-en-1-yl)silane:</u>

^{1}H NMR (500 MHz, Chloroform-*d*) δ 5.86 (dq, *J* = 14.4, 1.6 Hz, 1H).

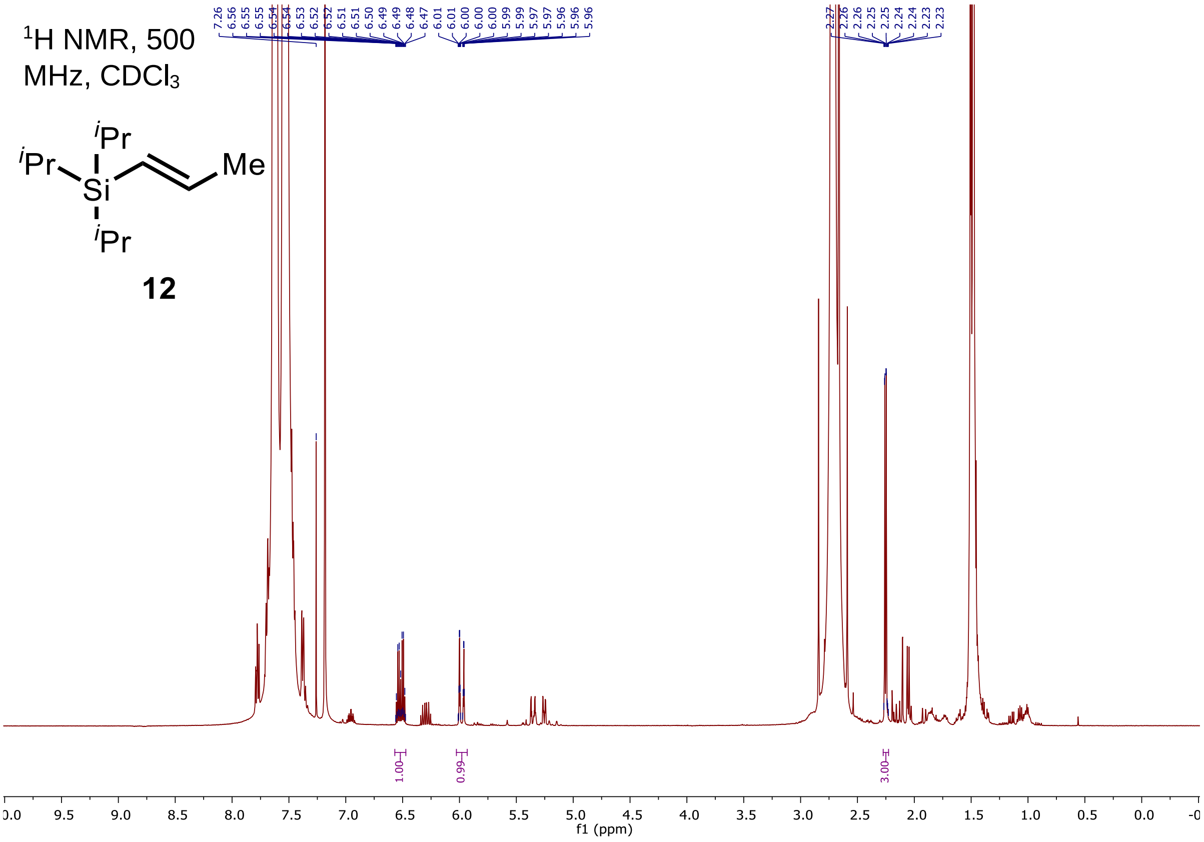

(*E*)-trimethyl(prop-1-en-1-yl)silane (13)[21]

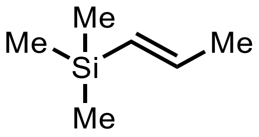

The title compound was prepared according to general procedure 2 using allyltrimethylsilane (0.1 mmol). Yield determined by crude ^{1}H NMR using 1,3,5-trimethylbenzene as internal standard: 54% (*E*:*Z* = 95:5).

Signals of the major isomer (*E*)-trimethyl(prop-1-en-1-yl)silane:

^{1}H NMR (300 MHz, Chloroform-*d*) δ 6.60 (dq, *J* = 18.5, 6.1 Hz, 1H), 6.23 (dtd, *J* = 18.3, 1.8, 1.3 Hz, 1H), 2.33 (dd, *J* = 6.1, 1.6 Hz, 3H), 0.63 (d, *J* = 0.6 Hz, 9H).

Signals of the minor isomer (*Z*)-trimethyl(prop-1-en-1-yl)silane:

^{1}H NMR (300 MHz, Chloroform-*d*) δ 6.99 – 6.88 (m, 1H), 6.13 – 6.06 (m, 1H), 2.32 – 2.29 (m, 3H), 0.71 (s, 9H).

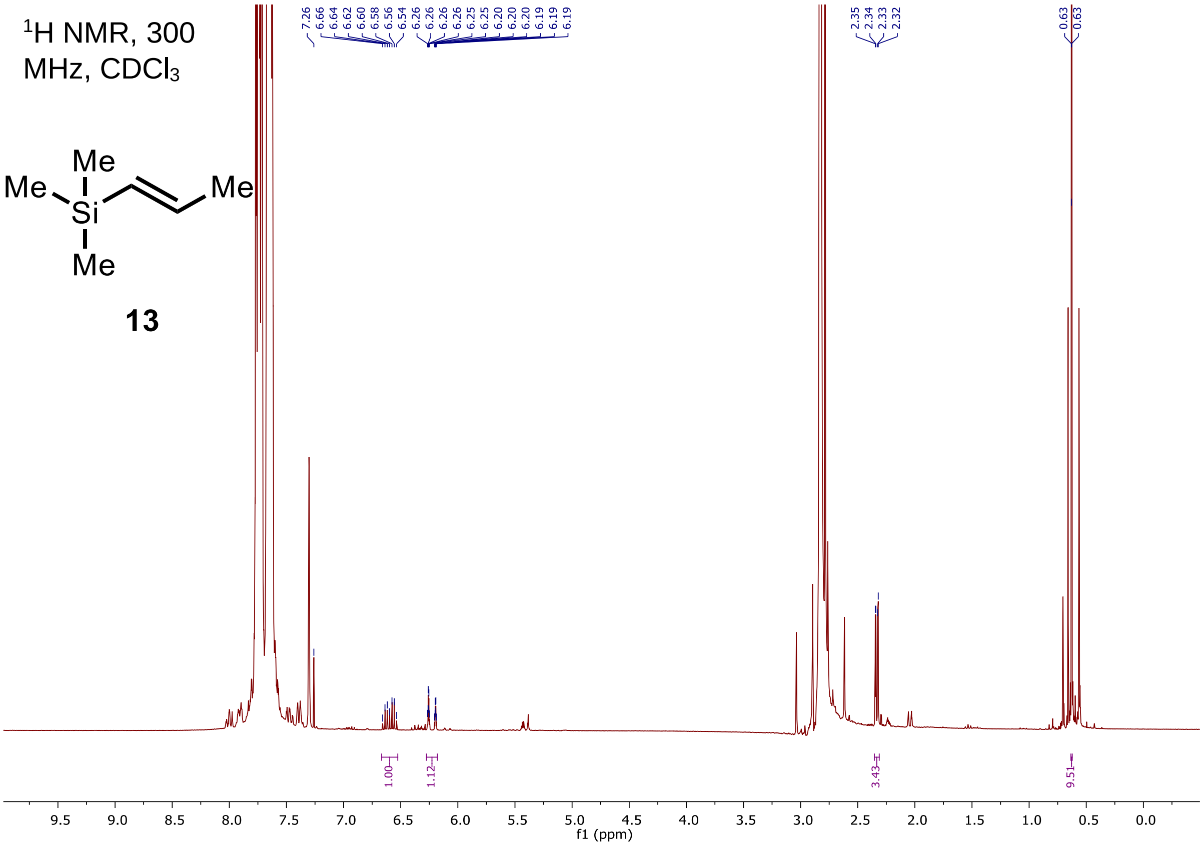

(*E*)-triphenyl(prop-1-en-1-yl)germane (14)[22]

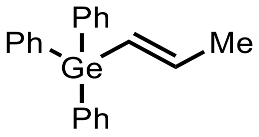

The title compound was prepared according to general procedure 2 using allyltriphenylgermane (0.1 mmol). Yield determined by crude ^{1}H NMR using 1,3,5-trimethylbenzene as internal standard: 58% (*E*:*Z* = 93:7).

<u>Resolved signals of the major isomer (*E*)-triphenyl(prop-1-en-1-yl)germane:</u>

^{1}H NMR (300 MHz, Chloroform-*d*) δ 6.61 – 6.37 (m, 2H), 2.25 – 2.21 (m, 3H).

<u>Resolved signals of the minor isomer (*Z*)-triphenyl(prop-1-en-1-yl)germane:</u>

^{1}H NMR (300 MHz, Chloroform-*d*) δ 1.98 (dd, *J* = 6.8, 1.5 Hz, 3H).

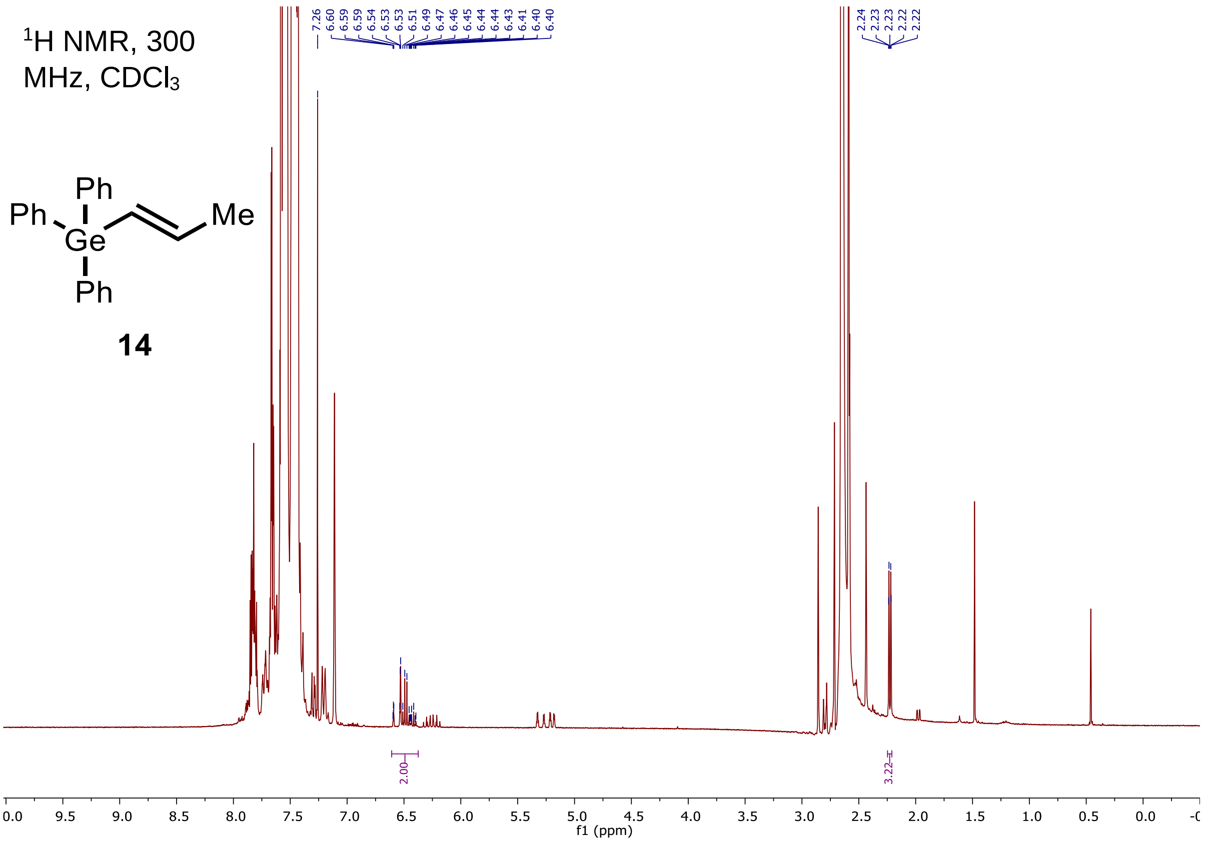

(*E*)-triethyl(prop-1-en-1-yl)germane (15)

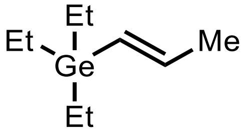

The title compound was prepared according to general procedure 2 using allyltriethylgermane (0.1 mmol). Yield determined by crude ^{1}H NMR using 1,3,5-trimethylbenzene as internal standard: 45% (*E*:*Z* = 89:11).

Signals of the major isomer (*E*)-triethyl(prop-1-en-1-yl)germane:

^{1}H NMR (300 MHz, Chloroform-*d*) δ 6.47 (dq, *J* = 18.1, 6.0 Hz, 1H), 6.26 (dq, *J* = 18.2, 1.5 Hz, 1H), 2.32 (dd, *J* = 6.0, 1.5 Hz, 3H), 1.56 (dd, *J* = 4.1, 0.8 Hz, 9H), 1.39 – 1.17 (m, 6H).

Resolved signals of the minor isomer (*Z*)-triethyl(prop-1-en-1-yl)germane:

^{1}H NMR (300 MHz, Chloroform-*d*) δ 6.17 – 6.09 (m, 1H), 2.25 (dd, *J* = 6.7, 1.5 Hz, 3H).

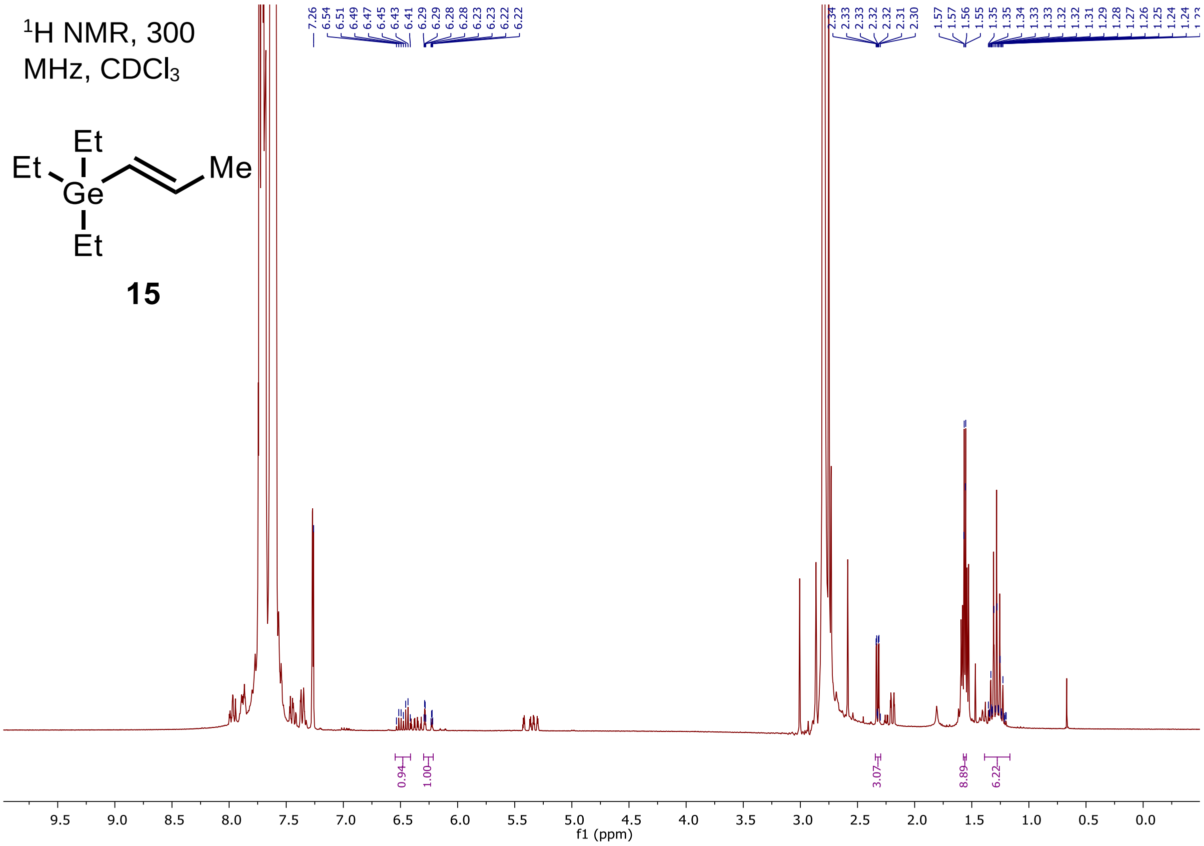

2.3.3. General procedure 3

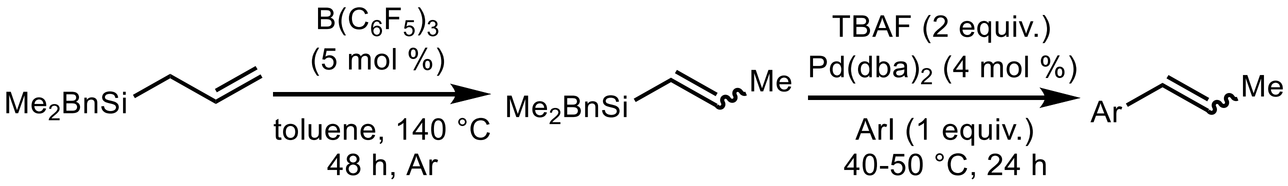

In the glovebox under Ar, an oven-dried 10 mL microwave vial equipped with a magnetic stirrer bar was charged with $B(C_6F_5)_3$ (5 mol %), allylbenzyldimethylsilane (0.1 mmol), and toluene (0.4 mL). The vial was sealed with an aluminium crimp cap and stirred at 140 ˚C for 48 h. It was cooled to rt, TBAF (2 equiv.) was added by syringe and stirred at rt for 10 min. The aluminium cap was removed, ArI (1 equiv.) and $Pd(dba)_2$ (4 mol %) were added. The vial then was sealed with an aluminium crimp cap and stirred at 40-50 ˚C for 24 h. it was cooled to rt, wet toluene was added. Then 1,3,5-trimethylbenzene (0.1 mmol) was added and stirred for 5 min and the yield was analysed using ^1H NMR. Purification was done by flask silica chromatography with the stated eluent.

(*E*)-1-(phenoxymethyl)-4-(prop-1-en-1-yl)benzene (16)

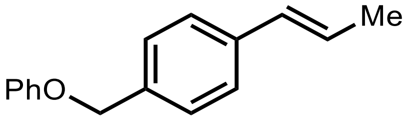

The title compound was prepared according to general procedure 3 using 1-Iodo-4-(phenoxymethyl)benzene (0.1 mmol). Purification by flask silica chromatography (eluent = 100% PE) gave the title compound as off-white solid (19 mg, 85%, *E*:*Z* = 90:10); mp 97–98 ˚C; Rf: 0.33 (eluent = 100% PE). NMR yield = 91% (*E*:*Z* = 87:13).

Signals of the major isomer (*E*)-1-(phenoxymethyl)-4-(prop-1-en-1-yl)benzene:

^{1}H NMR (300 MHz, Chloroform-*d*) δ 7.40 – 7.27 (m, 5H), 7.30 – 7.26 (m, 2H), 7.00 – 6.95 (m, 2H), 6.41 (dq, *J* = 15.7, 1.5 Hz, 1H), 6.25 (dq, *J* = 15.7, 6.4 Hz, 1H), 5.04 (s, 2H), 1.89 (dd, *J* = 6.4, 1.5 Hz, 3H).

^{13}C NMR (75 MHz, Chloroform-*d*) δ 158.9, 137.9, 135.6, 130.8, 129.6, 127.9, 126.2, 126.1, 121.0, 115.0, 69.9, 18.7.

HRMS (CI) calculated $[C_{16}H_{17}O]^+$ $(M+H)^+$: m/z 225.1274, found 225.1274.

IR (film, ν_{max} / cm^{-1}) 2961, 2930, 2913, 2878, 2851, 1595, 1584, 1495, 1381, 1240, 1169, 1013, 968, 689.

Resolved signals of the minor isomer (*Z*)-1-(phenoxymethyl)-4-(prop-1-en-1-yl)benzene:

^{1}H NMR (300 MHz, Chloroform-*d*) δ 7.74 – 7.69 (m, 3H), 7.22 – 7.17 (m, 2H), 5.81 (dq, *J* = 11.6, 7.2 Hz, 1H), 5.01 (s, 3H).

^{1}H NMR, 300 MHz, $CDCl_3$

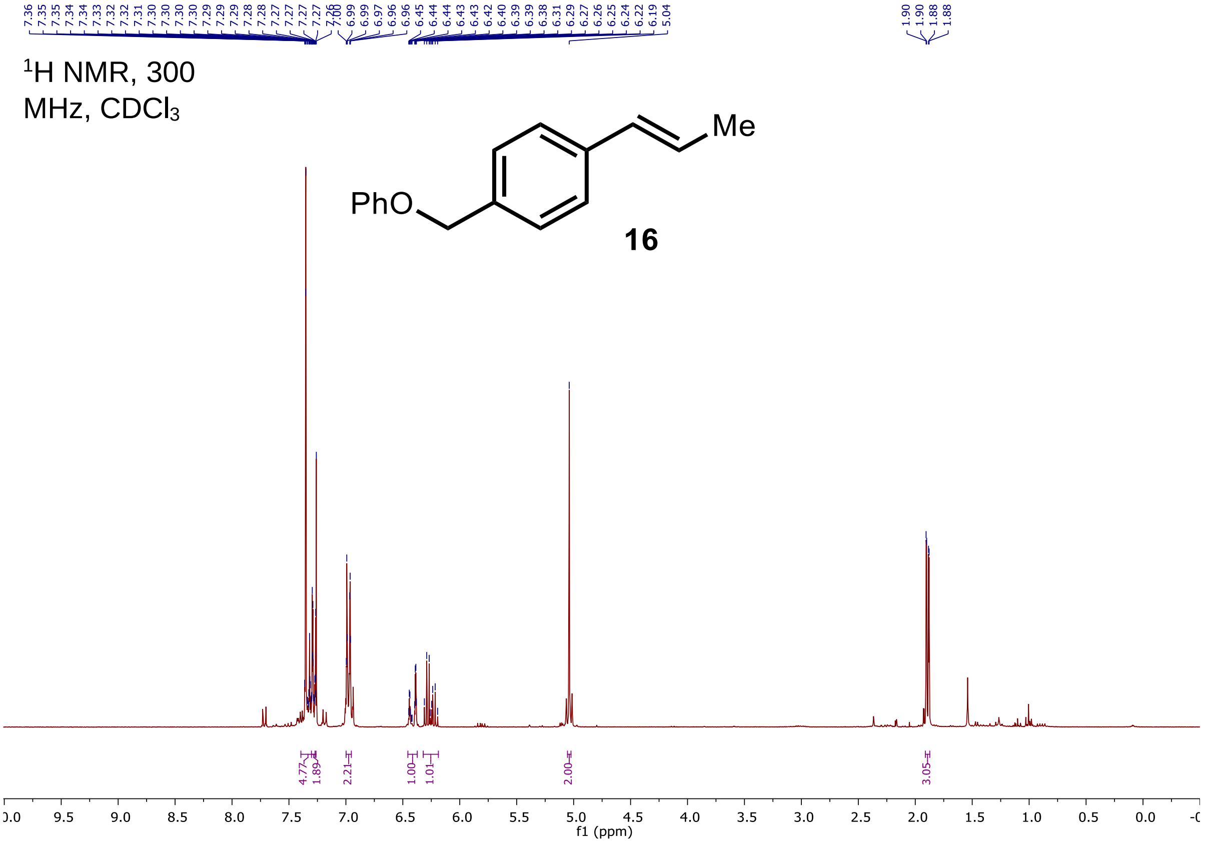

^{13}C NMR, 75 MHz, $CDCl_3$

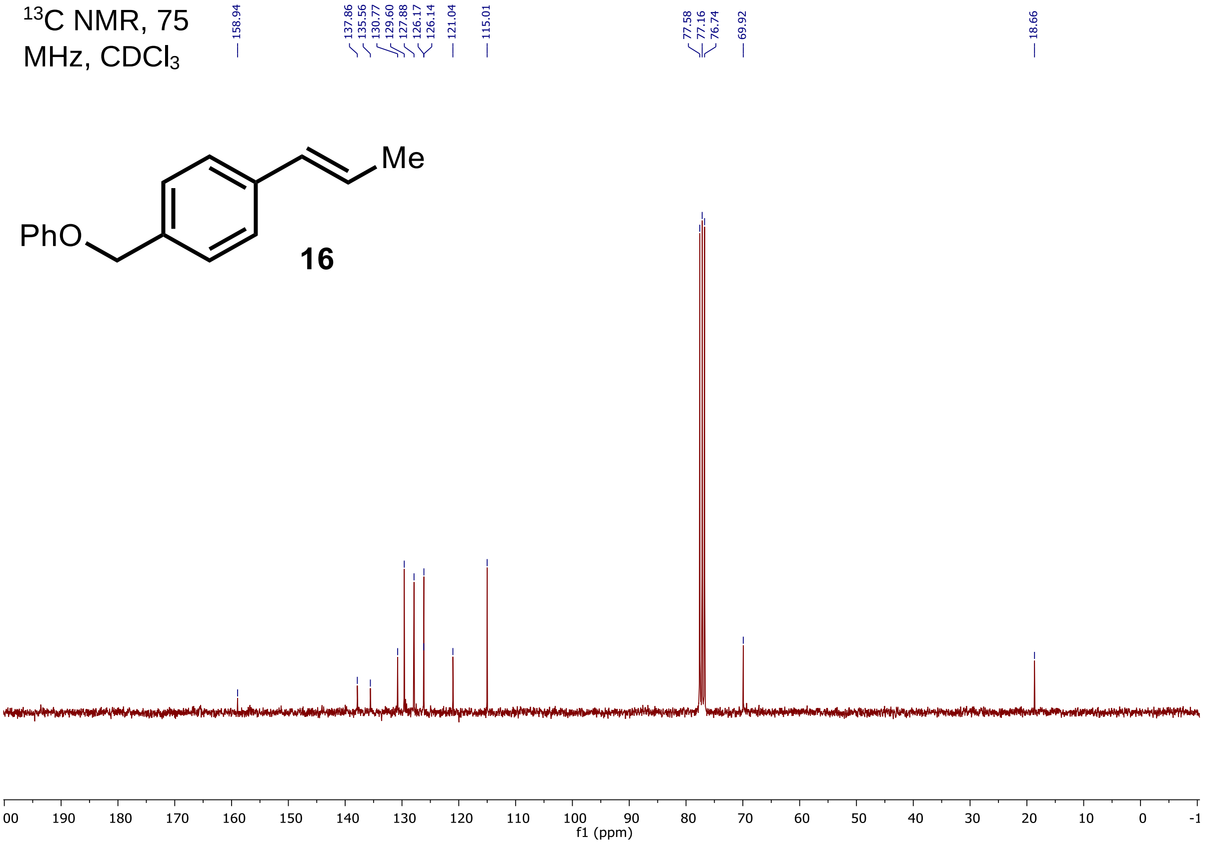

(*E*)-1-(benzyloxy)-4-(prop-1-en-1-yl)benzene (17)[23]

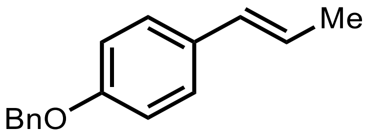

The title compound was prepared according to general procedure 3 using 1-(benzyloxy)-4-iodobenzene (0.1 mmol). Yield determined by crude ^{1}H NMR using 1,3,5-trimethylbenzene as internal standard: NMR yield = 92% (*E*:*Z* = 75:25).

<u>Resolved signals of the major isomer (*E*)-1-(benzyloxy)-4-(prop-1-en-1-yl)benzene:</u>

^{1}H NMR (400 MHz, Chloroform-*d*) δ 6.60 (dd, *J* = 15.9, 1.8 Hz, 1H), 6.33 (dq, *J* = 15.6, 6.5 Hz, 1H), 5.23 (s, 2H).

<u>Resolved signals of the minor isomer (*Z*)-1-(benzyloxy)-4-(prop-1-en-1-yl)benzene:</u>

^{1}H NMR (400 MHz, Chloroform-*d*) δ 5.96 (dd, *J* = 11.6, 7.1 Hz, 1H), 5.16 (s, 2H).

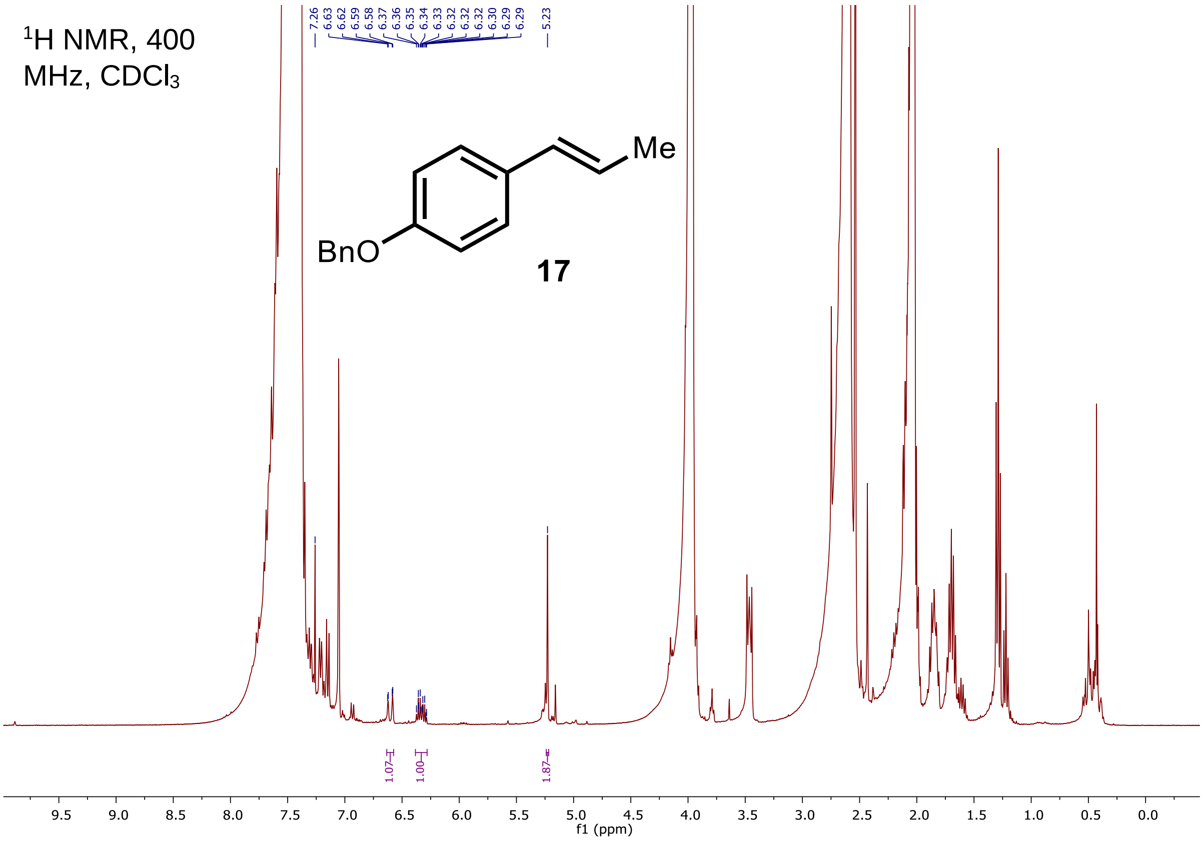

(*E*)-*N*,*N*-dibutyl-4-(prop-1-en-1-yl)aniline (18)

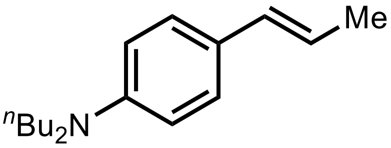

The title compound was prepared according to general procedure 3 using *N*,*N*-dibutyl-4-iodoaniline (0.1 mmol). Yield determined by crude ^{1}H NMR using 1,3,5-trimethylbenzene as internal standard: NMR yield = 59% (*E*:*Z* = 97:3).

Resolved signals of the major isomer (*E*)-*N*.*N*-dibutyl-4-(prop-1-en-1-yl)aniline:

^{1}H NMR (300 MHz, Chloroform-*d*) δ 6.83 – 6.79 (m, 2H), 6.54 (dt, *J* = 15.7, 2.0 Hz, 1H), 6.21 (dq, *J* = 15.7, 6.6 Hz, 1H).

Resolved signals of the minor isomer (*Z*)-*N*.*N*-dibutyl-4-(prop-1-en-1-yl)aniline:

^{1}H NMR (300 MHz, Chloroform-*d*) δ 5.81 (dd, *J* = 11.6, 7.2 Hz, 1H).

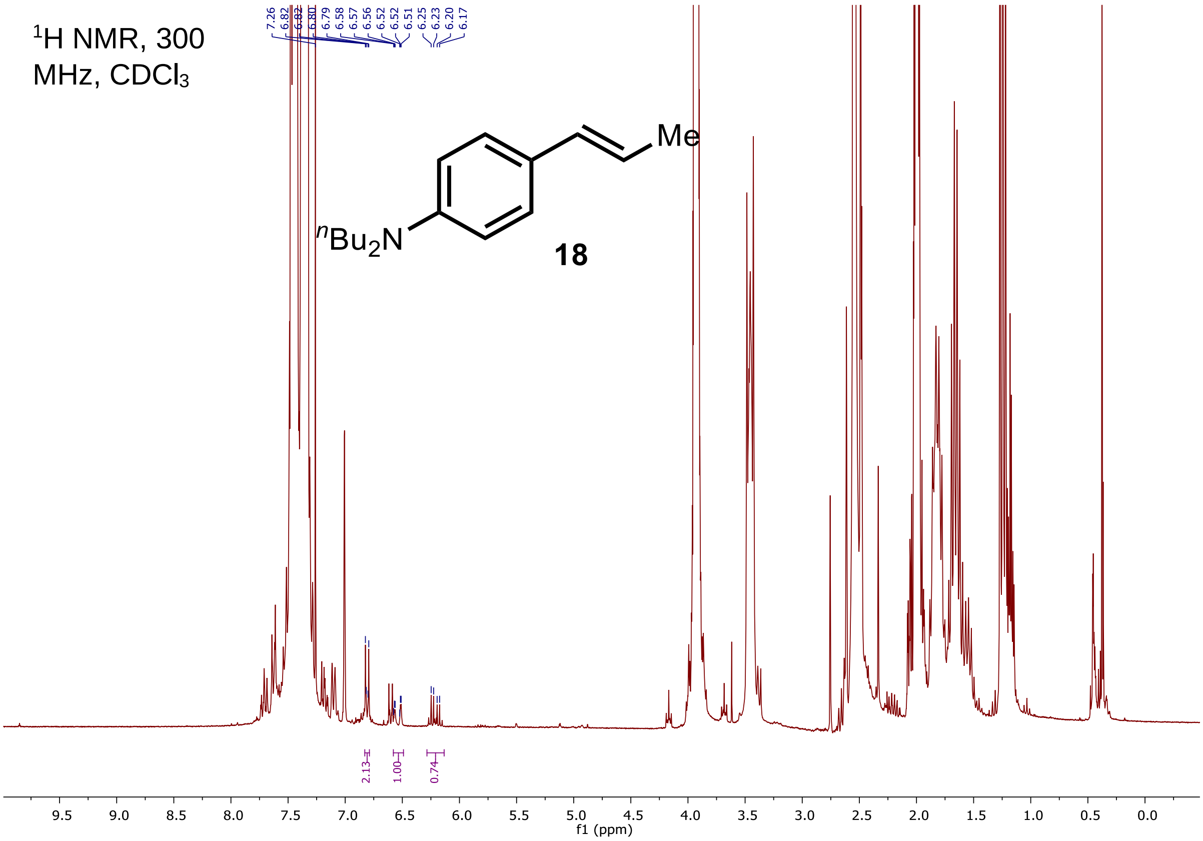

(*E*)-5-(prop-1-en-1-yl)benzo[*d*][1,3]dioxole (19)[24]

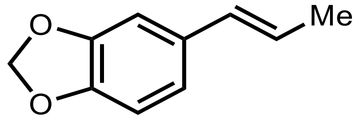

The title compound was prepared according to general procedure 3 using 5-iodobenzo[*d*][1,3]dioxole (0.1 mmol). Yield determined by crude ^{1}H NMR using 1,3,5-trimethylbenzene as internal standard: NMR yield = 85% (*E*:*Z* = 90:10).

<u>Resolved signals of the major isomer (*E*)-5-(prop-1-en-1-yl)benzo[*d*][1,3]dioxole:</u>

^{1}H NMR (400 MHz, Chloroform-*d*) δ 7.00 (d, *J* = 1.7 Hz, 1H), 6.84 (d, *J* = 1.5 Hz, 2H), 6.46 – 6.39 (m, 1H), 6.15 (dqd, *J* = 14.6, 6.6, 1.3 Hz, 1H), 5.91 (d, *J* = 1.3 Hz, 2H).

<u>Resolved signals of the minor isomer (*Z*)-5-(prop-1-en-1-yl)benzo[*d*][1,3]dioxole:</u>

^{1}H NMR (400 MHz, Chloroform-*d*) δ 5.93 (d, *J* = 1.4 Hz, 2H), 5.87 – 5.79 (m, 1H).

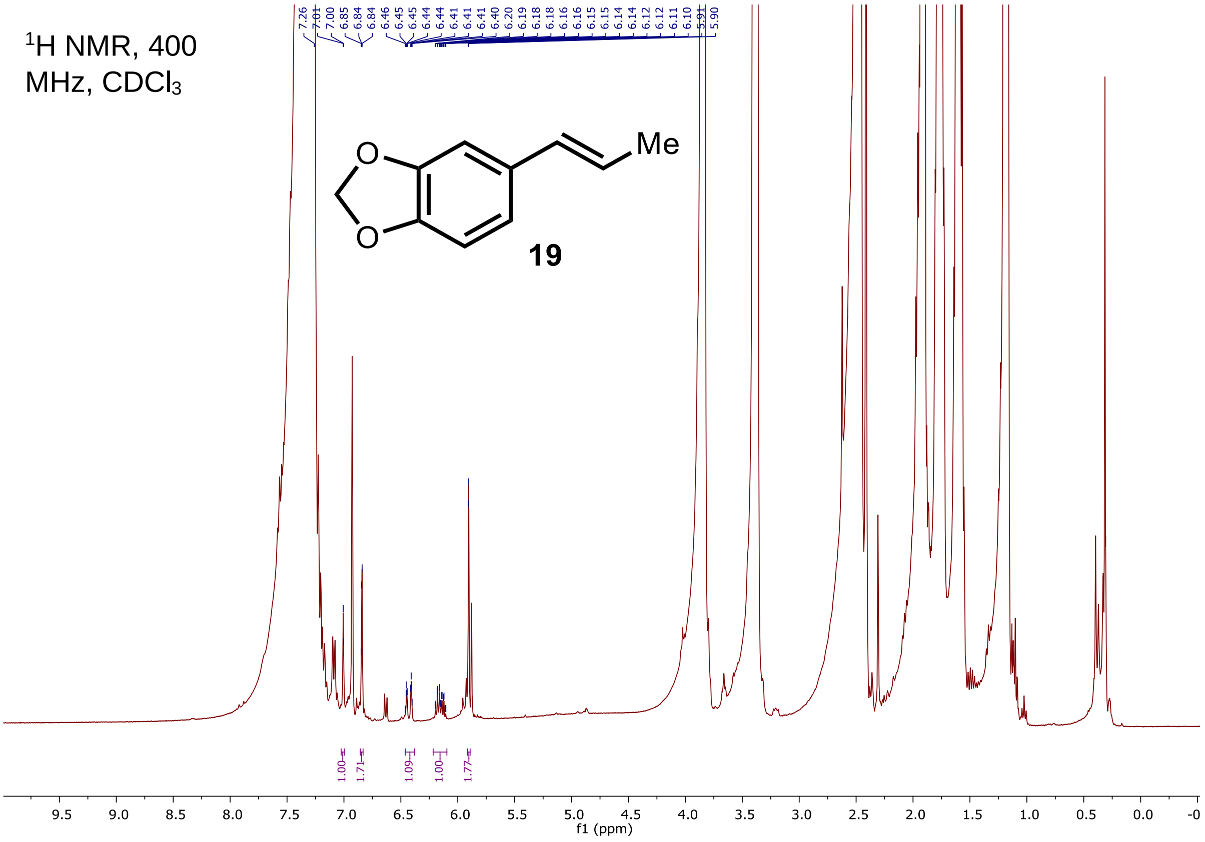

Butyl (*E*)-2-(4-(prop-1-en-1-yl)phenyl)acetate (20)

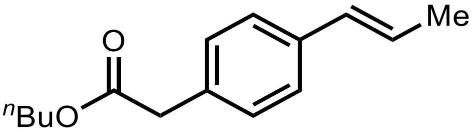

The title compound was prepared according to general procedure 3 using butyl 2-(4-iodophenyl)acetate (0.1 mmol). Yield determined by crude ^{1}H NMR using 1,3,5-trimethylbenzene as internal standard: NMR yield = 65% (*E*:*Z* = 93:7).

<u>Resolved signals of the major isomer butyl (*E*)-2-(4-(prop-1-en-1-yl)phenyl)acetate:</u>

^{1}H NMR (500 MHz, Chloroform-*d*) δ 6.65 (dq, *J* = 15.6, 1.8 Hz, 1H), 6.46 (dq, *J* = 15.7, 6.6 Hz, 1H), 4.36 (t, *J* = 6.7 Hz, 2H), 3.81 (s, 2H), 2.14 (dd, *J* = 6.6, 1.7 Hz, 3H), 1.26 (t, *J* = 7.3 Hz, 3H).

<u>Resolved signals of the minor isomer butyl (*E*)-2-(4-(prop-1-en-1-yl)phenyl)acetate:</u>

^{1}H NMR (500 MHz, Chloroform-*d*) δ 6.73 – 6.69 (m, 1H), 6.10 – 6.00 (m, 1H), 3.66 (s, 2H).

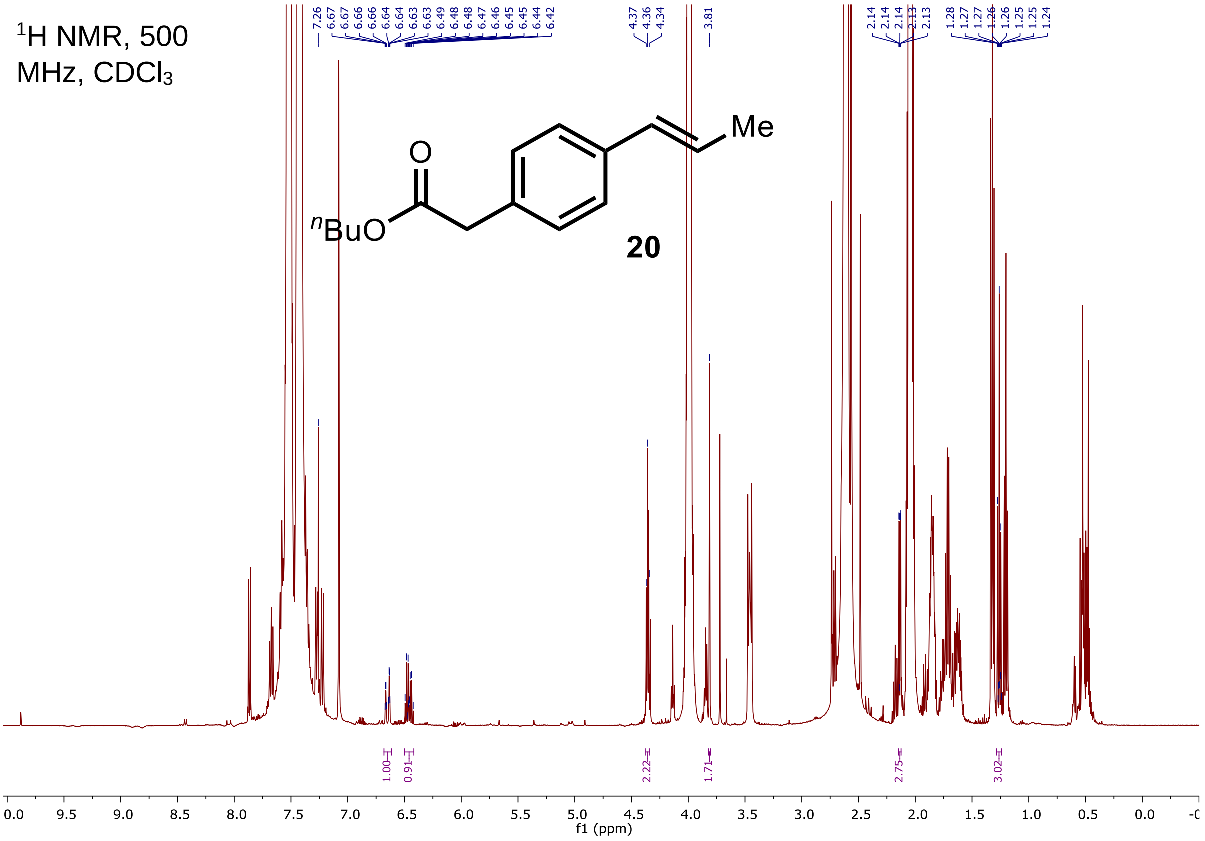

(*E*)-1-(prop-1-en-1-yl)-4-(trifluoromethyl)benzene (21)[15]

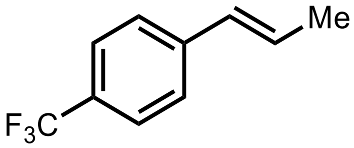

The title compound was prepared according to general procedure 3 using 1-iodo-4-(trifluoromethyl)benzene (0.1 mmol). Yield determined by crude ^{1}H NMR using 1,3,5-trimethylbenzene as internal standard: NMR yield = 85% (*E*:*Z* = 94:6).

Resolved signals of the major isomer (*E*)-1-(prop-1-en-1-yl)-4-(trifluoromethyl)benzene:

^{1}H NMR (300 MHz, Chloroform-*d*) δ 6.68 – 6.60 (m, 1H), 6.53 (dq, *J* = 15.7, 6.1 Hz, 1H), 2.15 (dd, *J* = 6.3, 1.2 Hz, 3H).

^{19}F NMR (471 MHz, Chloroform-*d*) δ -62.4.

Resolved signals of the minor isomer (*Z*)-1-(prop-1-en-1-yl)-4-(trifluoromethyl)benzene:

^{1}H NMR (300 MHz, Chloroform-*d*) δ 6.21 – 6.09 (m, 1H).

^{19}F NMR (471 MHz, Chloroform-*d*) δ -62.4.

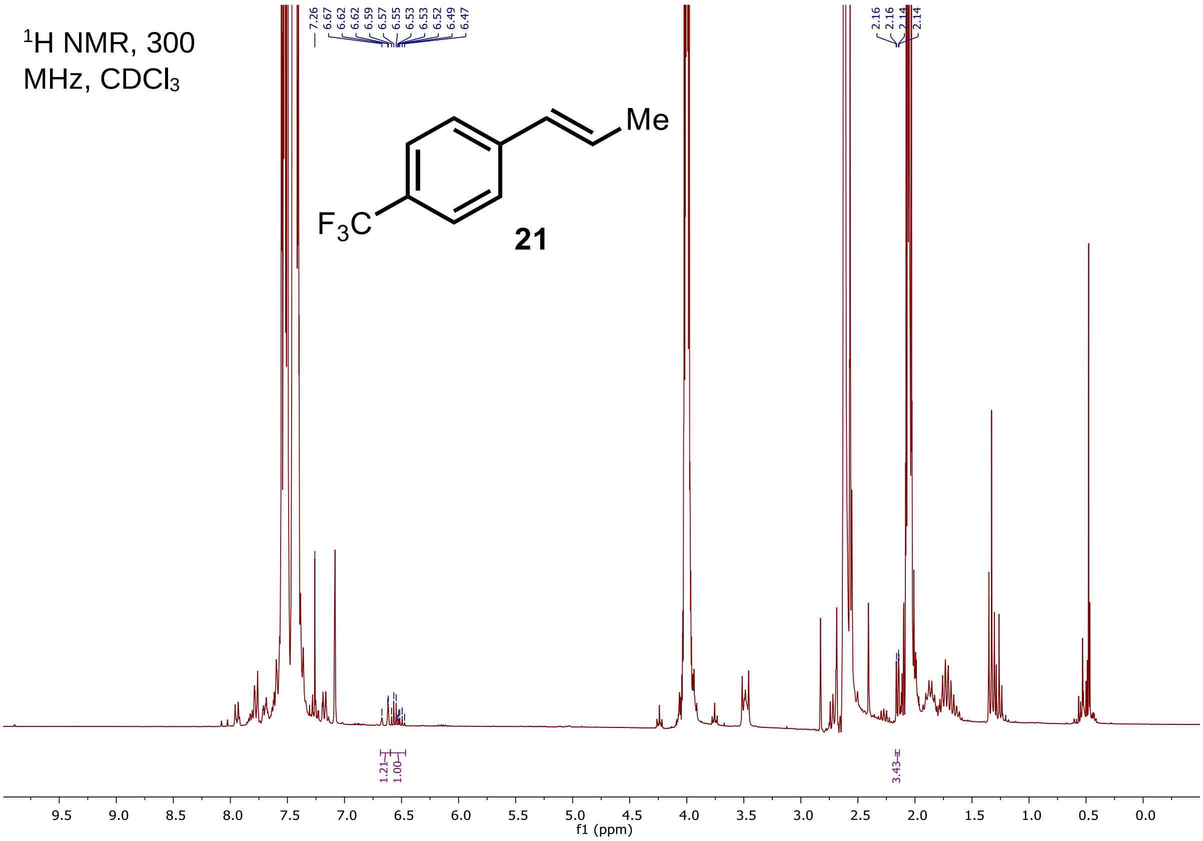

(*E*)-4-(prop-1-en-1-yl)benzonitrile (22)[25]

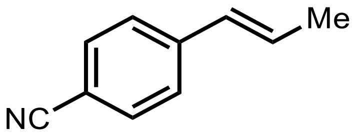

The title compound was prepared according to general procedure 3 using 4-iodobenzonitrile (0.1 mmol). Purification by flask silica chromatography (eluent = 20% EtOAc in PE) gave the title compound as yellow liquid (11.6 mg, 81%, *E*:*Z* = 92:8); Rf: 0.47 (eluent = 20% EtOAc in PE). NMR yield = 85% (*E*:*Z* = 92:8).

Signals of the major isomer (*E*)-4-(prop-1-en-1-yl)benzonitrile:

^{1}H NMR (300 MHz, Chloroform-*d*) δ 7.58 – 7.53 (m, 2H), 7.41 – 7.36 (m, 2H), 6.50 – 6.30 (m, 2H), 1.94 – 1.90 (m, 3H).

^{13}C NMR (75 MHz, Chloroform-*d*) δ 142.5, 132.5, 130.3, 129.9, 126.4, 119.3, 110.1, 18.8.

Resolved signals of the minor isomer (*Z*)-4-(prop-1-en-1-yl)benzonitrile:

^{1}H NMR (300 MHz, Chloroform-*d*) δ 7.88 – 7.82 (m, 2H), 7.64 – 7.59 (m, 2H), 5.95 (dq, *J* = 11.6, 7.2 Hz, 1H).

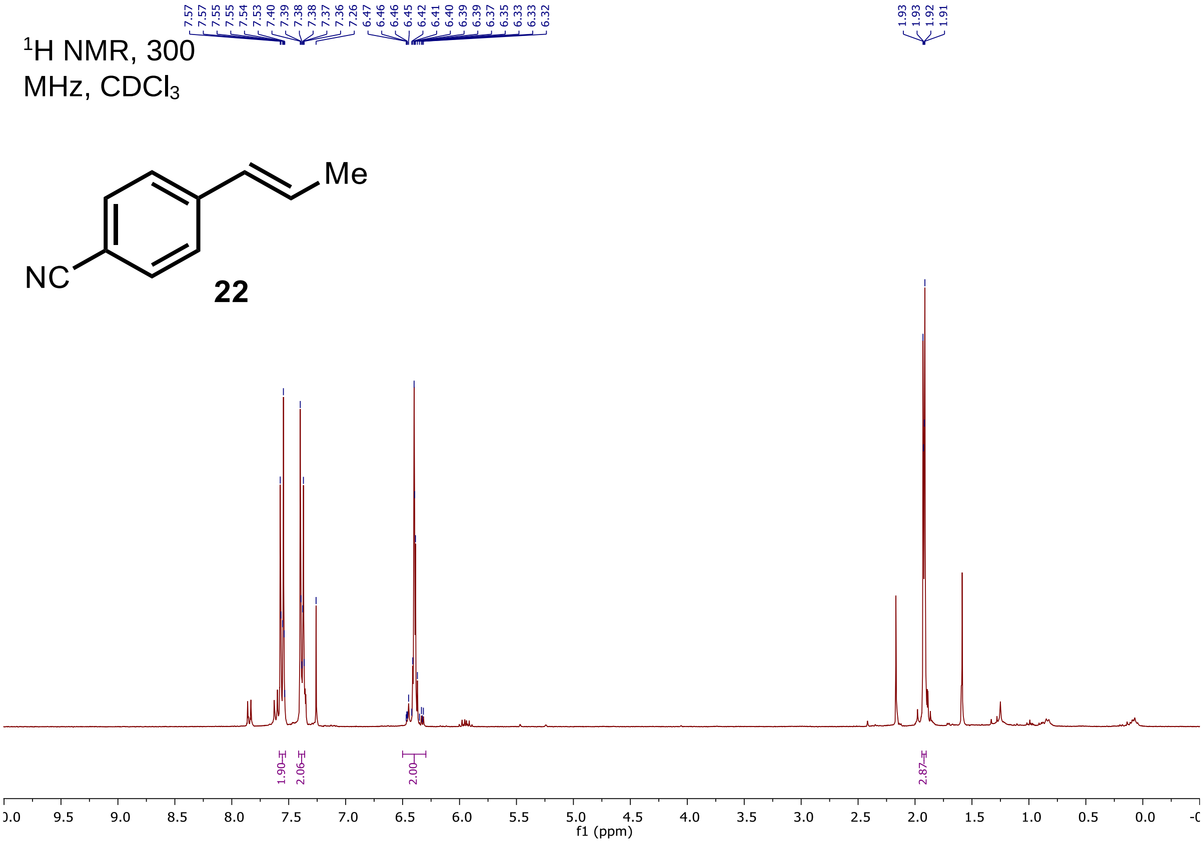

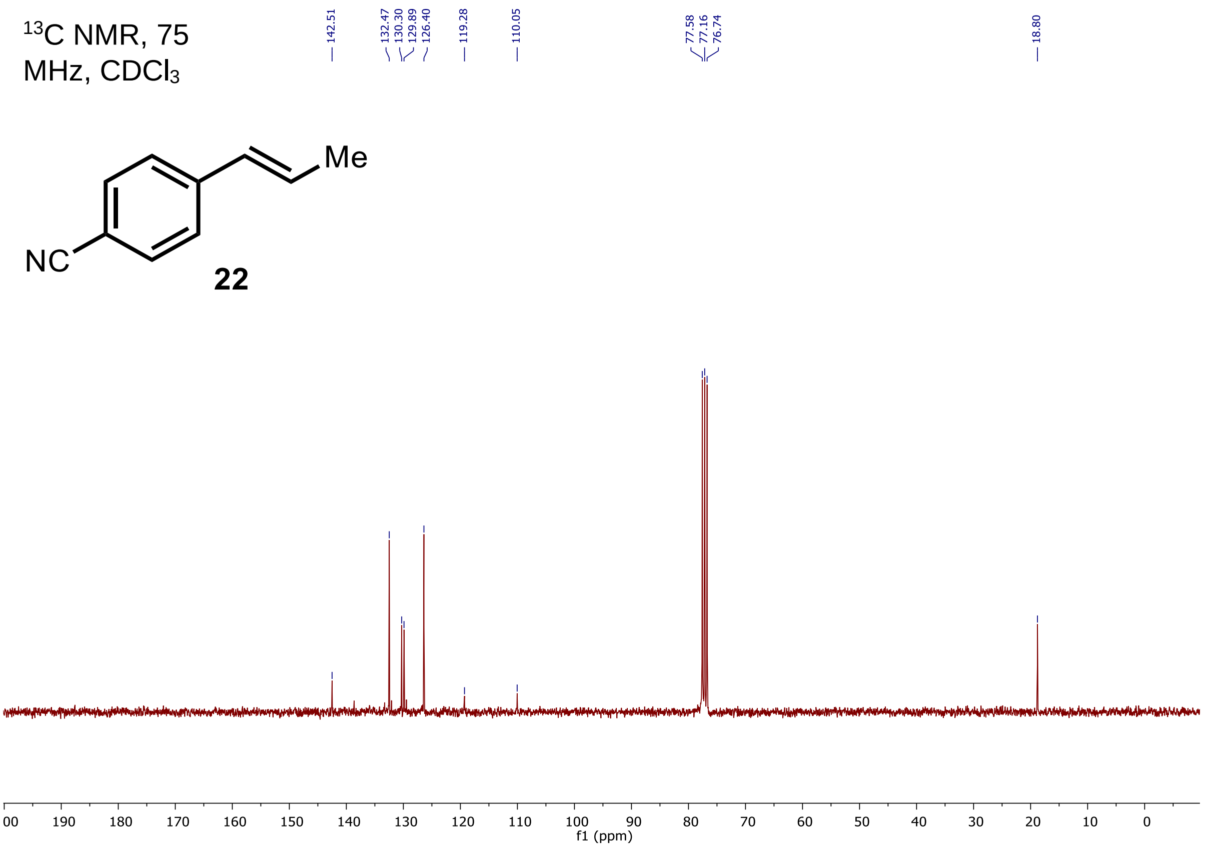

(*E*)-1-(4-(prop-1-en-1-yl)phenyl)ethan-1-one (23)[25]

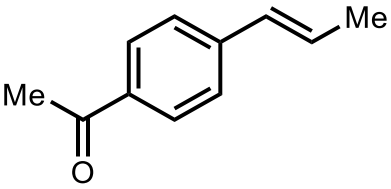

The title compound was prepared according to general procedure 3 using 1-(4-iodophenyl)ethan-1-one (0.1 mmol). Yield determined by crude ^{1}H NMR using 1,3,5-trimethylbenzene as internal standard: NMR yield = 82% (*E*:*Z* = 93:7).

Resolved signals of the major isomer (*E*)-1-(4-(prop-1-en-1-yl)phenyl)ethan-1-one:

^{1}H NMR (300 MHz, Chloroform-*d*) δ 8.13 – 8.09 (m, 2H), 6.69 – 6.50 (m, 2H), 2.73 (s, 3H), 2.16 – 2.13 (m, 3H).

Resolved signals of the minor isomer (*Z*)-1-(4-(prop-1-en-1-yl)phenyl)ethan-1-one:

^{1}H NMR (300 MHz, Chloroform-*d*) δ 6.20 – 6.07 (m, 1H), 2.71 (s, 3H).

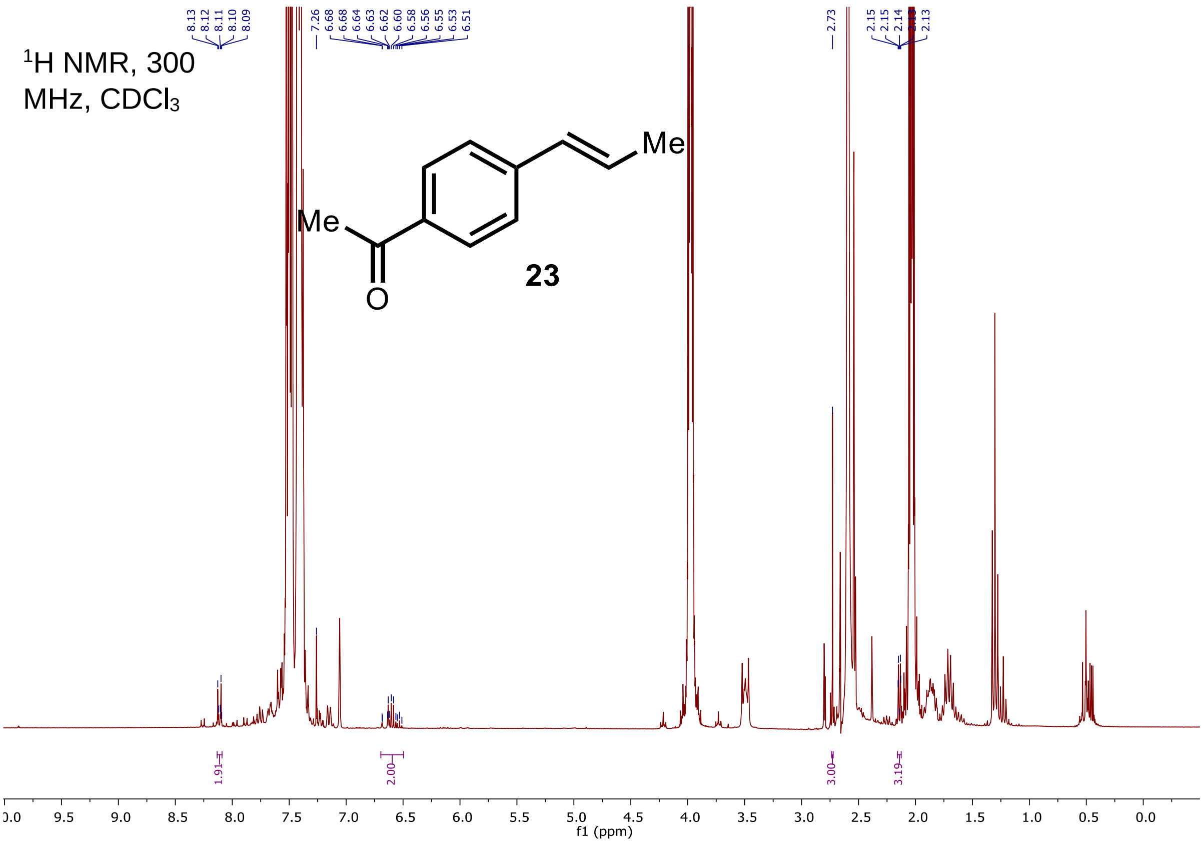

(*E*)-phenyl(4-(prop-1-en-1-yl)phenyl)methanone (24)[26]

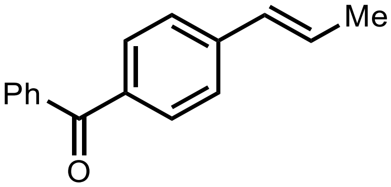

The title compound was prepared according to general procedure 3 using (4-iodophenyl)(phenyl)methanone (0.1 mmol). Yield determined by crude ^{1}H NMR using 1,3,5-trimethylbenzene as internal standard: NMR yield = 82% (*E*:*Z* = 89:11).

Resolved signals of the major isomer (*E*)-phenyl(4-(prop-1-en-1-yl)phenyl)methanone:

^{1}H NMR (500 MHz, Chloroform-*d*) δ 8.05 – 7.97 (m, 4H), 6.66 (dd, *J* = 15.8, 1.6 Hz, 1H), 6.58 (dq, *J* = 15.6, 6.3 Hz, 1H), 2.13 (dd, *J* = 6.4, 1.5 Hz, 3H).

Resolved signals of the minor isomer (*Z*)-phenyl(4-(prop-1-en-1-yl)phenyl)methanone:

^{1}H NMR (500 MHz, Chloroform-*d*) δ 6.73 – 6.69 (m, 1H), 6.13 (dq, *J* = 11.7, 7.3 Hz, 1H).

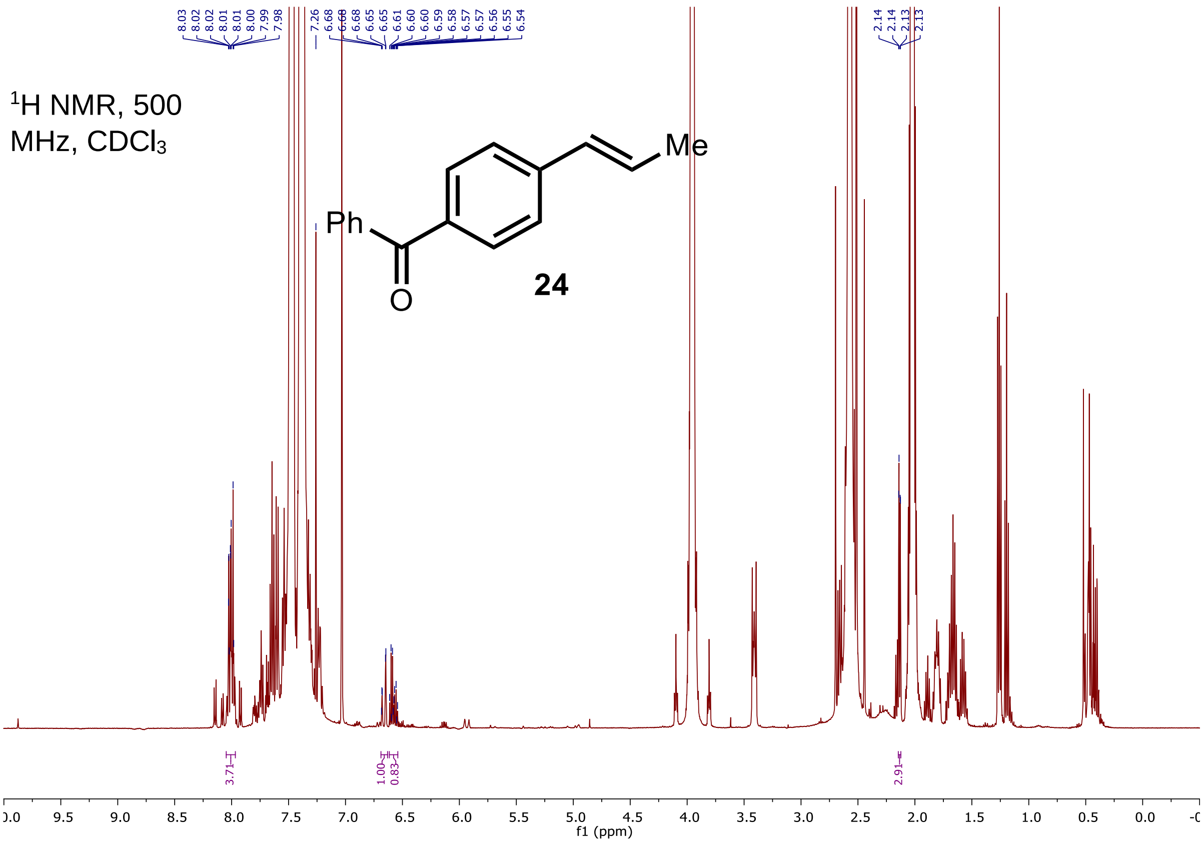

Ethyl (*E*)-4-(prop-1-en-1-yl)benzoate (25)[27]

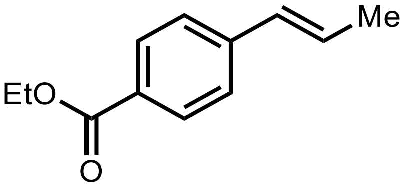

The title compound was prepared according to general procedure 3 using ethyl 4-iodobenzoate (0.1 mmol). Yield determined by crude ^{1}H NMR using 1,3,5-trimethylbenzene as internal standard: NMR yield = 98% (*E*:*Z* = 96:4).

Resolved signals of the major isomer ethyl (*E*)-4-(prop-1-en-1-yl)benzoate:

^{1}H NMR (300 MHz, Chloroform-*d*) δ 8.26 – 8.22 (m, 2H), 6.72 – 6.53 (m, 2H), 4.60 (q, *J* = 7.2 Hz, 2H).

Resolved signals of the minor isomer ethyl (*Z*)-4-(prop-1-en-1-yl)benzoate:

^{1}H NMR (300 MHz, Chloroform-*d*) δ 8.29 (d, *J* = 8.3 Hz, 2H), 6.15 (dd, *J* = 11.7, 7.3 Hz, 1H).

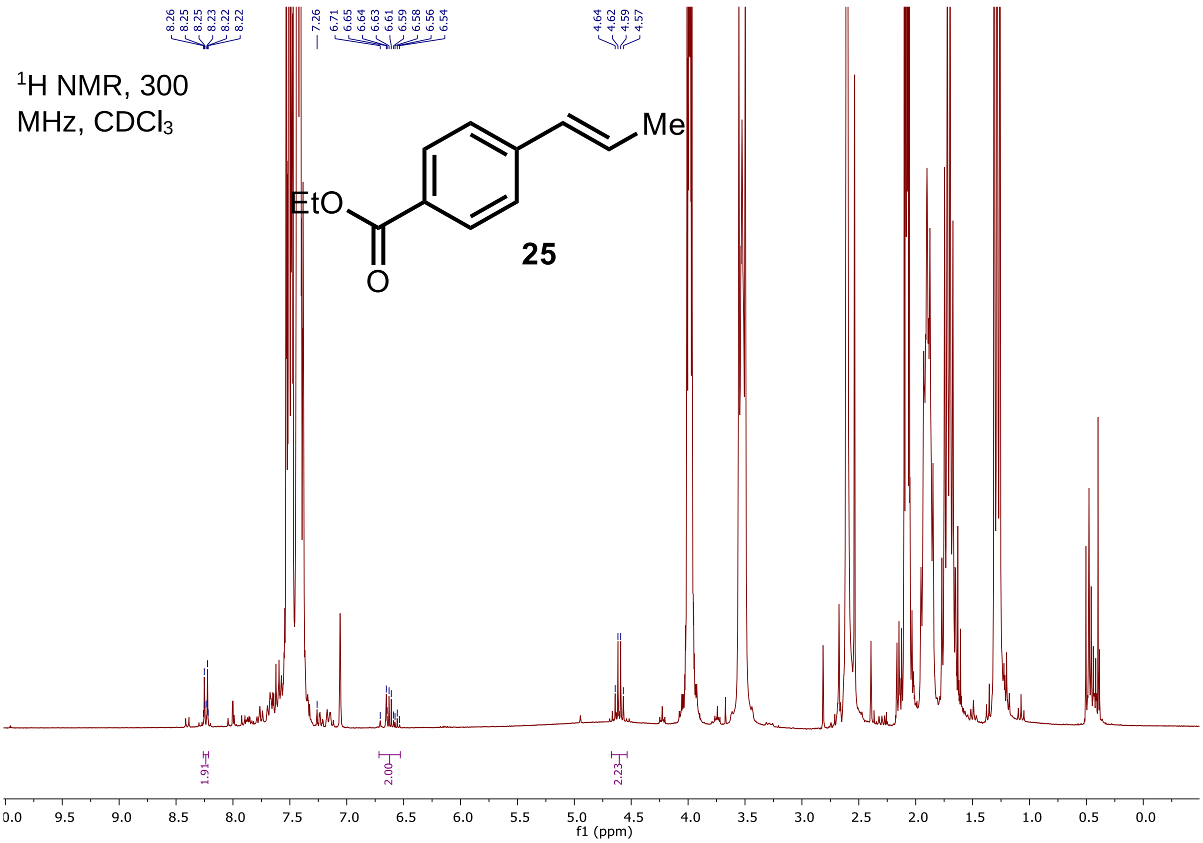

Phenyl (*E*)-4-(prop-1-en-1-yl)benzoate (26)

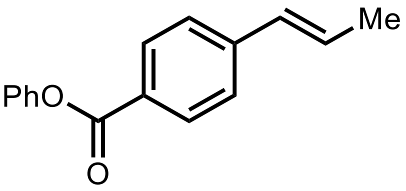

The title compound was prepared according to general procedure 3 using phenyl 4-iodobenzoate (0.1 mmol). Yield determined by crude ^{1}H NMR using 1,3,5-trimethylbenzene as internal standard: NMR yield = 78% (*E*:*Z* = 90:10).

<u>Resolved signals of the major isomer phenyl (*E*)-4-(prop-1-en-1-yl)benzoate:</u>

^{1}H NMR (500 MHz, Chloroform-*d*) δ 8.38 – 8.35 (m, 2H), 6.65 (dd, *J* = 15.8, 1.5 Hz, 1H), 6.62 – 6.53 (m, 1H), 2.13 (dd, *J* = 6.3, 1.4 Hz, 3H).

<u>Resolved signals of the minor isomer phenyl (*Z*)-4-(prop-1-en-1-yl)benzoate:</u>

^{1}H NMR (500 MHz, Chloroform-*d*) δ 8.42 – 8.39 (m, 2H), 6.72 – 6.68 (m, 1H), 6.13 (dq, *J* = 11.6, 7.3 Hz, 1H).

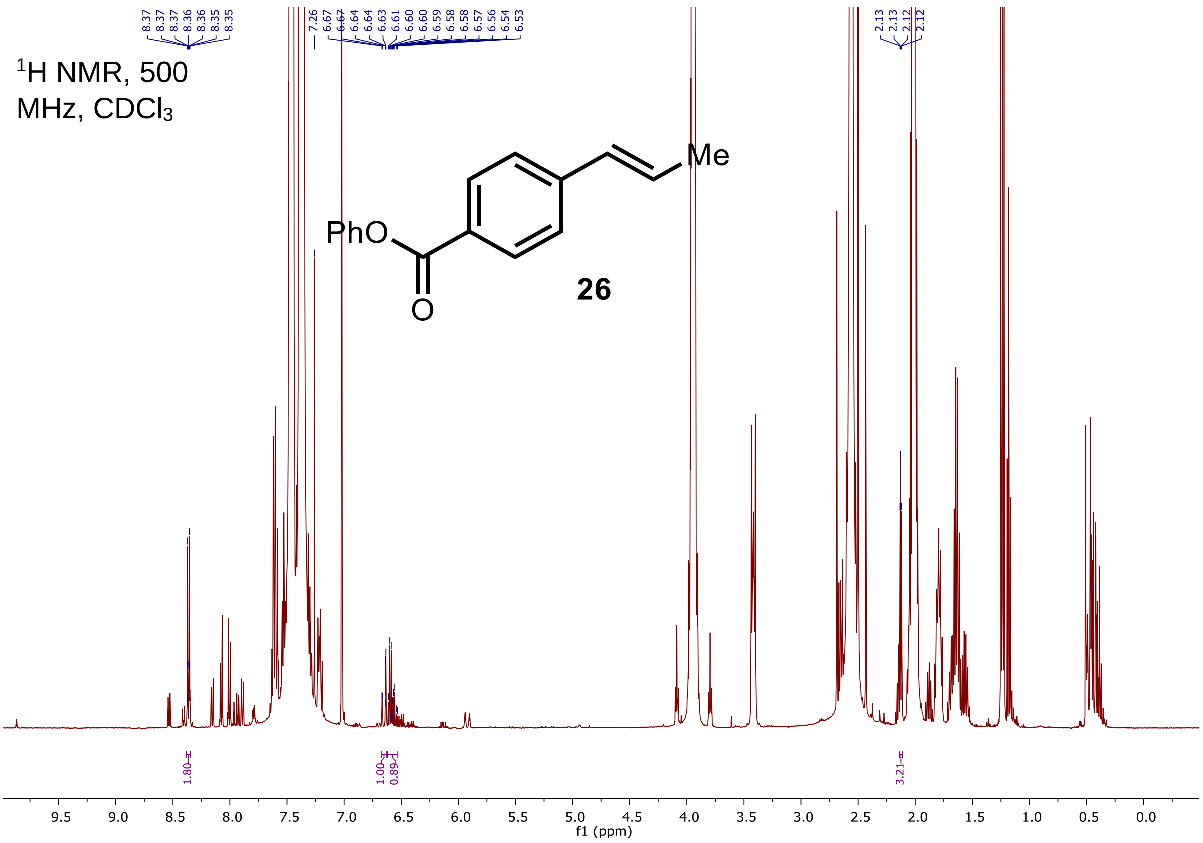

(*E*)-*N*,*N*-dimethyl-4-(prop-1-en-1-yl)benzenesulfonamide (27)

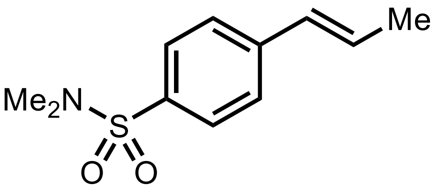

The title compound was prepared according to general procedure 3 using 4-iodo-*N*,*N*-dimethylbenzenesulfonamide (0.1 mmol). Yield determined by crude ^{1}H NMR using 1,3,5-trimethylbenzene as internal standard: NMR yield = 76% (*E*:*Z* = 95:5).

<u>Resolved signals of the major isomer (*E*)-*N*,*N*-dimethyl-4-(prop-1-en-1-yl)benzenesulfonamide:</u>

^{1}H NMR (300 MHz, Chloroform-*d*) δ 7.89 – 7.84 (m, 2H), 6.63 – 6.45 (m, 2H), 2.82 (s, 6H), 2.09 (d, *J* = 5.2 Hz, 3H).

<u>Resolved signals of minor isomer (*Z*)-*N*,*N*-dimethyl-4-(prop-1-en-1-yl)benzenesulfonamide:</u>

^{1}H NMR (300 MHz, Chloroform-*d*) δ 8.09 – 8.04 (m, 2H), 6.20 – 6.02 (m, 1H), 2.84 (s, 6H).

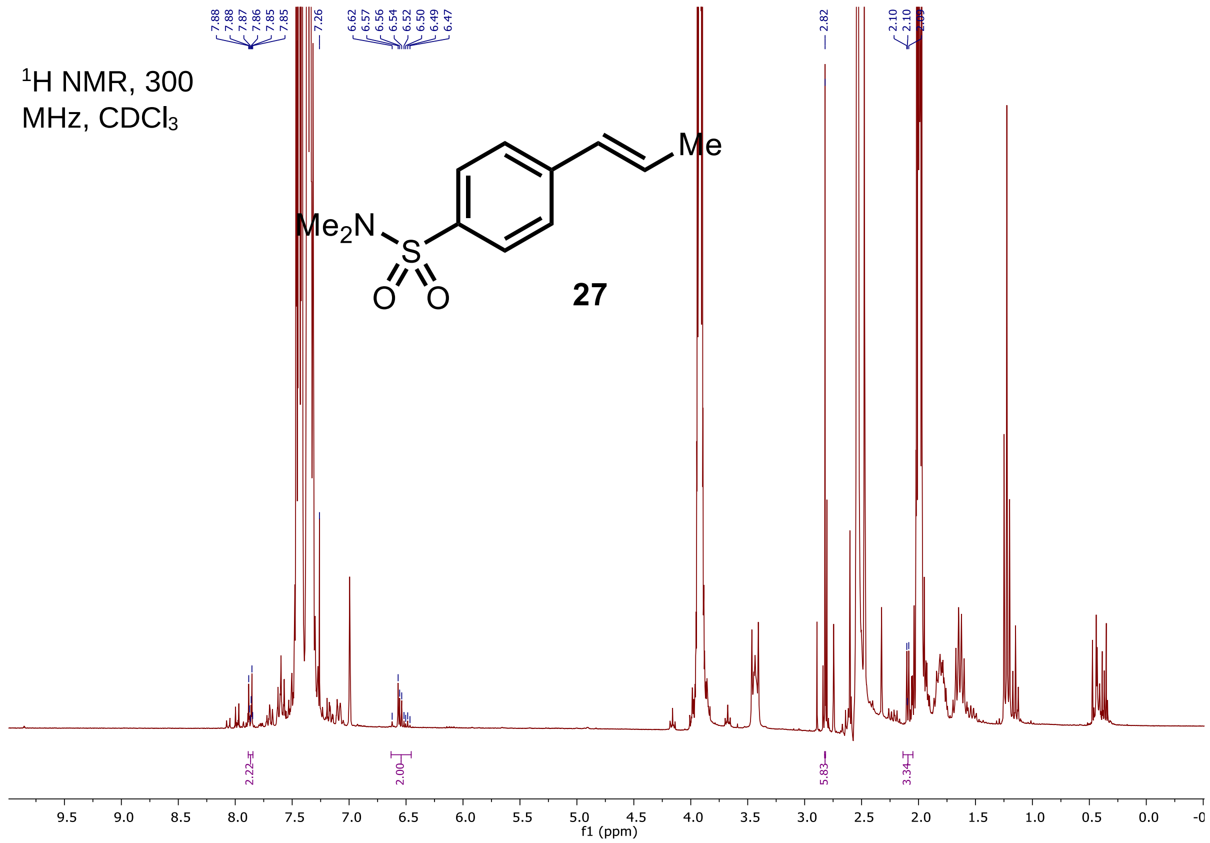

(*E*)-1-methyl-5-(prop-1-en-1-yl)-1*H*-indole (28)[24]

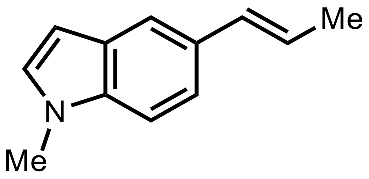

The title compound was prepared according to general procedure 3 using 5-Iodo-1-methyl-1*H*-indole (0.1 mmol). Yield determined by crude ^{1}H NMR using 1,3,5-trimethylbenzene as internal standard: NMR yield = 55% (*E*:*Z* = 93:7).

<u>Resolved signals of the major isomer (*E*)-1-methyl-5-(prop-1-en-1-yl)-1*H*-indole:</u>

^{1}H NMR (300 MHz, Chloroform-*d*) δ 7.78 (d, *J* = 1.6 Hz, 1H), 6.77 (dd, *J* = 15.7, 1.8 Hz, 1H), 6.66 (dd, *J* = 3.1, 0.9 Hz, 1H), 6.41 (dq, *J* = 15.7, 6.6 Hz, 1H), 3.82 (s, 3H), 2.13 (dd, *J* = 6.6, 1.7 Hz, 3H).

<u>Resolved signals of the minor isomer (*Z*)-1-methyl-5-(prop-1-en-1-yl)-1*H*-indole:</u>

^{1}H NMR (300 MHz, Chloroform-*d*) δ 5.97 (dd, *J* = 11.6, 7.2 Hz, 1H).

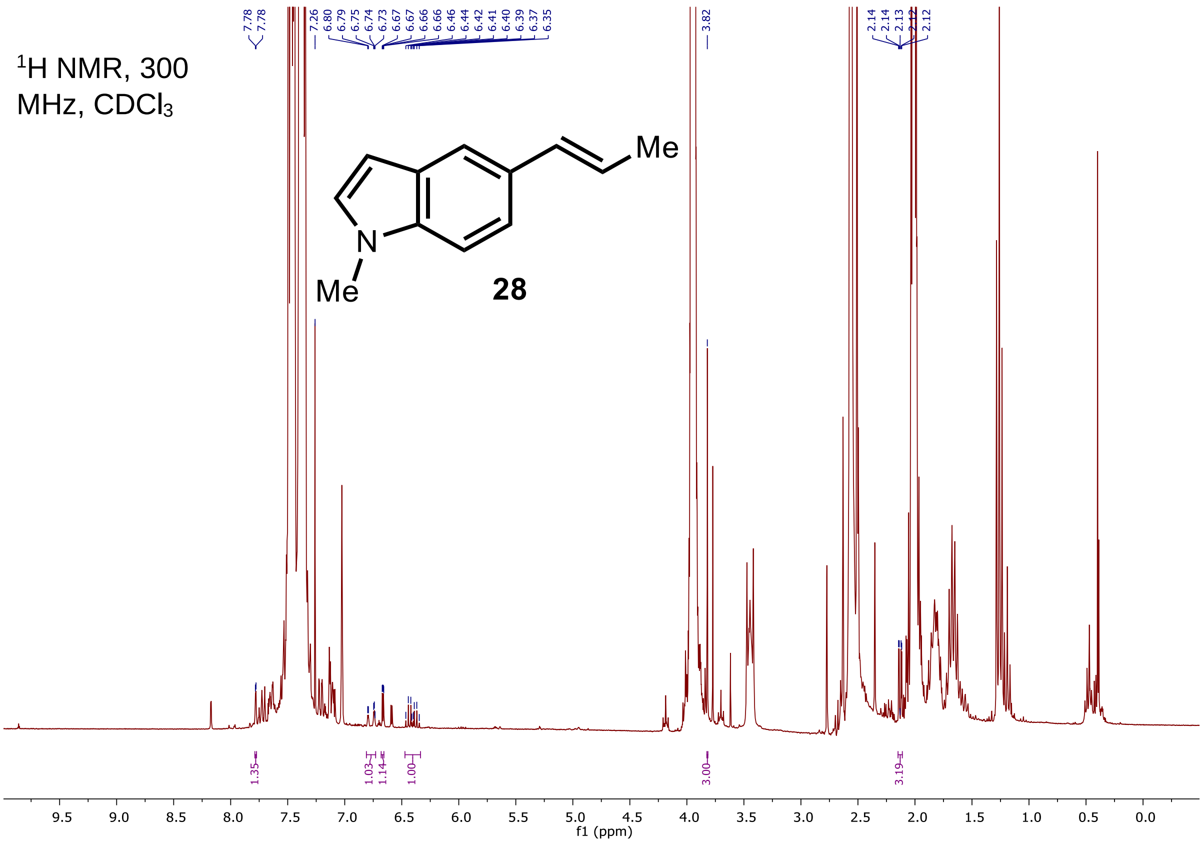

(*E*)-5-(prop-1-en-1-yl)-1-tosyl-1*H*-indole (29)

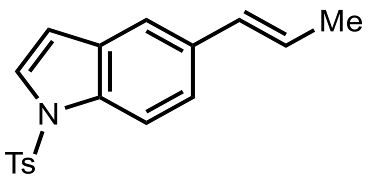

The title compound was prepared according to general procedure 3 using 5-Iodo-1-tosyl-1*H*-indole (0.1 mmol). Yield determined by crude ^{1}H NMR using 1,3,5-trimethylbenzene as internal standard: NMR yield = 53% (*E*:*Z* = 97:3).

<u>Resolved signals of the major isomer (*E*)-5-(prop-1-en-1-yl)-1-tosyl-1*H*-indole:</u>

^{1}H NMR (300 MHz, Chloroform-*d*) δ 8.16 (t, *J* = 0.8 Hz, 1H), 7.95 – 7.91 (m, 2H), 6.75 (dd, *J* = 3.7, 0.8 Hz, 1H), 6.71 – 6.58 (m, 1H), 6.38 (dq, *J* = 15.7, 6.5 Hz, 1H), 2.08 (dd, *J* = 6.6, 1.7 Hz, 3H).

<u>Resolved signals of the minor isomer (*Z*)-5-(prop-1-en-1-yl)-1-tosyl-1*H*-indole:</u>

^{1}H NMR (300 MHz, Chloroform-*d*) δ 6.05 – 5.94 (m, 1H).

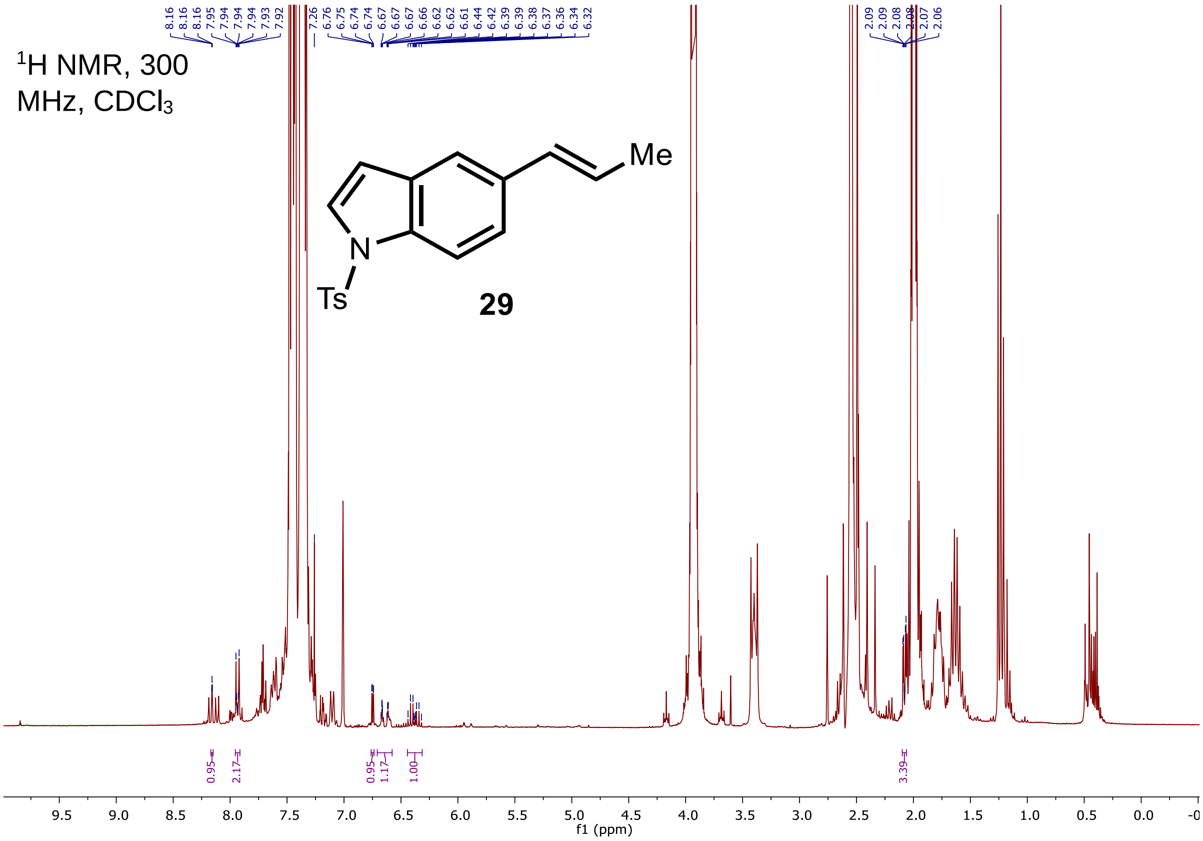

(*E*)-5-(prop-1-en-1-yl)benzofuran (30)[28]

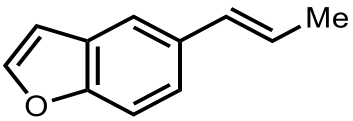

The title compound was prepared according to general procedure 3 using 5-Iodobenzofuran (0.1 mmol). Yield determined by crude ^{1}H NMR using 1,3,5-trimethylbenzene as internal standard: NMR yield = 70% (*E*:*Z* = 94:6).

Resolved signals of the major isomer (*E*)-5-(prop-1-en-1-yl)benzofuran:

^{1}H NMR (300 MHz, Chloroform-*d*) δ 6.90 (dd, *J* = 2.2, 0.9 Hz, 1H), 6.74 (dq, *J* = 15.8, 1.7 Hz, 1H), 6.43 (dq, *J* = 15.7, 6.6 Hz, 1H), 2.14 (dd, *J* = 6.6, 1.7 Hz, 3H).

Resolved signals of the minor isomer (*Z*)-5-(prop-1-en-1-yl)benzofuran:

^{1}H NMR (300 MHz, Chloroform-*d*) δ 6.04 (dd, *J* = 11.5, 7.2 Hz, 1H), 2.17 (dd, *J* = 7.2, 1.9 Hz, 3H).

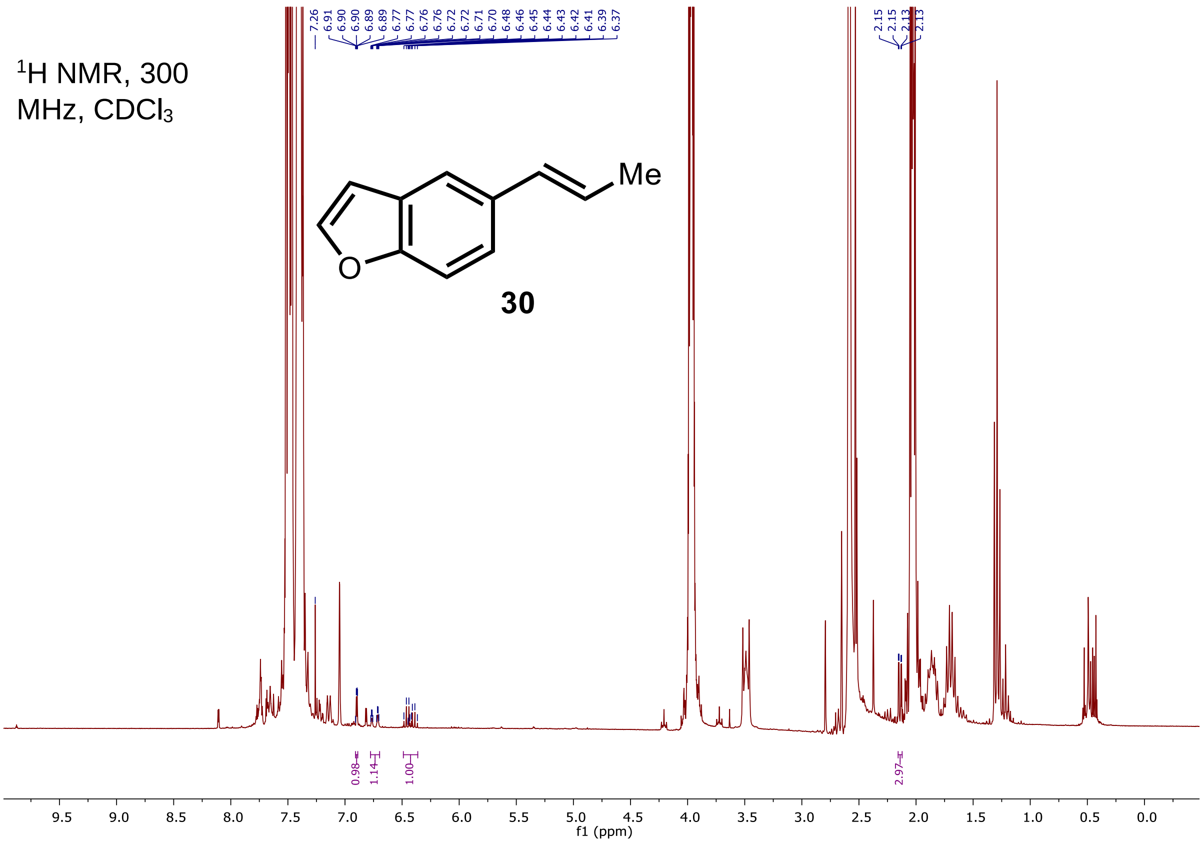

(*E*)-3-(prop-1-en-1-yl)pyridine (31)[29]

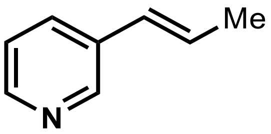

The title compound was prepared according to general procedure 3 using 3-iodopyridine (0.1 mmol). Yield determined by crude ^{1}H NMR using 1,3,5-trimethylbenzene as internal standard: NMR yiled = 68% (*E*:*Z* = 91:9).

<u>Signals of the major isomer (*E*)-3-(prop-1-en-1-yl)pyridine:</u>

^{1}H NMR (500 MHz, Chloroform-*d*) δ 9.06 (dd, *J* = 2.2, 0.8 Hz, 1H), 8.70 (dd, *J* = 4.7, 1.5 Hz, 1H), 8.05 (ddd, *J* = 8.1, 2.2, 1.5 Hz, 1H), 7.11 (ddd, *J* = 8.0, 4.7, 0.8 Hz, 1H), 6.57 – 6.52 (m, 1H), 6.52 – 6.42 (m, 1H), 2.11 – 2.09 (m, 3H).

<u>Resolved signals of the minor isomer (*Z*)-3-(prop-1-en-1-yl)pyridine:</u>

^{1}H NMR (500 MHz, Chloroform-*d*) δ 8.81 (d, *J* = 2.2 Hz, 1H), 8.67 (dd, *J* = 4.8, 1.7 Hz, 1H), 6.58 (dd, *J* = 11.7, 2.0 Hz, 1H).

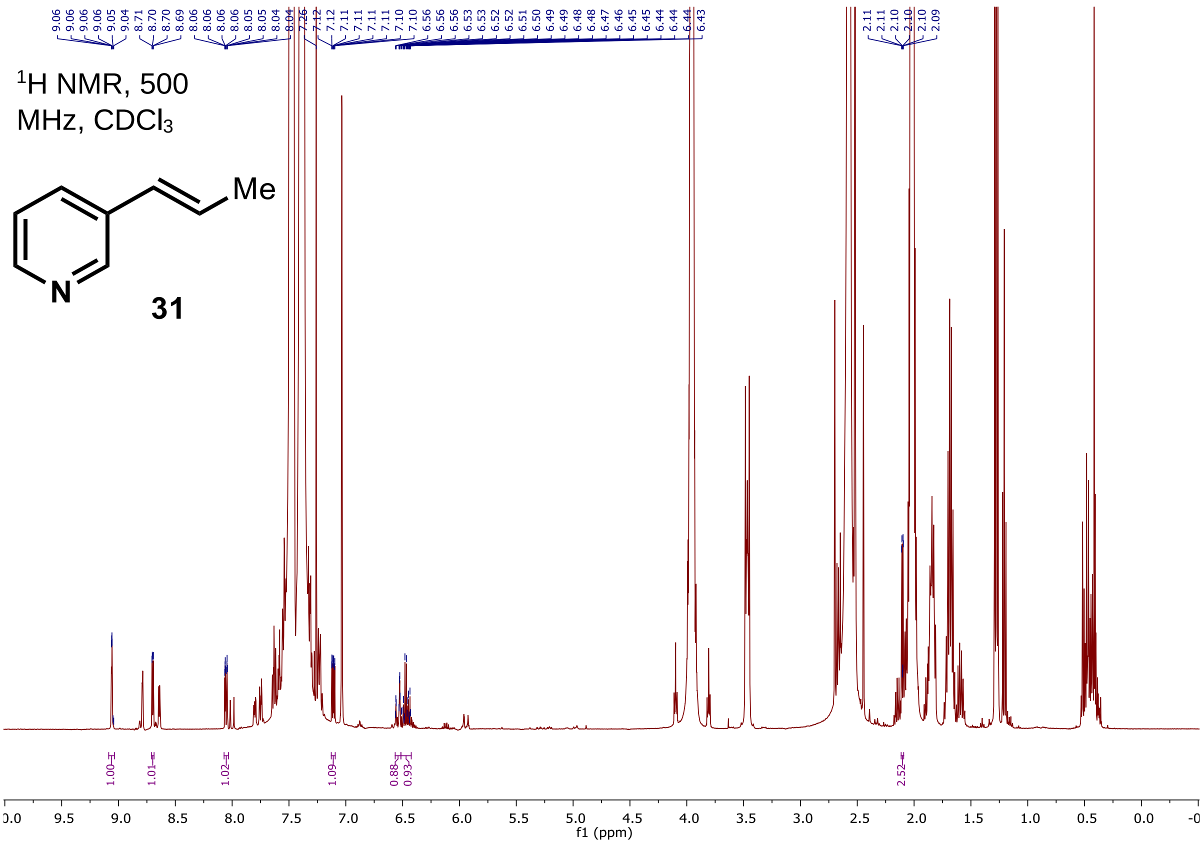

2.3.4. Epoxidation of (*E*)-triphenyl(prop-1-en-1-yl)silane (32)[30]

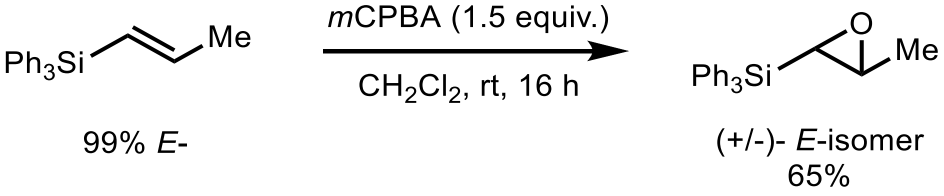

In 25 mL round-bottomed flask was charged with (*E*)-triphenyl(prop-1-en-1-yl)silane (0.2 mmol), *m*CPBA (0.3 mmol), and dry CH_2Cl_2 (2 mL). The reaction was stirred at rt for 16 h. Then sat. Na_2SO_4 and EtOAc (10 mL) was added and organic phase was separated, washed with sat. $NaHCO_3$, then brine, dried over $MgSO_4$, filtered, concentrated *in vacuo*. Purification by flask silica chromatography (eluent = 10% EtOAc in PE) gave the epoxidation product as white solid (41 mg, 65%, only *E*-); mp 92–93 ˚C; Rf: 0.32 (eluent = 10% EtOAc in PE); **^{1}H NMR (300 MHz, Chloroform-*d*)** δ 7.59 – 7.55 (m, 6H), 7.48 – 7.35 (m, 9H), 2.85 (qd, *J* = 5.1, 3.4 Hz, 1H), 2.71 – 2.67 (m, 1H), 1.44 (d, *J* = 5.1 Hz, 3H); **^{13}C NMR (75 MHz, Chloroform-*d*)** δ 136.0, 130.2, 128.2, 52.5, 50.6, 19.4; **LRMS** (ES) calculated $[C_{11}H_{16}OSiNa]^+$ $(M+Na)^+$: m/z 339.20. The compound was previously reported, but not spectroscopically characterized.

^{1}H NMR, 300 MHz, $CDCl_3$

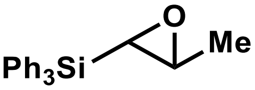

(+/-)- *E*-isomer

32

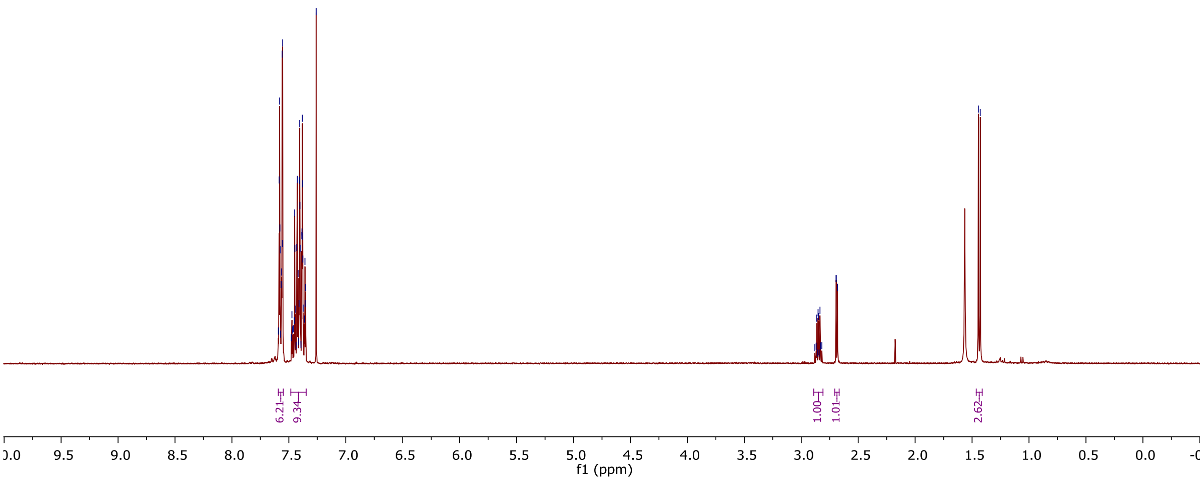

^{13}C NMR, 75 MHz, $CDCl_3$

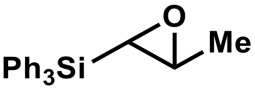

(+/-)- *E*-isomer

32

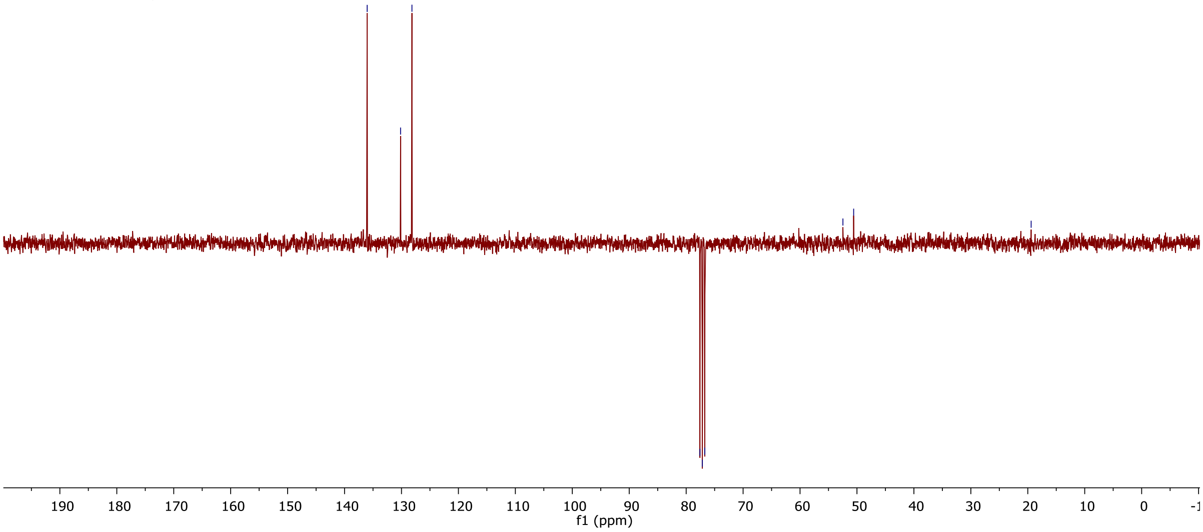

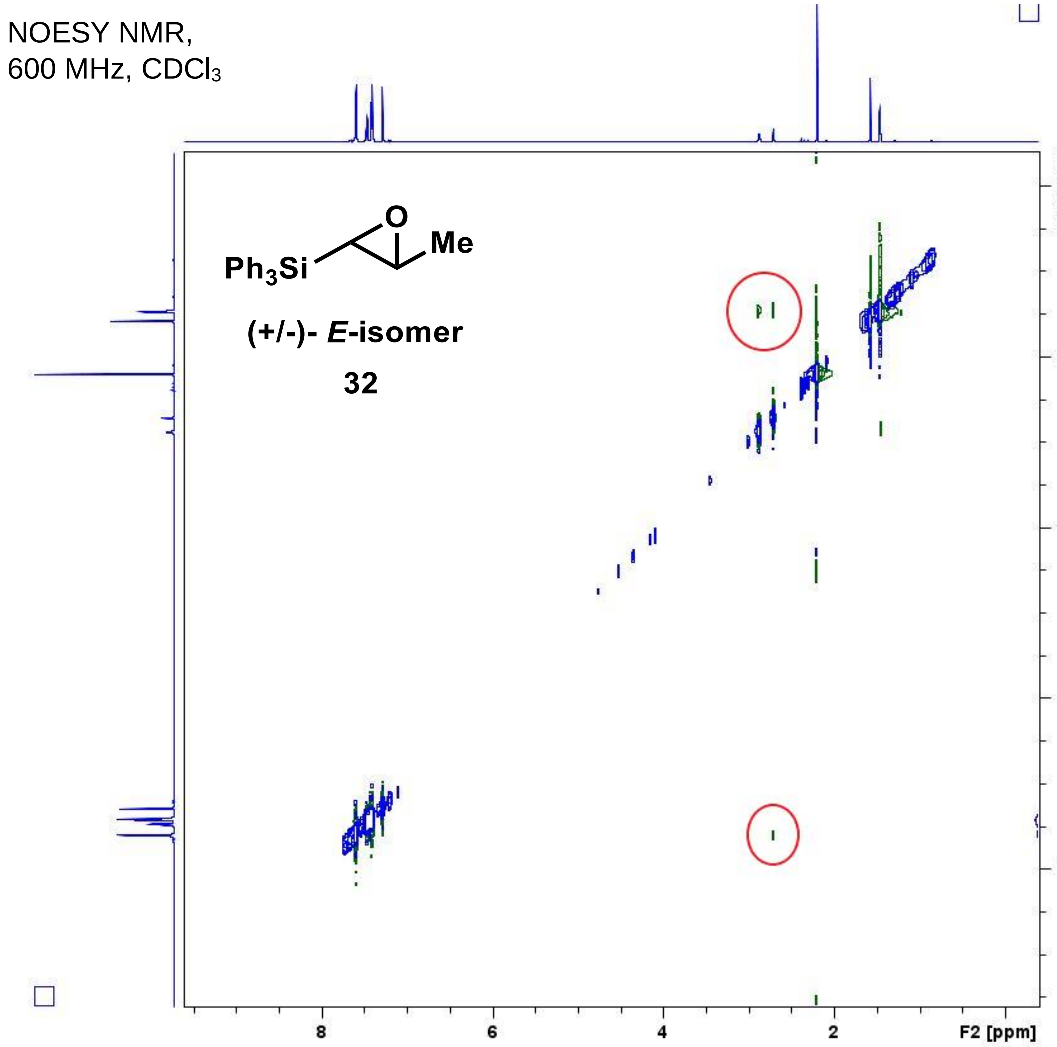

2.4. Intermediates trapping

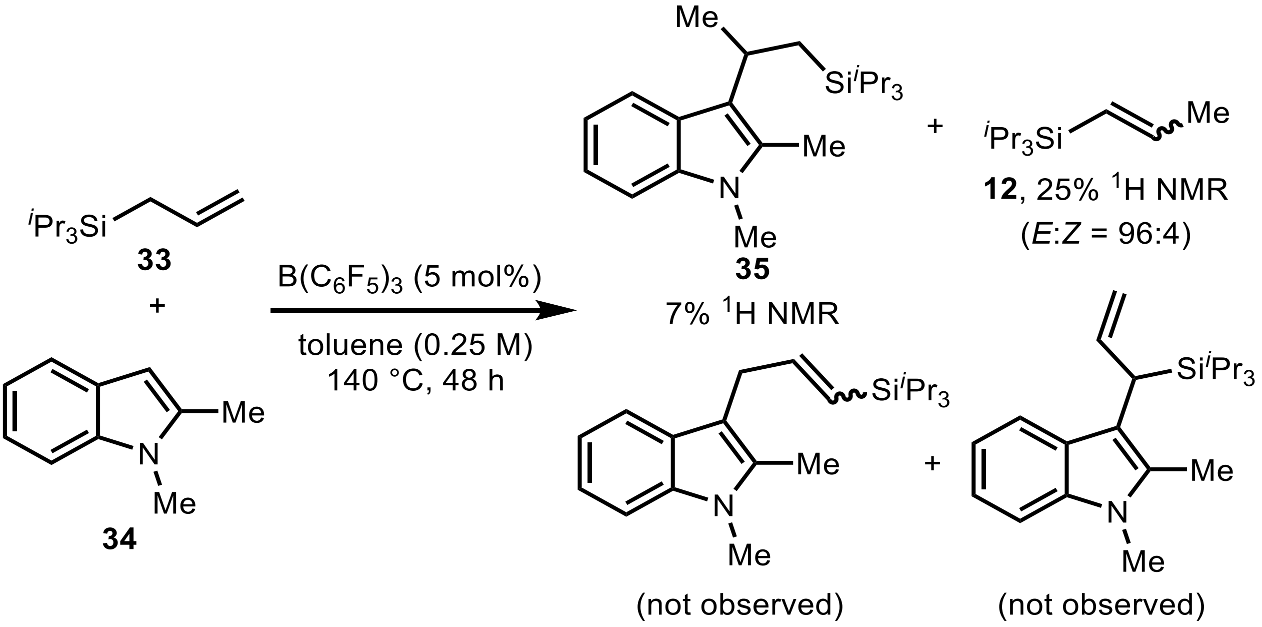

In the glovebox under Ar, an oven-dried 10 mL microwave vial equipped with a magnetic stirrer bar was charged with $B(C_6F_5)_3$ (5 mol %), allyltriisopropylsilane (0.1 mmol), 1,2-dimethylindole (0.12 mmol), and toluene (0.4 mL). The vial was sealed with an aluminium crimp cap and stirred at 140 ˚C for 48 h. It was cooled to rt, 1,3,5-trimethylbenzene (15 µL, 0.1 mmol) was added and analysed using ^{1}H NMR. Purification was done by quenching with brine (0.4 mL). The organic phase was separated, dried over $MgSO_4$, filtered, and concentrated *in vacuo*. Purification by flask silica chromatography (eluent = 10% EtOAc in PE) gave compound **36** as yellow oil (4 mg, 12%); Rf: 0.65 (eluent = 10% EtOAc in PE); **^{1}H NMR (500 MHz, Chloroform-*d*)** δ 7.72 (dt, *J* = 7.9, 1.0 Hz, 1H), 7.23 (dt, *J* = 8.2, 0.9 Hz, 1H), 7.12 (ddd, *J* = 8.2, 7.0, 1.2 Hz, 1H), 7.03 (ddd, *J* = 8.0, 7.0, 1.1 Hz, 1H), 3.62 (s, 3H), 3.26 (td, *J* = 7.3, 5.9 Hz, 1H), 2.35 (s, 3H), 1.44 (d, *J* = 7.0 Hz, 3H), 1.34 – 1.29 (m, 1H), 1.31 – 1.22 (m, 1H), 1.04 (dt, *J* = 3.5, 2.1 Hz, 18H), 0.98 – 0.93 (m, 3H); **^{13}C NMR (126 MHz, Chloroform-*d*)** δ 137.1, 130.5, 126.2, 120.2, 119.9, 119.5, 118.2, 108.8, 29.9, 29.5, 27.1, 25.4, 19.2, 19.0, 11.6; **IR** (film, ν_{max} / cm^{-1}) 2938, 2922, 2864, 1661, 1614, 1468, 1368, 1015, 881, 740; **HRMS** (CI) calculated $[C_{22}H_{37}NSi]^+$ $(M)^+$: m/z 343.2689, found 343.2682.

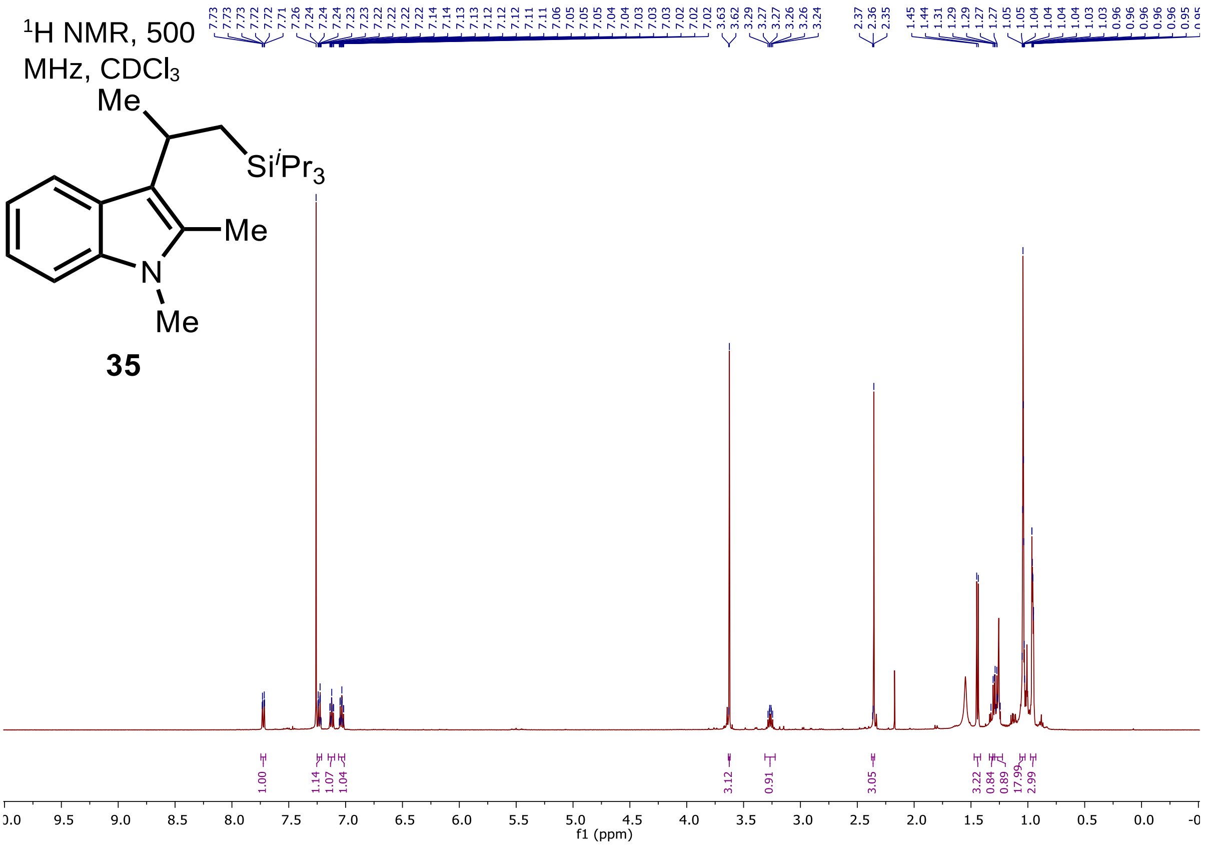

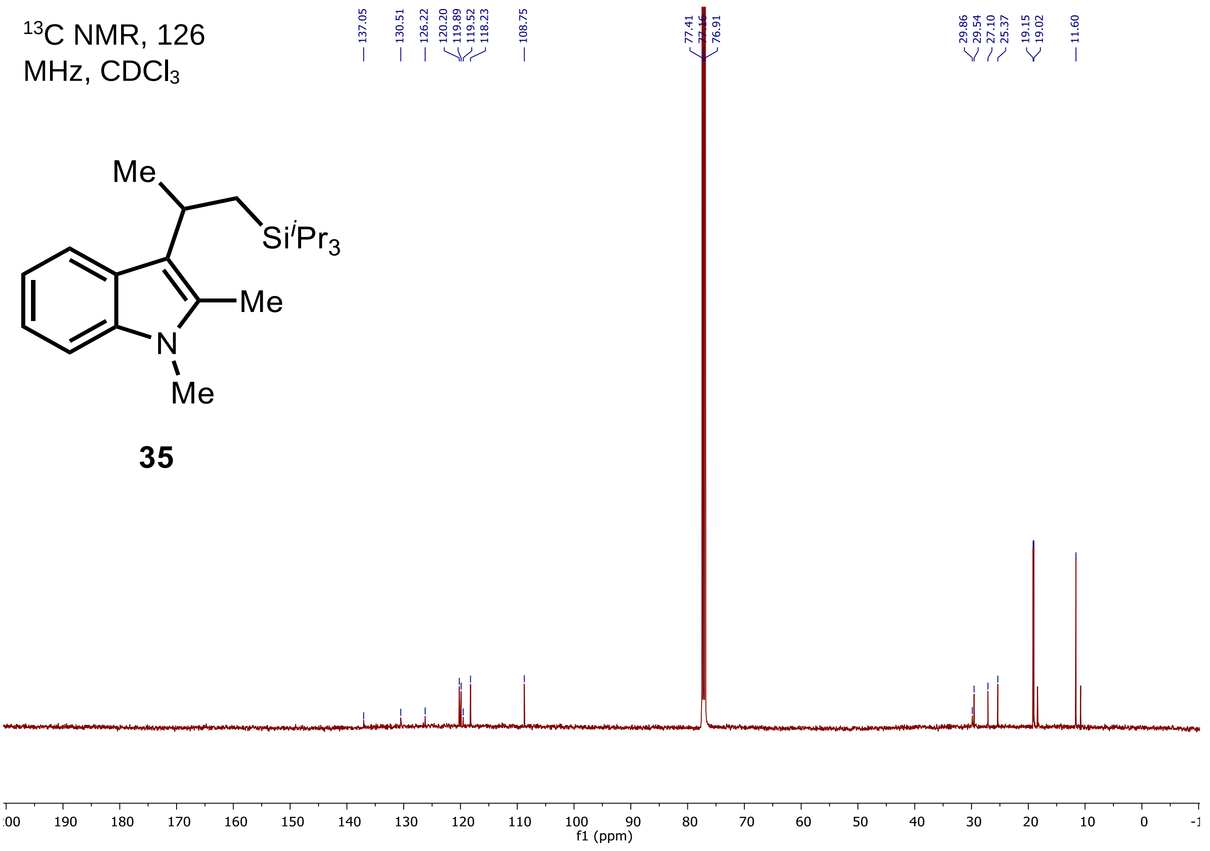

35

3. References

(1) Xue, W.; Oestreich, M. Silicon Grignard Reagents as Nucleophiles in Transition-Metal-Catalyzed Allylic Substitution. *Synthesis (Germany)* **2019**, *51* (1), 233–239. https://doi.org/10.1055/s-0037-1610309.

(2) Hirone, N.; Sanjiki, H.; Tanaka, R.; Hata, T.; Urabe, H. Acceleration of the Substitution of Silanes with Grignard Reagents by Using Either LiCl or YCl3/MeLi. *Angewandte Chemie - International Edition* **2010**, *49* (42), 7762–7764. https://doi.org/10.1002/anie.201003174.

(3) Shimizu, R.; Egami, H.; Hamashima, Y.; Sodeoka, M. Copper-Catalyzed Trifluoromethylation of Allylsilanes. *Angewandte Chemie - International Edition* **2012**, *51* (19), 4577–4580. https://doi.org/10.1002/anie.201201095.

(4) Dunn, J.; Dobbs, A. P. Synthesis and Reactions of Donor Cyclopropanes: Efficient Routes to Cis- and Trans-Tetrahydrofurans. *Tetrahedron* **2015**, *71* (39), 7386–7414. https://doi.org/10.1016/j.tet.2015.05.007.

(5) Carre Corriu Et B Henner, F. R. *PROBLEME DU CATION SILICONIUM IV. MECANISME DE LA COUPURE METAL-CARBONE DANS LES DERIVES /3-FONCTIONNELS DU SILICIUM ET DU GERMANIUM*.

(6) Grzelak, M.; Fra¸ckowiak, D.; Januszewski, R.; Marciniec, B. Introduction of Organogermyl Functionalities to Cage Silsesquioxanes. *Dalton Transactions* **2020**, *49* (16), 5055–5063. https://doi.org/10.1039/d0dt00557f.

(7) Rondeau-Gagné, S.; Curutchet, C.; Grenier, F.; Scholes, G. D.; Morin, J. F. Synthesis, Characterization and DFT Calculations of New Ethynyl-Bridged C60 Derivatives. *Tetrahedron* **2010**, *66* (23), 4230–4242. https://doi.org/10.1016/j.tet.2010.03.092.

(8) Caramenti, P.; Nandi, R. K.; Waser, J. Metal-Free Oxidative Cross Coupling of Indoles with Electron-Rich (Hetero)Arenes. *Chemistry - A European Journal* **2018**, *24* (40), 10049–10053. https://doi.org/10.1002/chem.201802142.

(9) Niu, Y. J.; Sui, G. H.; Zheng, H. X.; Shan, X. H.; Tie, L.; Fu, J. le; Qu, J. P.; Kang, Y. B. Competing Dehalogenation versus Borylation of Aryl Iodides and Bromides under Transition-Metal-Free Basic Conditions. *Journal of Organic Chemistry* **2019**, *84* (17), 10805–10813. https://doi.org/10.1021/acs.joc.9b01350.

(10) Boehm, P.; Roediger, S.; Bismuto, A.; Morandi, B. Palladium-Catalyzed Chlorocarbonylation of Aryl (Pseudo)Halides Through In Situ Generation of Carbon Monoxide. *Angewandte Chemie - International Edition* **2020**, *59* (41), 17887–17896. https://doi.org/10.1002/anie.202005891.

(11) Garnier, J.; Thomson, D. W.; Zhou, S.; Jolly, P. I.; Berlouis, L. E. A.; Murphy, J. A. Hybrid Super Electron Donors - Preparation and Reactivity. *Beilstein Journal of Organic Chemistry* **2012**, *8*, 994–1002. https://doi.org/10.3762/bjoc.8.112.

(12) Ming, X. X.; Tian, Z. Y.; Zhang, C. P. Base-Mediated O-Arylation of Alcohols and Phenols by Triarylsulfonium Triflates. *Chem Asian J* **2019**, *14* (19), 3370–3379. https://doi.org/10.1002/asia.201900968.

(13) Wang, X.; Pan, S.; Luo, Q.; Wang, Q.; Ni, C.; Hu, J. Controllable Single and Double Difluoromethylene Insertions into C-Cu Bonds: Copper-Mediated Tetrafluoroethylation and Hexafluoropropylation of Aryl Iodides with TMSCF2H and TMSCF2Br. *J Am Chem Soc* **2022**, *144* (27), 12202–12211. https://doi.org/10.1021/jacs.2c03104.

(14) Debergh, J. R.; Niljianskul, N.; Buchwald, S. L. Synthesis of Aryl Sulfonamides via Palladium-Catalyzed Chlorosulfonylation of Arylboronic Acids. *J Am Chem Soc* **2013**, *135* (29), 10638–10641. https://doi.org/10.1021/ja405949a.

(15) Kawamura, K. E.; Chang, A. S. M.; Martin, D. J.; Smith, H. M.; Morris, P. T.; Cook, A. K. Modular Ni(0)/Silane Catalytic System for the Isomerization of Alkenes. *Organometallics* **2022**, *41* (4), 486–496. https://doi.org/10.1021/acs.organomet.2c00010.

(16) Gilman, H.; Aoki, D. Reactions of Triphenyisilyllithium with 1-Chloropropene and l-Chloro-1-Butene. *J. Organometal.Chem.* **1964**, 89–92.

(17) Barberot, A.; Cuadradot, P.; Fleming, I.; Gonzalezt, A. M.; Pulido, F. J.; Sanchez, A. *Synthesis of Vinylsilanes by Silyl-Cupration of Acetylenes Using Tert-but y Ldiphenylsil y I-Cupr at e Reagents*; 1995.

(18) Kobayashi, T.; Yorimitsu, H.; Oshima, K. Cobalt-Catalyzed Isomerization of 1-Alkenes to (E)-2-Alkenes with Dimethylphenylsilylmethylmagnesium Chloride and Its Application to the Stereoselective Synthesis of (E)-Alkenylsilane. *Chem Asian J* **2009**, *4* (7), 1078–1083. https://doi.org/10.1002/asia.200900111.

(19) Zhang, Q.; Wang, S.; Yin, J.; Xiong, T.; Zhang, Q. Remote Site-Selective Asymmetric Protoboration of Unactivated Alkenes Enabled by Bimetallic Relay Catalysis. *Angewandte Chemie - International Edition* **2022**, *61* (23). https://doi.org/10.1002/anie.202202713.

(20) Dethe, D. H.; Beeralingappa, N. C.; Das, S.; Nirpal, A. K. Ruthenium-Catalyzed Formal Sp3C-H Activation of Allylsilanes/Esters with Olefins: Efficient Access to Functionalized 1,3-Dienes. *Chem Sci* **2021**, *12* (12), 4367–4372. https://doi.org/10.1039/d0sc06845d.

(21) Chen, C.; Dugan, T. R.; Brennessel, W. W.; Weix, D. J.; Holland, P. L. Z -Selective Alkene Isomerization by High-Spin Cobalt(II) Complexes. *J Am Chem Soc* **2014**, *136* (3), 945–955. https://doi.org/10.1021/ja408238n.

(22) Ichinose, Y.; Nozaki, K.; Wakamatsu, K.; Oshima, K.; Utimoto, K. *Induced Stereoselective Radical Addition of Ph3GeE to Acetylenes and Its Application to Isomerization of Olefins*; 1987; Vol. 28.

(23) Albright, H.; Vonesh, H. L.; Schindler, C. S. Superelectrophilic Fe(III)-Ion Pairs as Stronger Lewis Acid Catalysts for (E)-Selective Intermolecular Carbonyl-Olefin Metathesis. *Org. Lett.* **2020**, *22* (8), 3155–3160. https://doi.org/10.1021/acs.orglett.0c00917.

(24) Yang, B.; Wang, Z. X. Nickel-Catalyzed Alkylation or Reduction of Allylic Alcohols with Alkyl Grignard Reagents. *Journal of Organic Chemistry* **2020**, *85* (7), 4772–4784. https://doi.org/10.1021/acs.joc.0c00008.

(25) Simonetti, S. O.; Larghi, E. L.; Kaufman, T. S. A Facile and Convenient Sequential Homobimetallic Catalytic Approach towards β-Methylstyrenes. A One-Pot Stille Cross-Coupling/Isomerization Strategy. *Org. Biomol. Chem.* **2014**, *12* (22), 3735–3743. https://doi.org/10.1039/c4ob00604f.

(26) Denmark, S. E.; Werner, N. S. Cross-Coupling of Aromatic Bromides with Allylic Silanolate Salts. *J. Am. Chem. Soc.* **2008**, *130* (48), 16382–16393. https://doi.org/10.1021/ja805951j.

(27) Mariampillai, B.; Herse, C.; Laufens, M. Intermolecular Heck-Type Coupling of Aryl Iodides and Allylic Acetates. *Org Lett* **2005**, *7* (21), 4745–4747. https://doi.org/10.1021/ol051947e.

(28) Kustiana, B. A.; Elsherbeni, S. A.; Linford-Wood, T. G.; Melen, R. L.; Grayson, M. N.; Morrill, L. C. B(C 6 F 5) 3 -Catalyzed E -Selective Isomerization of Alkenes . *Chemistry – A European Journal* **2022**. https://doi.org/10.1002/chem.202202454.

(29) Liu, H.; Xu, M.; Cai, C.; Chen, J.; Gu, Y.; Xia, Y. Cobalt-Catalyzed Z to e Isomerization of Alkenes: An Approach to (E)-β-Substituted Styrenes. *Org. Lett.* **2020**, *22* (3), 1193–1198. https://doi.org/10.1021/acs.orglett.0c00072.

(30) Taniguchi, M.; Oshima, K.; Utimoto, K. *Formation Of Alkylated Tryphenylsilylalkenes From The Reaction Of Triphenylsilyl-Substituted Oxiranyl Anion With Organoaluminium Reagents*; 1991; Vol. 32.
